# Effect of *H2A*.*Z* deletion is rescued by compensatory mutations in *Fusarium graminearum*

**DOI:** 10.1371/journal.pgen.1009125

**Published:** 2020-10-22

**Authors:** Zhenhui Chen, Enric Zehraoui, Anna K. Atanasoff-Kardjalieff, Joseph Strauss, Lena Studt, Nadia Ponts

**Affiliations:** 1 INRAE, MycSA, Villenave d’Ornon, France; 2 Department of Applied Genetics and Cell Biology, University of Natural Resources and Life Sciences, Vienna (BOKU), Vienna, Austria; Universita degli Strudi di Roma La Sapienza, ITALY

## Abstract

Fusarium head blight is a destructive disease of grains resulting in reduced yields and contamination of grains with mycotoxins worldwide; *Fusarium graminearum* is its major causal agent. Chromatin structure changes play key roles in regulating mycotoxin biosynthesis in filamentous fungi. Using a split-marker approach in three *F*. *graminearum* strains INRA156, INRA349 and INRA812 (PH-1), we knocked out the gene encoding H2A.Z, a ubiquitous histone variant reported to be involved in a diverse range of biological processes in yeast, plants and animals, but rarely studied in filamentous fungi. All Δ*H2A*.*Z* mutants exhibit defects in development including radial growth, sporulation, germination and sexual reproduction, but with varying degrees of severity between them. Heterogeneity of osmotic and oxidative stress response as well as mycotoxin production was observed in Δ*H2A*.*Z* strains. Adding-back wild-type *H2A*.*Z* in INRA349Δ*H2A*.*Z* could not rescue the phenotypes. Whole genome sequencing revealed that, although *H2A*.*Z* has been removed from the genome and the deletion cassette is inserted at *H2A*.*Z* locus only, mutations occur at other loci in each mutant regardless of the genetic background. Genes affected by these mutations encode proteins involved in chromatin remodeling, such as the helicase Swr1p or an essential subunit of the histone deacetylase Rpd3S, and one protein of unknown function. These observations suggest that *H2A*.*Z* and the genes affected by such mutations are part or the same genetic interaction network. Our results underline the genetic plasticity of *F*. *graminearum* facing detrimental gene perturbation. These findings suggest that intergenic suppressions rescue deleterious phenotypes in Δ*H2A*.*Z* strains, and that *H2A*.*Z* may be essential in *F*. *graminearum*. This assumption is further supported by the fact that *H2A*.*Z* deletion failed in another *Fusarium* spp., *i*.*e*., the rice pathogen *Fusarium fujikuroi*.

## Author summary

The function of histone variant H2A.Z has been widely studied in *Saccharomyces cerevisiae* and higher eukaryotes. However, there is a significant lack of knowledge of the role of H2A.Z and its regulation in filamentous fungi. Here we demonstrate for the first time that deleterious consequences of complete removal of *H2A*.*Z* from the genome of *F*. *graminearum* are mitigated by genetic suppression. The suppressors detected open new possibilities to investigate in more depth the network linking H2A.Z and other genes in *F*. *graminearum* and used to decipher unresolved questions concerning H2A.Z.

## Introduction

*Fusarium graminearum*, a homothallic filamentous fungus, is the major causal agent responsible for the devastating disease Fusarium head blight (FHB) of wheat, barley and other small grain cereal crops worldwide [1,2]. In wheat, FHB affects the head with symptoms of wilted kernels, reducing yield at harvest and causing billions of dollars losses [3,4]. As an additional serious concern, the presence of *F*. *graminearum* in kernels results in the contamination of cereals with mycotoxins, especially the extremely stable type B trichothecenes (TCTB) including deoxynivalenol (DON) and its acetylated C-15 derivatives (15-ADON). The presence of these mycotoxins on cereals, persistent in derived food and feed products, threatens the health of humans and animals [5–7]. A growing body of evidence indicates that chromatin structure changes play a critical role in the regulation of mycotoxin biosynthesis in filamentous fungi.

In eukaryotes, gene expression depends on chromatin structure. Chromatin is the fundamental packaging form of DNA, which is wrapped around an octamer of canonical core histones H2A, H2B, H3 and H4 to form nucleosomes [8,9]. Canonical histones are conserved across eukaryotic species and represent the major part of histones within an organism. The N-terminal tails of histones are extensively marked by covalent post-translational modifications (PTMs) including methylation, phosphorylation, or acetylation for example. The combinatorial positioning of these histone marks impact gene expression by altering chromatin state according to a not yet deciphered “Histone Code” [10]. Additionally, non-allelic isoforms of canonical histones called histone variants also exist in all eukaryotes [11,12]. The H2A family encompasses the largest number of variants [13]; H2A.Z is considered as the most evolutionarily conserved one [14,15]. Generally, H2A.Z and H2A have ~60% sequence similarity. It differs from H2A in the increased acidic patch and the carboxy-terminal α-helix included in the docking domain, which is a structure involved in the interaction of the H2A-H2B dimer with the (H3-H4)_2_ tetramer [16,17]. H2A.Z has been identified in various species, including *Arabidopsis thaliana* [18], *Saccharomyces cerevisiae* [19], *Drosophila melanogaster* [20], and human [21]. It has been found involved in a diverse range of biological processes, including genome stability [22–24], DNA repair [25–28] and transcriptional regulation [29–31]. The absence of H2A.Z may be lethal in many organisms such as mouse [32], Drosophila [20], frogs [33] and Tetrahymena [34], but not in *S*. *cerevisiae* [35,36]. The dynamic process of H2A.Z deposition/removal from nucleosomes is mediated by ATP-dependent chromatin remodeling complexes, especially complexes belonging to the SNF2 superfamily [37,38]. In *S*. *cerevisiae*, the complex SWR1 is involved in the recruitment of H2A.Z. It can replace the H2A-H2B by H2A.Z-H2B dimers. By contrast, the removal of H2A.Z from nucleosomes is mediated by the SWR1-related Inositol requiring 80 (INO80) complex [28,39,40]. INO80 shares several subunits with SWR1 complex and its functions are usually associated with DNA double-strand breaks repair [41,42]. Homologs for H2A.Z and most subunits of yeast SWR1 and INO80 complex can be found in *F*. *graminearum*, meaning that similar biological processes may also occur [43].

Various studies illustrated that functions of H2A.Z are based on its collaboration with other histone marks, particularly those that decorate histone H3 tails. For example, both in human and mouse ES cells, H2A.Z is enriched at enhancers and promoters marked by H3K4me3, a mark that activates gene expression [44,45]. Moreover, it seems that H2A.Z can act as a functional substitute for H3K9me3 in chromatin, recruiting Heterochromatin Protein 1 (HP1) when H3K9me3 levels are low for example [46]. Still in mouse ES cells, H2A.Z strongly co-localizes with H3K27me3 and Polycomb Repressive Complexes (PRCs) 1 and 2 near the transcription start site (TSS) of genes involved in cell differentiation, and helps to keep these genes silenced [47]. In filamentous fungi, major roles of chromatin structure changes by means of histone marks in regulating the biosynthesis of mycotoxins and other secondary metabolites have been evidenced. For example, in *F*. *graminearum*, H3K4me3 deposited by FgSet1 is required for the active transcription of genes involved in the biosynthesis of both DON and the pigment aurofusarin [48]. Consistently, lack of the COMPASS component FgCcl1, required for full H3K4me3, also results in reduced DON levels whereas aurofusarin levels remained unaffected [49]. Notably, removal of the H3K4me3-specific histone demethylase FgKdm5 also resulted in decreased DON levels, suggesting more complex regulatory circuits than previously anticipated [50]. By contrast, the histone mark H3K27me3 represses 14% of *F*. *graminearum*’s genome, including genes involved in secondary metabolism [51]. However, regarding the function of H2A.Z in fungi, very few studies were carried out in *Neurospora crassa* studying specifically the function of H2A.Z and identifying a role in oxidative stress response [52,53] as well as a link with the deposition of H3K27me3 [54].

Here, we hypothesized that H2A.Z plays important roles in controlling development and metabolism in two *Fusarium* spp., *i*.*e*., *F*. *graminearum* and *F*. *fujikuroi*. Using a reverse genetics approach on three different strains of each species, we provide pieces of evidence that H2A.Z may be essential in both species. Not a single homokaryotic Δ*H2A*.*Z* strain was recovered from *F*. *fujikuroi* wild-type strains, whereas mutants in which *H2A*.*Z* has been totally removed from the genome were obtained in all three *F*. *graminearum* strains. However, compensatory mutations occurred at other sites in FgΔ*H2A*.*Z* mutants of different genetic backgrounds, revealing an unsuspected genome plasticity. As a whole, our results suggest a flexible network of genes that, by interacting with H2A.Z within the same functional network, can re-wire itself to rescue deleterious phenotypes.

## Results and discussion

### Identification of FgH2A.Z as a single H2A variant in *F*. *graminearum*

We scanned the entire proteome of *F*. *graminearum* PH-1 for the presence of proteins carrying a domain characteristic of the C-terminal end of histone H2A, and found the accessions FGRAMPH1_01T26109 and FGRAMPH1_01T03973 as candidates for histone H2A and one variant. We putatively assigned FGRAMPH1_01T26109 as H2A and FGRAMPH1_01T03973 as H2A.Z by protein sequence similarity (see blastp [55] reports in S1 File and S2 File). *FgH2A* and *FgH2A*.*Z* are found on chromosomes 1 and 4, respectively. Gene models supported by transcriptomics data [56–58] show that both genes possess two introns, the first one being much larger in FgH2A.Z than FgH2A for which the second exon is larger (S1A Fig). As a point of comparison, the other sordariomycete *Neurospora crassa* was shown to also possess two introns in its H2A gene [59] whereas the eurotiomycete *Aspergillus nidulans* was shown to possess three ones [60]. Similarly, H2A.Z also possesses two introns in these two last species. We further examined available gene expression data obtained for asexual spores and actively growing *F*. *graminearum* mycelia [58]. Important changes in expression levels can be observed in *FgH2A* (more than five-fold, S1B Fig) but not for *FgH2A*.*Z* suggesting that, similarly to other eukaryotes [61], the expression of H2A is restricted to actively dividing cells. Finally, protein alignment found ~65% sequence identity between FgH2A and FgH2A.Z (S1C Fig), with notably the typical H2A.Z features of an extended acidic patch and the substitution of a glutamine for a glycine at position 104 responsible for a less stable interaction between H2A.Z with H3 than H2A with H3 [17]. All together, these elements indicate that FGRAMPH1_01T03973 is a *bona fide* histone H2A.Z in *F*. *graminearum*.

### Knock-out mutant of Fg*H2A*.Z is viable with severe defects in development, toxin production, and stress response

**Fig 1:**
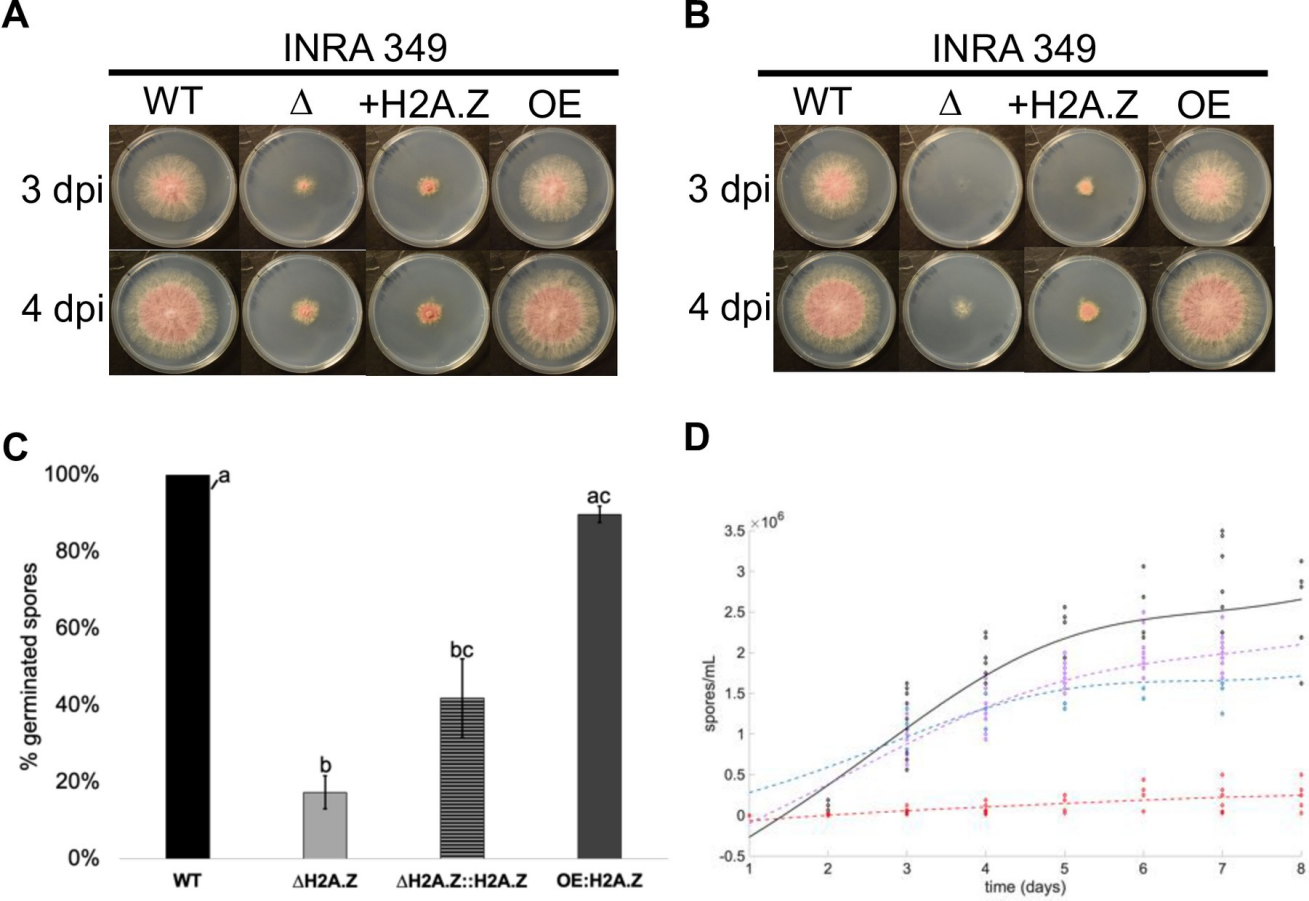
Developmental defects in INRA349 mutants. **(A)** and **(B).** Radial growth in FgINRA349 wild-type, Δ*H2A*.*Z*, Δ*H2A*.*Z*::*H2A*.*Z*, and OE:H2A.Z grown from a central 3 mm-diameter plug **(A)** or 100 spores **(B)** on CM agar for three and four days at 25°C in the dark. WT = wild-type I349; Δ = deletion mutant I349Δ*H2A*.*Z*; +H2A.Z = I349Δ*H2A*.*Z*::*H2A*.*Z* mutant; OE = I349OE:*H2A*.*Z*; dpi = days post-inoculation. **(C).** Germination rates of FgINRA349 wild-type, Δ*H2A*.*Z*, Δ*H2A*.*Z*::*H2A*.*Z*, and OE:H2A.Z after eight hours of incubation. **(D).** Fitted kinetics of sporulation followed for eight days. Black = wild-type I349; dashed red = I349Δ*H2A*.*Z*; dashed blue = I349Δ*H2A*.*Z*::*H2A*.*Z* mutant; dashed purple = I349OE:*H2A*.*Z*. For **(C)** and **(D)**, letters indicate statistically significant curve groups after Kruskal-Wallis testing and Tukey-Kramer correction for multiple testing (*p* < 0.05).

To explore H2A.Z functions in *F*. *graminearum*, we proceeded with gene knock out experiments to remove the full coding sequence of FGRAMPH1_01T03973 (see Materials and Methods, and S2 Fig) from *F*. *graminearum* strain INRA349 known to be highly virulent on wheat and produce deoxynivalenol and its 15-acetylated form [62,63]. We successfully obtained one mutant I349Δ*H2A*.*Z* (verified by both PCR and southern blot, S3 Fig), suggesting that the deletion of *H2A*.*Z* in *F*. *graminearum* is non-lethal. This result is consistent with previous experiments in fungi that found H2A.Z as non-essential in *N*. *crassa* [64], *S*. *cerevisiae* [36], or *Schizosaccharomyces pombe* [65] for example, although they exhibited reduced growth in the deletion mutants. Our *F*. *graminearum* Δ*H2A*.*Z* mutant was indeed very slow-growing compared to its respective wild-type parental strain (**Fig 1A**). Strikingly, the add-back mutant I349Δ*H2A*.*Z*::*H2A*.*Z*, restoring *H2A*.*Z* expression levels to those observed in wild-type strain (see Materials and Methods, S4A Fig and S4C Fig), did not exhibit a rescued phenotype. This observation indicates that, although the single copy of *H2A*.Z was successfully removed from the genome, the observed effects on radial growth could not be (at least fully) attributed to the loss of H2A.Z. We also engineered a version of INRA349 in which we inserted a copy of *FgH2A*.*Z* under the control of the constitutive promoter *pGPD* (see Materials and Methods, and S4B Fig) and over-expressing *FgH2A*.Z by 7.6-fold (S4C Fig), here referred to as I349-OE::*H2A*.*Z*). No effect of radial growth could be observed in our conditions (**Fig 1A**).

The same experiment was repeated inoculating culture plates with 100 spores rather than a plug from a precedent culture. Similarly to our previous observation using plugs, I349-OE::*H2A*.*Z* showed similar growth to that of wild type, and growth defect in I349Δ*H2A*.*Z* compared to wild type was drastic (**Fig 1B**). However, the add-back mutant I349Δ*H2A*.*Z*::*H2A*.*Z* seemed to perform better and showed better ability to grow than I349Δ*H2A*.*Z*, although wild-type phenotype was not fully restored. We thus hypothesized that germination may be affected differently in I349Δ*H2A*.*Z* and I349Δ*H2A*.*Z*::*H2A*.*Z*. The kinetics of spore germination was monitored for up to eight hours in INRA349 wild type, I349Δ*H2A*.*Z*, I349Δ*H2A*.*Z*::*H2A*.*Z*, I349-OE::*H2A*.*Z*. Results are shown **Fig 1C**. In INRA349 wild type, germination was complete after eight hours, an observation consistent with a previously published assay using the same strain [63]. Similar results were obtained for I349-OE::*H2A*.*Z*, with 90% (± 2) of the spores germinated after eight hours. However, within the same time span, less than 20% of the spores of I349Δ*H2A*.*Z* (17% ± 4) and 42% (± 10) I349*ΔH2A*.*Z*::*H2A*.*Z* had germinated, values significantly lower than those measured for both INRA 349 wild type and I349-OE::*H2A*.*Z*. Germination in I349Δ*H2A*.*Z*::*H2A*.*Z* was higher than the one measured for I349Δ*H2A*.*Z*, although not significant (certainly due to high deviation in measurements). Considering this trend, we hypothesize that the differences in radial growth obtained above are caused by a better ability of I349Δ*H2A*.*Z*::*H2A*.*Z* to germinate and actually start growing.

We further tested the ability of our mutant strains to produce asexual spores (macroconidia). Kinetics of conidia production in a spore-inducing medium was followed for up to eight days for I349 wild-type, I349Δ*H2A*.*Z*, I349Δ*H2A*.*Z*::*H2A*.*Z*, and I349-OE::*H2A*.*Z* strains. Results are shown **Fig 1D**. Deletion of *H2A*.*Z* severely impeded sporulation, but phenotype was rescued in the add-back mutant I349Δ*H2A*.*Z*::*H2A*.*Z* to levels not differing significantly from those measured in I349 wild-type and I349-OE::*H2A*.*Z* strains. Here, while I349Δ*H2A*.*Z*::*H2A*.*Z* had its ability to produce asexual spores restored, its ability to initiate germination is altered.

**Fig 2:**
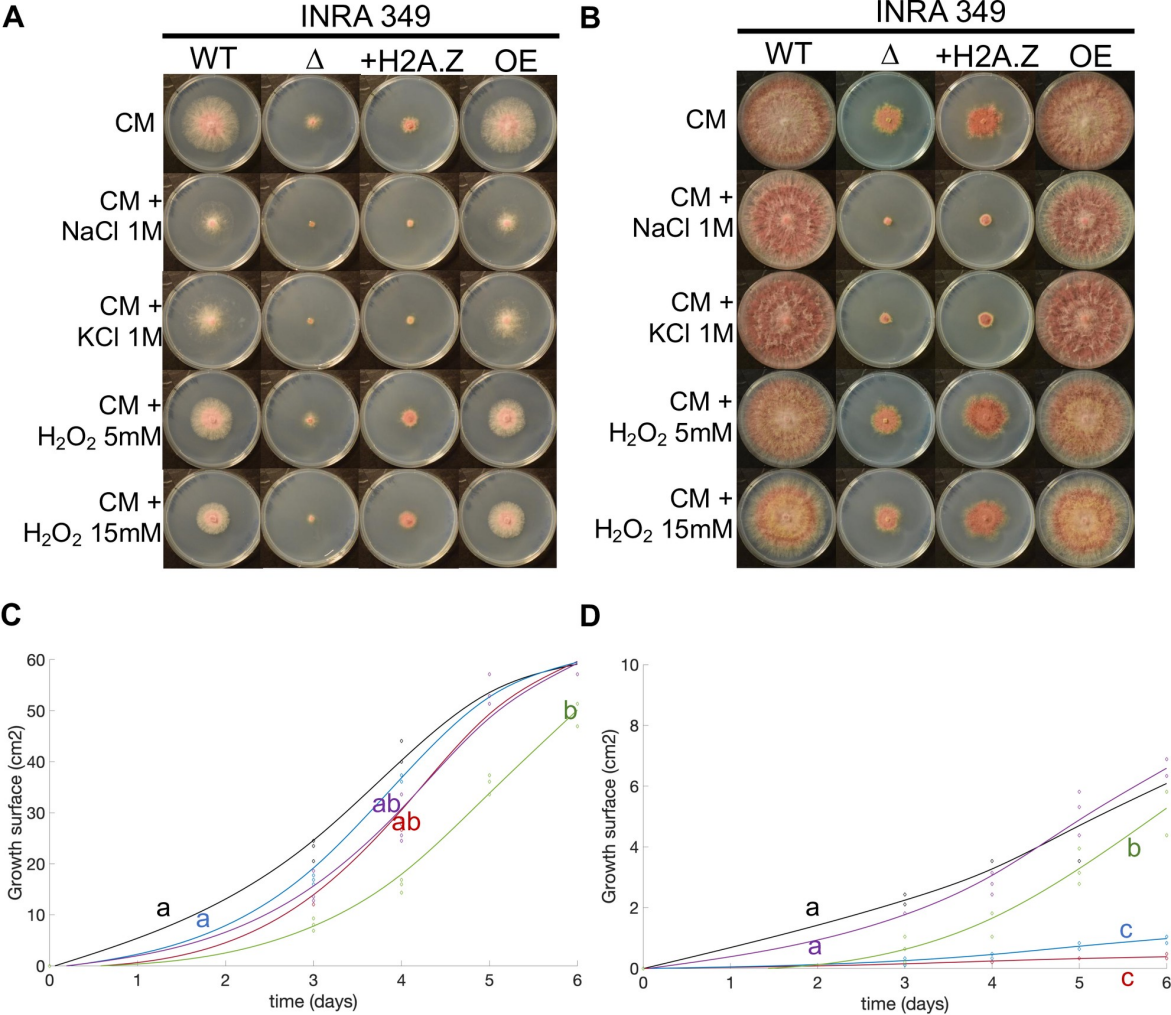
Abiotic stress resistance of INRA349 and its mutants. **(A)** and **(B)** Radial growth in FgINRA349 wild-type, Δ*H2A*.*Z*, Δ*H2A*.*Z*::*H2A*.*Z*, and OE:H2A.Z grown from a central 3 mm-diameter plug on CM agar supplemented with NaCl 1M, KCl 1M, H_2_O_2_ 5 mM, H_2_O_2_ 15 mM, or not supplemented for three **(A)** and six **(B)** days at 25°C in the dark. WT = wild-type I349; Δ = I349Δ*H2A*.*Z*; +H2A.Z = I349Δ*H2A*.*Z*::*H2A*.*Z*; OE = I349OE:*H2A*.*Z***. (C)** and **(D).** Fitted radial growth kinetics followed for six days for wild-type I349 **(C)** and I349Δ*H2A*.*Z* **(D)**. Black = CM; red = CM + NaCl 1M; blue = CM + KCl 1M; purple = CM + H_2_O_2_ 5 mM; green = CM + H_2_O_2_ 15 mM. Letters indicate statistically significant curve groups after Kruskal-Wallis testing and Tukey-Kramer correction for multiple testing (*p* < 0.05).

We pursued our exploration and investigated the ability to respond to osmotic/ionic stress and oxidative stress, which are common and major stresses for fungi [66]. We thus cultured for up to six days our INRA349 wild-type and mutant strains on CM agar plates supplemented with NaCl 1M, KCl 1M, H_2_O_2_ 5 mM, or H_2_O_2_ 15 mM, or not supplemented. Results are shown **Fig 2** and S5 Fig. In terms of radial growth, wild-type strain showed little sensitivity to KCl but increased with NaCl (Fig 2A and 2B, S5A Fig, S5B Fig), although not significant over the whole growth curve (**Fig 2C**). Similarly, H_2_O_2_ had important measurable effects when applied at 15 mM rather than 5 mM (**Fig 2A, 2B and 2C**). Nonetheless, both NaCl and KCl caused visible discoloration of the mycelium and reduction in aerial mycelium (**Fig 2B**). Similar effects were observed for I349-OE::*H2A*.*Z*, although the effects of NaCl and H_2_O_2_ 5 mM were this time significant (up to 6 dpi where growth curves meet, S5A Fig, S5B Fig and S5C Fig). Regarding I349Δ*H2A*.*Z*, growth curves showed different trends (**Fig 2D**). Treatment with NaCl and KCl both have drastic effects on growth, whereas H_2_O_2_ 5 mM or 15 mM showed no or moderate effects respectively. The situation is exacerbated in the add-back mutant I349Δ*H2A*.*Z*::*H2A*.*Z* that shows no sign of sensitivity to H_2_O_2_ 5 mM or 15 mM (S5A Fig, S5B Fig and S5D Fig).

**Fig 3:**
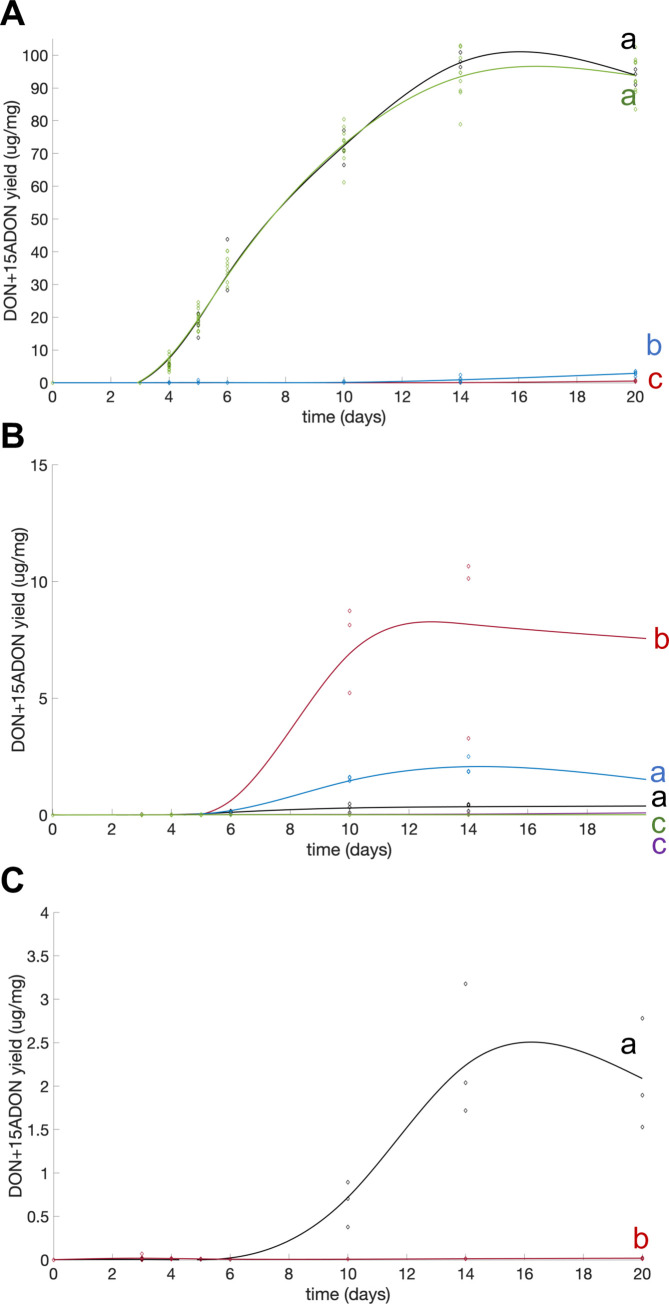
Fitted kinetics of production of DON and 15-ADON by INRA349, INRA156, PH-1, and their respective mutants grown in liquid MS cultures. **(A)** FgINRA349 wild-type (black), Δ*H2A*.*Z* (red), Δ*H2A*.*Z*::*H2A*.*Z* (blue), and OE:H2A.Z (green)**. (B)** FgINRA156 wild-type (black) and its four Δ*H2A*.*Z*, numbered #1 to #4 (red, blue, purple, and green, respectively)**. (C)** PH-1 wild-type (black) and its Δ*H2A*.*Z* mutant (red). Toxin yields are expressed in micrograms of toxins per milligram of dry biomass. Letters indicate statistically significant curve groups after Kruskal-Wallis testing and Tukey-Kramer correction for multiple testing (*p* < 0.05).

The ability to produce mycotoxins was finally investigated in all our mutants and compared to their respective wild-type strains. INRA349 wild type can produce high amounts of DON (and its derivative 15-ADON *in vitro*, [62,63]), a virulence factor that helps the fungus to spread within wheat spikes [67]. We monitored the kinetics of DON+15-ADON production of wild type and mutants for all strains and expressed yields as micrograms of mycotoxins produced per milligram of dry weight of mycelium (**Fig 3A**). Toxin production could be detected after three days of culture post-inoculation (dpi) in our conditions for both INRA349 wild type and over-expressed mutant I349-OE::*H2A*.*Z*. Both strains then produced toxins exponentially until 14 days of culture after which toxin accumulation seemed to stabilize. Both curves were nearly identical. On the contrary, we detected toxins in cultures of I349Δ*H2A*.*Z* and I349Δ*H2A*.*Z*::*H2A*.*Z* only after four days, and production remained very low throughout. Nonetheless, DON and 15-ADON production seemed to slightly increase between 12 and 14 days of culture and thereafter in I349Δ*H2A*.*Z*::*H2A*.*Z* but not in I349Δ*H2A*.*Z*, significantly.

### Putative intergenic suppressors rescue deleterious phenotypes in I349Δ*H2A*.*Z* and I349Δ*H2A*.*Z*::*H2A*.*Z* strains

The phenotypes presented above were puzzling. Indeed, although I349Δ*H2A*.*Z*::*H2A*.*Z* had successfully re-integrated *H2A*.*Z* at its *locus*, most deficiencies caused by *H2A*.*Z* deletion could not be rescued. Thus, in order to shed some light on the causes for our observations, INRA349 wild-type, Δ*H2A*.*Z*, Δ*H2A*.*Z*::*H2AZ*, and OE::*H2A*.*Z* strains were sequenced by Whole Genome Sequencing (WGS) and all subsequent genome sequences scrutinized. As expected, sequencing confirmed that *H2A*.*Z* coding sequence was absent from all Δ*H2A*.*Z* strains and successfully added back in I349Δ*H2A*.*Z*::*H2A*.*Z* (S6A Fig). More surprising, we discovered in I349Δ*H2A*.*Z* one mutation elsewhere in the genome (S6B Fig) and a second one in I349Δ*H2A*.*Z*::*H2A*.*Z* (S6C Fig; verified by Sanger sequencing, S7 Fig), which we hypothesized to be compensatory mutations considered to have beneficial effects on fitness when a deleterious mutation is present [68].

**Fig 4:**
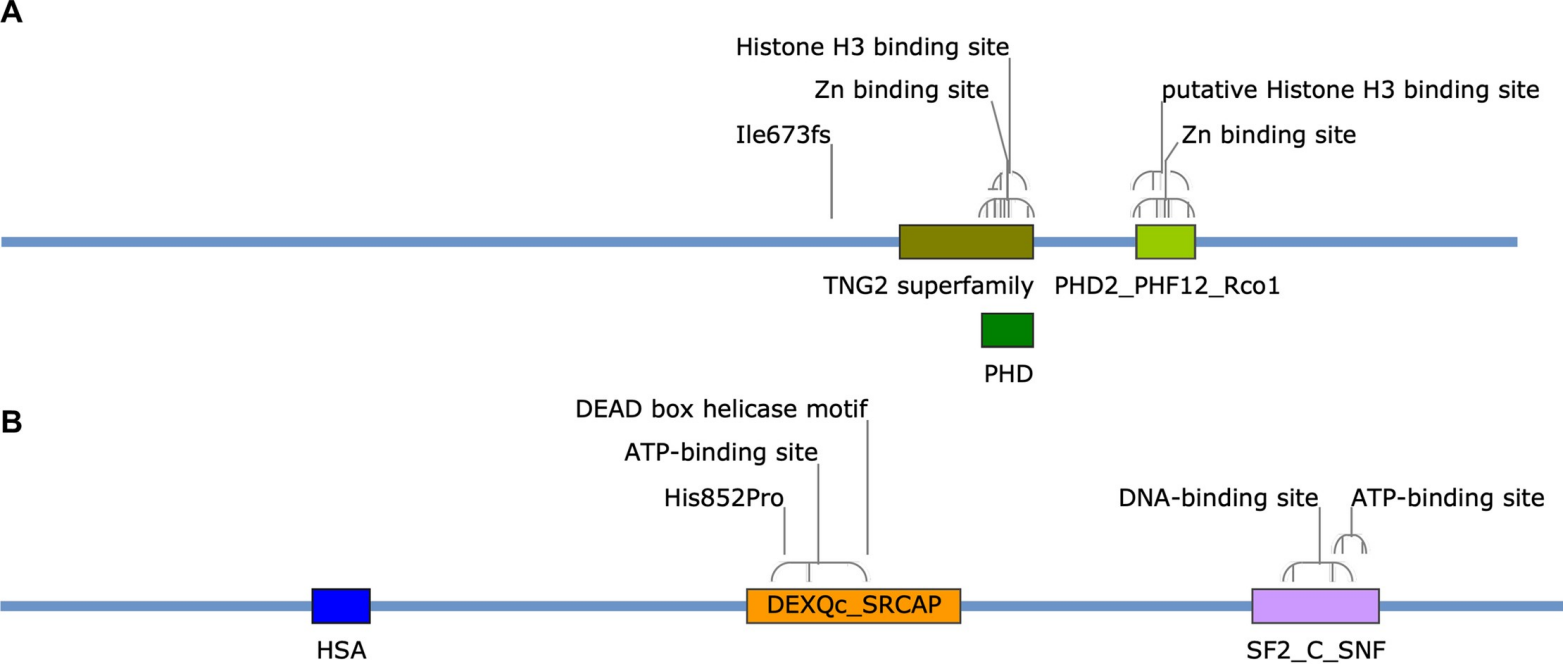
Domain architecture of proteins affected by compensatory mutations in I349Δ*H2A*.*Z* and I349Δ*H2A*.*Z*:*H2A*.*Z*. **(A)** Essential subunit of Rpd3S complex (FGRAMPH1_01G23597; 1,225 aa). The annotation “Ile673fs” indicates the position of a frameshift starting at position 673 of the protein in I349Δ*H2A*.*Z* (an isoleucine in wild-type). **(B)** Swr1 (FGRAMPH1_01G18675; 1,691 aa). The annotation “His852Pro” indicates the replacement of a histidine at position 852 by a proline in I349Δ*H2A*.*Z*:*H2A*.*Z*. Domain accession numbers in NCBI CDD/SPARCKLE database: TNG2 superfamily = CL34876, PHD = CL22851, PHD2_PHF12_Rco1 = CD15534, HAS = PFAM07529, DEXQc_SRCAP = CD18003, SF2_C_SNF = CD18793. Architectures are displayed with SnapGene Viewer 5.0.6.

The mutation discovered in I349Δ*H2A*.*Z* is a deletion of a single nucleotide A in position 2015 of its CDS (S5B Fig), causing a frameshift mutation (Ile673fs) in the ORF of FGRAMPH1_01G23597, annotated in the FungiDB database as an essential subunit of the histone deacetylase Rpd3S complex. FGRAMPH1_01G23597 encodes a 1225 amino acid-long protein predicted to contain two PHD-type domains (one of the TNG2 superfamily and one PHD2_PHF12_Rco1 particularly found in the yeast Rpd3s component Rco1p) including their zinc and histone H3 binding sites (**Fig 4A**) using the NCBI CD-Search [69,70]. Provided corresponding transcripts with such premature termination codon is not degraded by the non-sense-mediated mRNA decay (NMD) pathway, resulting in no protein at all, the frameshift mutation that occurred in I349Δ*H2A*.*Z* would have the drastic consequence of removing both PHD domains and creating a much shorter protein of 783 amino acids (**Fig 4A** and S8 Fig). Such truncated protein may either be degraded by the unfolded protein response (UPR) pathway or see its functions very likely dramatically impaired. We hypothesize that this mutation could be responsible for rescuing the deleterious effects of *H2A*.*Z* deletion, otherwise lethal. Rdp3S is typically involved in the deacetylation of H3K36. Functions of the acetylated *vs*. methylated H3K36 are multiple and the subject of intense investigation (reviewed in [71]); methylation of H3K36 is typically associated with active transcription. A proposed mechanism is that SET2-mediated methylation of H3K36 towards the 3’ end of genes mediates general histone deacetylation through the recruitment of Rpd3s to slow down transcription elongation at the end of genes [72]. In mammalian embryos, it was suggested that deposition of acetylated H2A.Z (a mark of active transcription) and methylated H3K36 are precisely orchestrated to allow fine-tuned expression of developmental genes and prevent spurious transcription [73]. Here, a frameshift mutation in FGRAMPH1_01G23597 with the likely consequence of losing its activity seems to counter-balance to some extent (the strain can survive) the deletion of *H2A*.*Z*. A possible explanation for this rescue could be that transcription elongation speed increases at the end of genes to provide partial compensation for the loss of H2A.Z, in other words the loss of the activation counterpart of a fine-tuned process. In this case, however, fine regulation of transcription can no longer be assured and survival is only permitted at high cost.

In the add-back mutant I349Δ*H2A*.*Z*::*H2A*.*Z*, in addition to the frameshift mutation in FGRAMPH1_01G23597 inherited from its parental strain I349Δ*H2A*.*Z*, another mutation was found in the gene FGRAMPH1_01G18675 encoding the 1,691 amino acid-long helicase Swr1p (S6C Fig) *i*.*e*., the catalytic subunit of the SWR1 complex involved in the deposition of H2A.Z (see [39] for a review). This mutation caused the exchange of a histidine for a proline at position 852, within the domain DEXQc_SRCAP (**Fig 4B**). Histidine is often seen as one of the most versatile and reactive amino acid for protein interactions, reacting in numerous ways to create various types of molecular interactions [74]. Proline is also a peculiar amino acid but with very different properties; when incorporated, it can disrupt secondary structure due to a unique locked-in conformation of its side chain [75]. An effect on the protein activity is here likely considering its insertion inside the stretch of amino acids composing the ATP-binding site (**Fig 4B**). Considering the scenario hypothesized above regarding the consequences of losing the activity of Rpd3s essential subunit, adding back *H2A*.*Z* in such background may cause transcription to be spuriously activated in the absence of Rpd3S fine tune-down system. In this system, a mutation in Swr1p that would impede the incorporation of H2A.Z in nucleosomes may moderate transcription activation and somehow re-equilibrate transcriptional balance to prevent uncontrolled transcription.

### Compensatory mutations with varying effects are obtained when *H2A*.*Z* is deleted in different genetic background

**Fig 5:**
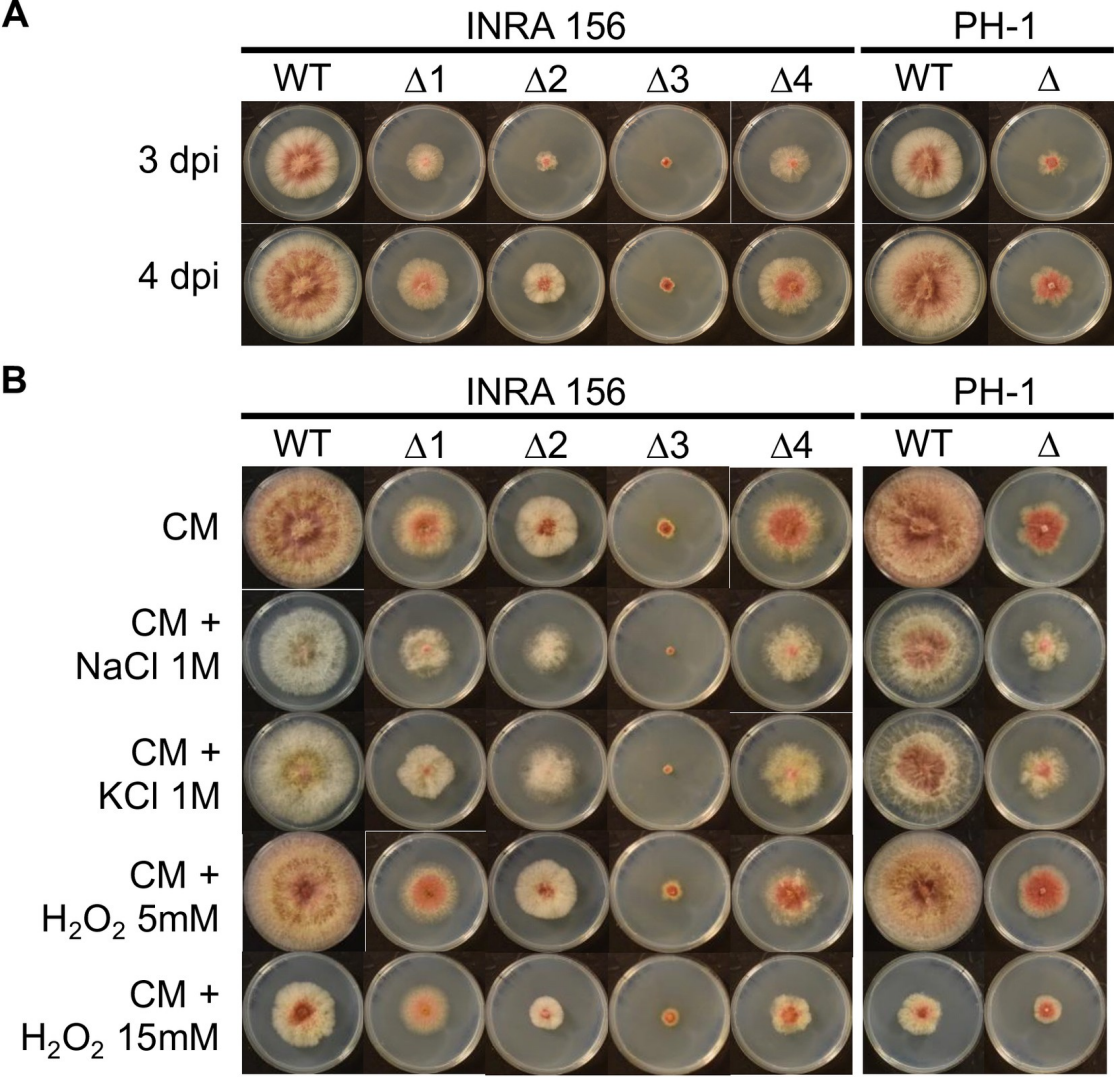
Growth defects in INRA156 and PH-1 mutants. WT = wild-type; Δ = deletion mutants Δ*H2A*.*Z* (numbered 1 to 4 for those obtained in INRA156 background); dpi = days post-inoculation. **(A).** Radial growth in FgINRA156 and FgPH-1 wild-type, and their Δ*H2A*.*Z* mutants, grown from a central 3 mm-diameter plug on CM agar for three and four days at 25°C in the dark. **(B).** Effect of abiotic stresses on radial growth in FgINRA156 and FgPH-1 wild-type and their Δ*H2A*.*Z* mutants, grown from a central 3 mm-diameter plug on CM agar supplemented with NaCl 1M, KCl 1M, H_2_O_2_ 5 mM, H_2_O_2_ 15 mM, or not supplemented for six days at 25°C in the dark.

Compensatory mutations are defined as beneficial in a particularly deleterious context, and are otherwise undesirable as they usually come as the cost to at least some fitness. Since genetic background influences the outcome of compensatory mutations [76], we engineered Δ*H2A*.*Z* strains in two additional genetic backgrounds of *F*. *graminearum*, INRA156 [77] and the reference strain PH-1 [78–80], and successfully obtained four deletion mutants and one mutant in each of the strain, respectively. All Δ*H2A*.*Z* deletion strains in INRA156 and PH-1 were severely impacted in their development, but with varying degrees of severity (**Fig 5A**). The mutant strain I156Δ*H2A*.*Z*#3 shows the most extreme growth defect, all strains considered. The fact that four mutant strains originating from the same parental strain INRA156 exhibit different phenotypes suggests that the suppressor mutations rather than the influence of the genetic background are responsible for the observed phenotypic variations. Regarding response to oxidative and osmotic/ionic stresses, both INRA156 and PH-1 are the most sensitive to H_2_O_2_ 15 mM, and to a much lesser extent NaCl 1M, in terms of radial growth (**Fig 5B,** S9A Fig and S9F Fig). Nonetheless, both NaCl and KCl caused striking discoloration of the mycelium of wild-type strains (**Fig 5B**). This discoloration can also be observed in Δ*H2A*.*Z* strains submitted to stress (**Fig 5B**). Nonetheless, marked differences in radial growth sensitivity could be observed. While sensitivity hierarchy is identical in I156Δ*H2A*.*Z*#4 and PH-1Δ*H2A*.*Z* to the one in their wild-type counterparts (*i*.*e*., mostly to H_2_O_2_ 15 mM followed by NaCl 1M, S9E Fig and S9G Fig), the growth curves of I156Δ*H2A*.*Z*#1 and I156Δ*H2A*.*Z*#2 are not significantly affected by any of the applied stresses (S9B Fig and S9C Fig). Finally, the effects observed for I156Δ*H2A*.*Z*#3 (S9D Fig) resemble those observed in I349Δ*H2A*.*Z* (S2D Fig), with an increased sensitivity to NaCl and KCl but higher resistance to H_2_O_2_ stress.

**Fig 6:**
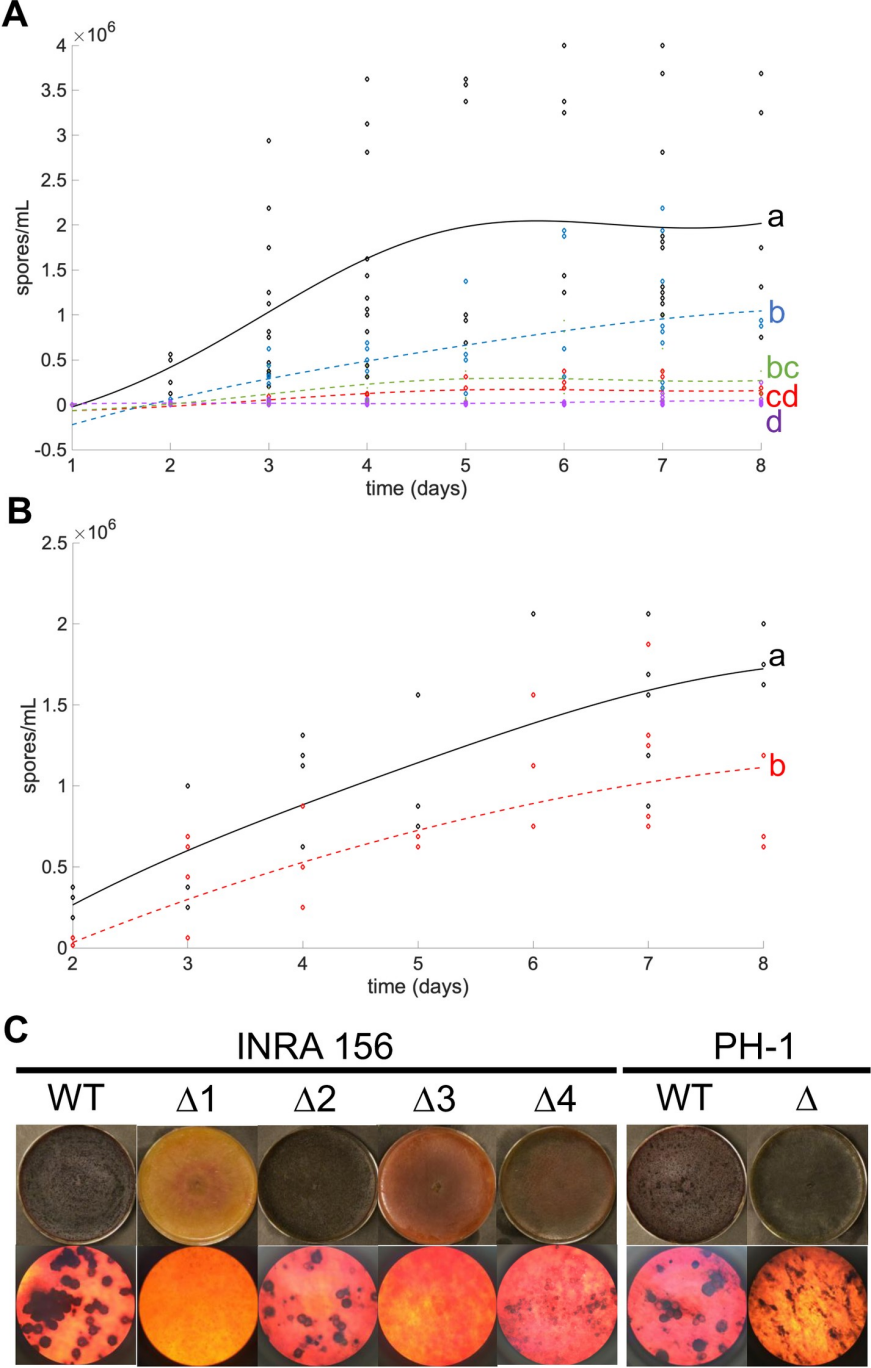
Asexual sporulation and formation of perithecia in INRA156 and PH-1 Δ*H2A*.*Z* mutants. **(A).** Fitted kinetics of sporulation for INRA156 wild-type and four Δ*H2A*.*Z* mutants. Black = wild-type; red = I156Δ*H2A*.*Z*#1; blue = I156Δ*H2A*.*Z*#2; purple = I156Δ*H2A*.*Z*#3; green = I156Δ*H2A*.*Z*#4. **(B).** Fitted kinetics of sporulation for PH-1 wild-type and PH-1Δ*H2A*.*Z* mutants. Black = wild-type; red = PH-1Δ*H2A*.*Z*. Letters indicate statistically significant curve groups after Kruskal-Wallis testing and Tukey-Kramer correction for multiple testing (*p* < 0.05). **(C).** Formation of perithecia on carrot agar by INRA156 and PH-1 wild-type and corresponding Δ*H2A*.*Z* mutants. Top lane: macroscopic view; bottom lane: microscopic view (x40). Pictures were taken four days after induction of sexual differentiation.

Differences between mutant strain behaviors were also observed regarding the ability to produce asexual spores. Similar to I349Δ*H2A*.*Z*, mutant strains in INRA156 and PH-1 backgrounds were affected in their ability to sporulate (**Fig 6A** and **6B**). The intensity of the defect was however moderate for two of them, I156Δ*H2A*.*Z*#2 (**Fig 6A**, blue curve) and PH-1Δ*H2A*.*Z* (**Fig 6B**, red curve). Since INRA156 and PH-1 show great capability to produce perithecia *in vitro*, we also investigated this property in their deletion mutants. The heterogeneity of the consequences of deleting *H2A*.*Z* is striking (**Fig 6C**): two mutants (I156Δ*H2A*.*Z*#1 and I156Δ*H2A*.*Z*#3) had their production of perithecia completely abolished; two mutants produced micro-perithecia (I156Δ*H2A*.*Z*#4 and PH-1Δ*H2A*.*Z*) or not viable ascospores (respectively); one mutant (I156Δ*H2A*.*Z*#2) had retained full capability to produce full-size perithecia (although somehow in lesser numbers) that could produce viable spores. Phenotype heterogeneity is also illustrated with the production of the mycotoxins DON and 15-ADON (**Fig 3B**). Nearly abolished in I156Δ*H2A*.*Z*#3, I156Δ*H2A*.*Z*#4, and PH-1Δ*H2A*.*Z* (similar to the situation in I349Δ*H2A*.*Z*), toxin production is heavily stimulated in I156Δ*H2A*.*Z*#1, and not significantly affected in I156Δ*H2A*.*Z*#2 (**Fig 3C**).

We investigated the occurrence of potential compensatory mutations in all Δ*H2A*.*Z* strains in both INRA156 and PH-1 background, similarly to the analysis we performed for INRA349 and its derived mutants. Putative compensatory mutations were detected in the two genes previously identified, namely FGRAMPH1_01G23597 and FGRAMPH1_01G18675, plus eight other genes in the five Δ*H2A*.*Z* mutants obtained in INRA156 and PH-1 backgrounds (Table 1). Strikingly, most genes are annotated as to encode proteins directly involved in chromatin remodeling in the reference functional database FungiDB [81,82].

**Table 1:** List of compensatory mutations detected.

Parental strain	Mutant	Gene ID*	Description*	Coding-region change	Amino Acid change	Ortholog* essential in *N*. crassa**?
INRA349	Δ*H2A*.*Z*	FGRAMPH1_01G23597	essential subunit of the histone deacetylase rpd3s complex	2015delA	Ile673fs	No
INRA349 ΔH2A.Z	Δ*H2A*.*Z*::*H2A*.*Z*	FGRAMPH1_01G23597	essential subunit of the histone deacetylase rpd3s complex	2015delA	Ile673fs	No
		FGRAMPH1_01G18675	helicase swr1	2555A>C	His852Pro	No
INRA156	Δ*H2A*.*Z*#1	FGRAMPH1_01G16577	oxidoreductase yusz	262G>T	Asp88Tyr	No
		FGRAMPH1_01G18925	histone demethylase jarid1	239_240insA	Val81fs	No
	Δ*H2A*.*Z*#2	FGRAMPH1_01G14931	Histone H3	49C>A	Pro17Thr	Probably essential
		FGRAMPH1_01G18675	helicase swr1	2545_2547delCTT	Leu850del	No
	Δ*H2A*.*Z*#3	FGRAMPH1_01G03975	hypothetical protein	82A>G;100G>A	Thr28Ala; Glu34Lys	n/a
		FGRAMPH1_01G11173	histone-lysine n-methyltransferase ash1l	1609C>T	His537Tyr	Probably essential
		FGRAMPH1_01G26173	Transcription factor	741_742insC	Ala248fs	No
		FGRAMPH1_01G26683	Hypothetical protein	406C>T	Pro136Ser	n/a
	Δ*H2A*.*Z*#4	FGRAMPH1_01G23597	essential subunit of the histone deacetylase rpd3s complex	3474_3475delTG	Val1159fs	No
PH-1	Δ*H2A*.*Z*	FGRAMPH1_01G03975	hypothetical protein	37G>A	Asp13Asn	n/a
		FGRAMPH1_01G27197	Hypothetical protein	1432C>T	Arg478*	No

* Source: FungiDB Release 46

**The Neurospora Genome Project [115]; n/a indicates no ortholog identified; identical genes with mutations in more than one genetic background are colored with the same shade of grey

In three instances, the same gene is affected in two Δ*H2A*.*Z* mutants from two different parental strains, albeit not with the exact same mutation. The first case regards I349Δ*H2A*.*Z* and I156Δ*H2A*.*Z*#4 that each carry a version of a frameshift mutation in the essential subunit of the histone deacetylase Rpd3S complex encoded by FGRAMPH1_01G23597. As evoked earlier, frameshifts creating non-sense mutations may result in the absence of the protein rather than the presence of a truncated one due to the activation of the NMD or the UPR pathways. In I156Δ*H2A*.*Z*#4, valine 1,159 becomes the site of a frameshift mutation. This mutation occurs towards the end of the protein and modifies profoundly its primary sequence with the likely consequence of losing the ability to undergo the conformational changes necessary to its proper activity [83]; it does not affect, at least directly, the functional domains of the protein as it can be the case in I349*ΔH2A*.*Z* (**Fig 4A**). Anecdotally, the latter is more severely affected in its growth and response to stress than I156*ΔH2A*.*Z*#4 (see S10 Fig for a visual summary), which suggest a more targeted rescue of *H2A*.*Z* deletion that does not (or not so much) compromises other H2A.Z-independent functions. Such differences in phenotypes between I349Δ*H2A*.*Z* and I156Δ*H2A*.*Z*#4 are remarkable since they both carry a severe mutation on the same gene, albeit not exactly the same one. While phenotypic differences may be attributed to the different genetic backgrounds, an alternative hypothesis is that both events affecting FGRAMPH1_01G23597 did not lead to null loss-of-function in both cases (or any), the alternatives being a leaky loss-of-function (with some functionality preserved) or a gain-of-function.

The second case of genes independently mutated regards I349Δ*H2A*.*Z*::*H2A*.*Z* and I156Δ*H2A*.*Z#2* that each harbor mutated *SWR1* (FGRAMPH1_01G18675), in addition to other mutations (Table 1). In I156Δ*H2A*.*Z*#2, leucine 850 is entirely removed, a mutated site located only two amino acids upstream the His852Pro found in I349Δ*H2A*.*Z*::*H2A*.*Z* and within the ATP-binding site of the DEAD box helicase-containing domain (**Fig 4B**). The activity of mutated FgSwr1, involved in the incorporation of H2A.Z in nucleosomes, is here very likely affected. Another mutation was found in the same mutant I156Δ*H2A*.*Z*#2: proline 17 (position 16 in the classical histone numbering since the initial methionine is cleaved off) histone H3 (FGRAMPH1_01G14931) is here replaced by a threonine. Proline isomerization influences protein secondary structure, and protein isomerases are found in many chromatin remodeling complexes [84]. Their exact role is however unclear. In histone H3, interplay between histone lysine methylation and proline isomerization has been demonstrated in *S*. *cerevisiae* [85]; for example, proline 38 was found to be necessary for H3K36 methylation. However, the potential function of H3P16 isomerization remains to be uncovered. As mentioned above, Swr1 is a major actor of the deposition of the dimer H2A.Z-H2B to replace a dimer H2A-H2B, notably *via* interactions with (H3-H4)_2_. Mutations in both Swr1 and H3 may prevent protein interaction and subsequent removal of H2A-H2B, thus stabilizing durably the histone octamer in the absence of H2A.Z and rescuing part of the deficiency. Among all Δ*H2A*.*Z* mutants in our set, I156Δ*H2A*.*Z*#2 certainly appears as the one in which the deleterious effects of deleting *H2A*.*Z* are best rescued.

The last case of genes repeatedly but independently mutated concerns I156Δ*H2A*.*Z*#3 and PH-1Δ*H2A*.*Z* that each harbor mutated FGRAMPH1_01G03975 (**Table 1**), encoding a small hypothetical protein with no known conserved domain. Considering that this gene is found immediately downstream and in the vicinity of FgH*2A*.*Z*, the mutations observed may be consequences of such proximity during knock-out assays. Nonetheless, other loci are also affected by secondary mutations in both I156Δ*H2A*.*Z*#3 and PH-1Δ*H2A*.*Z*. In the latter, the CDS of FGRAMPH1_01G27197 is interrupted at arginine 478 (out of 744 amino acids total) by a stop codon removing more than 35% of the protein (**Table 1**). Proline and alanine-rich (14% and 12%, respectively), its product is annotated as “hypothetical protein” (FungiDB v46) and no clear similarity to known features could shed any light on its possible function. In I156*ΔH2A*.*Z*#3, no less than three genes in addition to FGRAMPH1_01G03975 were affected by secondary mutations: FGRAMPH1_01G26683 encoding a hypothetical protein (Pro136Ser), FGRAMPH1_01G26173 encoding a transcription factor (Ala248fs), and FGRAMPH1_01G11173 encoding the histone-lysine methyltransferase Ash1l (S1 Table and S11 Fig). Although its function is unknown, a domain search in FGRAMPH1_01G26683 found a conserved Clr5 domain at the N-terminal end (S11A Fig); in fission yeast, Clr5 is involved in chromatin-mediated silencing and is in the same functional pathway as the histone deacetylase Clr6 (Rpd3). FGRAMPH1_01G26173 is a transcription factor containing a bZIP_ATF2 domain (S11B Fig) with a leucine zipper-type dimer interface followed by a proline-alanine-rich region of unknown function. The frameshift mutation occurs in the middle of this region. Finally, the histone methyltransferase Ash1l (SET2, FGRAMPH1_01G11173) contains a SET2_ASH1L domain (S11C Fig) that can bind H3K36 to add methyl groups using co-factor S-adenosyl methionine [86]. The mutation His537Tyr occurs within the active site, likely impeding the binding of S-adenosyl methionine [87]. Remarkably, among all obtained mutants, I156Δ*H2A*.*Z*#3 is the one most affected with secondary mutations (hitting four genes) and also the one with the most drastic phenotypes.

The last mutant, I156Δ*H2A*.*Z*#1, carries secondary mutations in two genes not affected in any of the other mutants: FGRAMPH1_01G16577 encoding the oxidoreductase Yusz, and FGRAMPH1_01G18925 encoding the histone demethylase Jarid1 (Table 1, and S11D Fig and S9E Fig). FgYusz is a short-chain dehydrogenase/reductase (SDR) with a structurally conserved Rossmann fold (alpha/beta folding pattern with a central beta-sheet; [88–91]). Its function in *F*. *graminearum* is currently unknown. The mutation Asp88Tyr is located within the NADP-binding site, thus with potential consequences in the activity of the protein (S11D Fig). Fg*Yusz* is found highly expressed in conidia, perithecia, and during wheat infection but not in vegetative hyphae [56,58,92]. Our observations of asexual sporulation and perithecia formation being affected whereas radial growth is less severely impaired supports a role in conidiation and sexual reproduction. Regarding the histone demethylase Jarid1 (Kdm5), involved in demethylating H3K4 [93], a single nucleotide insertion has dramatic consequences. A frameshift at the N-terminal end of the protein truncates the protein down to 114 aa with a completely modified sequence from position 81 to 114 (S11E Fig), completely removing all functional domains and binding sites necessary for proper function [94]. Incidentally, I156Δ*H2A*.*Z*#1 seems to cope better with stress during vegetative growth, including H_2_O_2_ 15 mM, than the wild type parent and produces much higher levels of toxins (**Figs 3** and **5**). This observation is consistent with DON and 15-ADON production being part of *F*. *graminearum* stress response [66]. FgKdm5 has been recently characterized in *F*. *graminearum* and was shown to be a positive regulator of secondary metabolism, in a demethylation-independent manner for four out of five secondary metabolites tested including DON [50]. Similarly, in *Aspergillus nidulans*, a histone demethylation-independent role of KdmB (FgKdm5) in activating some but not all genes involved in the production of secondary metabolites has been proposed [95]. In the present study, losing the canonical FgKdm5 did not prevent the activation of toxin production, suggesting the detected mutation did not cause of null loss-of-function, or the intervention of another Kdm5-independent pathway stimulated toxin production. In *F*. *fujikuroi* the homolog FfKdm5 balances H3K4me3 levels and affects secondary metabolism, hyphal growth and sporulation [96].

As a whole, our observations may suggest that deleting *H2A*.*Z* from the genome of *F*. *graminearum* causes particularly deleterious effects that would challenge viability in the absence of compensatory mutations.

### H2A.Z appears essential also in another *Fusarium* spp.

To explore the possible essentiality of H2A.Z in other *Fusarium* spp., attempts to delete *H2A*.*Z* were performed in the distantly related rice pathogen *Fusarium fujikuroi* [97]. *F*. *fujikuroi* is a rice pathogen and well known for the production of gibberellins, phytohormones that are produced during the infection and the causative agent for the typical symptoms of the *bakanae* disease, *i*.*e*. chlorotic, slender and etiolated rice stalks [98]. H2A.Z was identified in the *F*. *fujikuroi* wild-type strains IMI58289 (FFUJ_01849), B14 (FFB14_11687) and E282 (FFE2_11549) by determining FgH2A.Z orthologs using QuartetS [99], and here referred to as FfH2A.Z. To analyze the function of FfH2A.Z, a gene replacement cassette (pΔ*ffH2A*.*Z*) was generated and used for targeted deletion of *FfH2A*.*Z* in all three wild-type backgrounds (S12A Fig). In total, 15 hygromycin-resistant transformants were gained for IMI58289, all heterokaryons of nuclei showing homologous integration events mixed with wild-type ones. To obtain homokaryotic mutants three rounds of repeated single spore isolations were performed for all of them. However, attempts to generate hygromycin-resistant homokaryotic mutants failed as not one single mutant obtained during the process showed absence of the *FfH2A*.*Z* wild-type gene (S12B Fig**)**. Similarly, attempts to delete *H2A*.*Z* in B14 and E282 resulted in several hygromycin-resistant transformants showing homologous integration events. For three independent transformants each, attempts to generate homokaryotic mutants by single spore isolation were performed. Again, no homokaryotic mutant showing absence of *FfH2A*.*Z* was obtained during three repeated rounds of single spore isolation (S12B Fig). Next, we tried to generate homokaryotic mutants by performing protoplast isolation in all three different wild-type backgrounds. Again, not a single hygromycin-resistant homokaryotic strain deleted for *FfH2A*.*Z* was obtained (S12B Fig), suggesting that removal of H2A.Z is also lethal in *F*. *fujikuroi*.

## Conclusion

Histone variant H2A.Z is found ubiquitously in fungi, plant and animal species. In the present study, we conducted attempts to knock out *H2A*.*Z* from the *F*. *fujikuroi* and *F*. *graminearum* genome in three different strains each. While not one single homokaryotic deletion mutant was recovered in all three *F*. *fujikuroi* backgrounds, a total of six homokaryotic *H2A*.*Z* deletion mutants were obtained in the case of *F*. *graminearum*. Notably, all six exhibited heterogenous phenotypes. Moreover, adding-back *H2A*.*Z* in I349Δ*H2A*.*Z* did not restore wild-type phenotype. We found that each one of *F*. *graminearum* genetically modified strain we engineered contained one or more secondary mutations elsewhere in the genome, not present in the wild-type parental strain. Thus, we propose that H2A.Z is essential in both *F*. *fujikuroi* and *F*. *graminearum*. In the latter, the occurrence of compensatory mutations rescues lethality.

In our study, the six *H2A*.*Z* deletion mutants obtained in three different *F*. *graminearum* genetic backgrounds harbor different mutations, some of them in the same genes. The fact that independent repetitions of knock-out experiments often lead to secondary mutations in the same gene has been previously observed in yeast, a phenomenon referred to as parallel evolution [100]. The numerous failed attempts we faced to obtain Δ*H2A*.*Z* mutants suggest that there are only a few suitable mutations that could be selected. On the evolutionary scale, the number of combinations that are sufficiently fit to persist may be limited, in line with the proposition from Davis and Colleagues (2007). Such mutations, indeed, usually occur for proteins that are in the same functional module [101]. In *F*. *graminearum*, an example of the suppression of growth defects provoked by the deletion of the kinase FgPrp4, involved in the spliceosome, has been evidenced and involves spontaneous mutation in *FgSAD1* involved in pre-mRNA complex assembly [102]. In our study, all mutants were affected in at least one known protein involved in chromatin remodeling, with the exception of PH-1Δ*H2A*.*Z* for which no function could be proposed for any of the protein containing secondary mutations. Altogether, the critical relationship between H3K36me and H2A.Z is largely highlighted here. The flexible changes of the regulatory networks around these marks reveal the power of network re-wiring to maintain a critical level of transcriptional balance. Some of the genetic innovations that arose from our experiments seem to give better results than others in the light of the traits tested. The overall fitness of our mutant strains to survive and thrive under actual conditions remains to be explored.

The mark H3K4me2/3 has also been underlined as in the same functional network as H2A.Z in our mutant I156Δ*H2A*.*Z*#1 defective in the histone demethylase Jarid1 (FgKdm5), which produces much higher levels of toxin than the wild-type. This observation is consistent with findings in *F*. *fujikuroi* in which methylated H3K4 is deposited by SET1 and positively regulates, among other traits, secondary metabolism [96]. In this mutant, active transcription may be promoted by maintaining methylated H3K4.

As a whole, in the present study, the observed phenotypes are the result of interactions between two or more mutations, under the influence of the genetic background. One feature emerges, lack of H2A.Z is likely lethal in both fusaria investigated here, *i*.*e*., *F*. *fujikuroi* and *F*. *graminearum*. In the latter, however, its extraordinary plasticity allows compensation within the short time frame of a lab transformation. Our study highlights peculiar links between H2A.Z and H3K36/H3K4, advancing the search for H2A.Z functional network.

## Materials and methods

### Fungal strains and culture media

Wild-type *F*. *graminearum* strains INRA156 (INRA-MycSA collection), INRA349 (CBS185.32; CBS-KNAW Collection, the Netherlands), INRA812 (PH-1 strain FGSC9075; Fungal Genetics Stock Center, the USA) were propagated on Potato Dextrose Agar (PDA; DIFCO, France) plates at 25°C. INRA156 can efficiently undergo sexual reproduction but produces only moderate to low levels of DON and 15-ADON, whereas INRA349 always fails to produce perithecia but consistently produces very high levels of DON and 15-ADON; INRA812 is the sequenced reference strain widely used in *Fusarium* related studies (can reproduce sexually and produces only very low levels of DON and 15-ADON). Radial growth assays were performed on complete medium [103] supplemented prior solidification with NaCl, KCl, H_2_O_2_, or not supplemented. When needed, conidia were prepared by inoculating agar plugs in CMC medium (15 g/L carboxylmethyl cellulose, 1 g/L yeast extract, 0.5 g/L MgSO_4_.7H_2_O, 1 g/L NH_4_NO_3_, 1 g/L KH_2_PO_4_) [104], incubating at 150 rpm and 25°C for three to five days, and harvesting by filtration through Sefar Nitex 03–100 (100 μm, SEFAR AG—Switzerland). Toxin production was measured in liquid synthetic medium [105]. For spore germination assays, glucose in MS medium was replaced by sucrose.

Wild-type strains of *F*. *fujikuroi* IMI58289 (Commonwealth Mycological Institute, Kew, UK), *F*. *fujikuroi* B14 (S.-H. Yun, Korea), *F*. *fujikuroi* E282 (S. Tonti, University Bologna, Italy) were used as parental strains for deletion experiments. For protoplasting, relevant strains were grown for three days in 100 mL Darken medium [106] at 30°C and 180 rpm in the dark. A 500 μL aliquot was then used for inoculation of 100 mL liquid synthetic ICI medium [107] containing 10 g/L fructose and 1g/L NH_4_SO_4_ as sole carbon and nitrogen sources, respectively. For verification of Δ*H2A*.*Z* mutants and DNA isolation, strains were grown for three days on solid complete medium (CM) [108] covered with cellophane sheets (Folia Bringmann) at 30°C in darkness. Where applicable, plates were supplemented with 100 ppm hygromycin B. For conidia production, *F*. *fujikuroi* strains were grown for seven days on solid V8 medium (20% v/v vegetable juice, Campbell Food, Puurs, Belgium) at 20°C and 16/8 light-dark cycles. Cultivation of *S*. *cerevisiae* was performed as previously described [109].

### Identification of *FgH2A*.*Z*

We used a Hidden Markov Model-based approach [110] to identify proteins carrying a domain characteristic of the C-terminal end of histone H2A (PFAM accession number PF16211) in the reference proteome of *F*. *graminearum* PH-1 [78–80] available at FungiDB [81,82]. Putative identification was then assigned by sequence similarity with the reference protein database (ref_seq_protein_v5) at NCBI using BLASTP [55,111]. Genes structures and sequences, protein sequences, and transcript levels were retrieved from FungiDB [81,82]. Protein alignment was performed and displayed with CLC Workbench 10.0.

### Generation of gene deletion, add-back, and over-expression mutants

Wild-type *F*. *graminearum* strains INRA156, INRA349, and PH-1 were used as parental strains to engineer H2A.Z deletion mutants. Primer sequences and amplification conditions used are provided in S1 Table. Deletion strategy using the split-marker approach [112] is represented S2 Fig. Upstream and downstream flanking regions of *FgH2A*.*Z* (gene ID FGRAMPH1_01G03973) were amplified by PCR (primer pairs 3r-3UTR-H2AZ-R / 3UTR-H2AZ- HY-F, and 5UTR-H2AZ-GRO-R / 5f-UTR-H2AZ-F, respectively) from wild-type INRA349 genomic DNA. The hygromycin-resistance cassette hygroR, containing *hph* gene under the control of *N*. *crassa* pCPC1 and *A*. *nidulans* t*TrpC*, was amplified by PCR using primer pair neoHY-8-finF / neoHY-1-debutR and pBSK(-) NeoHygroR plasmid as template according to a previously described procedure [113]. Fragment assembly was achieved by multiple recombination of overlapping sequences in *S*. *cerevisiae* strain FY1679 (genotype MATa/MATα, ura3-52/ura3-52, trp1Δ63/TRP1, leu2Δ/LEU2, his3Δ200/HIS3, GAL2/GAL2) transformed with the three PCR products and the pRS426 plasmid digested with BamHI and HindIII [114]. SC-Ura medium containing 0.67% yeast nitrogen base without amino acids was used for selection. A second round of PCR was carried out using successfully transformed FY1679 DNA as template to amplify two overlapping fragments (primer pairs 3UTR-H2AZ-N-R / NP_SplitHY_R, and NP_SplitGRO_F / 5UTR-H2AZ-N-F) to be used for transformation into *F*. *graminearum* for the targeted replacement of H2A.Z with the hygromycin resistance cassette (hygroR). PCR products were purified and transformed into protoplasts of INRA156, INRA349, and PH-1, prepared according to a previously published protocol [113]. For add-back experiment, H2A.Z CDS fused to *tTrpC* terminator and followed by a geneticin-resistance cassette, genetR as previously described [113], (assembled in yeast similarly to the deletion experiments) was used for targeted replacement of the integrated hygroR cassette in INRA349*ΔH2A*.*Z* (S3A Fig and S1 Table). For over-expression experiments, wild-type H2A.Z CDS in INRA349 was replaced by *FgH2A*.*Z* CDS fused to *A*. *nidulans* promotor *pGPD* and preceded by the above described HygroR cassette (S3B Fig and S1 Table). All selected transformants were purified by two rounds of monoconidial isolation.

The plasmid for *H2A*.*Z* deletion in *F*. *fujikuroi* (IMI58289, B14 and E282) was generated using yeast recombinational cloning [109,115]. All primers used for polymerase chain reaction (PCR) were obtained from Sigma-Aldrich GmbH. Deletion strategy and respective primers for *H2A*.*Z* deletion in the different wild-type strains are displayed in S12 Fig as well as listed in S2 Table. Briefly, the upstream (5’) and downstream (3’) sequences of *FfH2A*.*Z* were amplified from *F*. *fujikuroi* IMI58289 wild-type genomic DNA using the following primer pairs: FfH2A.Z_5F // FfH2A.Z_5R for upstream, FfH2A.Z_3F // FfH2A.Z_3R for downstream regions. For the deletion construct, hygromycin B was used as a selection marker. The *hph* resistance cassette was amplified from pCSN44 [116] with the primer pair Hph_F // Hph_R. Transformation of *F*. *fujikuroi* (IMI58289, B14 and E282) was performed as previously described [97,117]. For this, the deletion fragments were amplified from pΔ*ffH2A*.*Z* with the primer pairs FfH2A.Z_5F // Ff_H2A.Z_3R, using a proof-reading polymerase (Q5-polymerase, New England Biolabs). Transformed protoplasts were regenerated as described [118]. Regeneration media contained 100 ppm hygromycin B. Homologous recombination events were verified with the primer pairs dia_FfH2A.Z_F // pCSN44_trpC_T for the upstream part and dia_FfH2A.Z_R // pCSN44_trpC_P2 for the downstream part (S12 Fig). Presence of the native wild-type gene was checked with the primers FfH2A.Z_WT_diaF // FfH2A.Z_WT_diaR.

### Radial growth assays

A total of 100 spores or a plug (3 mm in diameter, taken from the periphery of a seven-day-old CM culture plate) were cultured at 25°C in darkness on 94*16 mm plates containing CM, or CM with 1M NaCl / KCl, or CM with 5 mM / 15 mM H_2_O_2_. Experiments were done in triplicate. Growth area of individual Petri dishes was analyzed by ImageJ 1.8.0 image processing program [119].

### Conidiation and germination rate assays

For conidiation assays, one mycelial plug (8 mm in diameter) of each strain, taken from the periphery of a 7-day old colony on PDA plates was inserted in a 50 mL-filter screw cap conical tube containing 10 mL of CMC medium, and incubated for up to eight days at 25°C and 180 rpm in darkness in an Infors Multitron (INFORS-HT). Spores were counted in a Thoma cell counting chamber under microscope every 24 hours (three independent counts per sample). For germination assays, 10^6^ five-day old spores were incubated in 10 mL MS sucrose liquid medium at 25°C in darkness for up to eight hours. Germination rates were counted under microscope every hour, according to a previously published procedure [63]. All experiments were performed with three independent biological replicates.

### Sexual reproduction assays

Self-fertilization were performed on carrot agar plates as previously described [120]. Perithecia were observed under microscope two days after sexual induction. Ascospores were harvested by placing the carrot agar plates upside down and allowing the mature perithecia to discharge ascospores on petri dish covers. Ascospores were collected by adding 1 mL of sterile MilliQ water and observed under microscope. A volume of 10 μL of each suspension was inoculated in liquid CM to check whether ascospores could grow or not. Experiments were done in triplicate.

### Type B trichothecene analysis

Eight milliliters of MS liquid medium [105] were inoculated with 8x10^4^ conidia prepared in CMC medium and incubated at 25°C in the dark for up to 20 days. TCTB were extracted and analyzed by HPLC-DAD according to a previously published protocol [113].

### Whole genome sequencing and data analysis

Genomic DNA was extracted from 50 mg of freeze-dried mycelium, from all wild-type and mutant strains, as previously described [121]. Libraries were prepared from 500 ng of gDNA using the Westburg NGS DNA Library Prep Kit for Illumina (cat. # WB9024, Westburg, The Netherlands) following manufacturer’s instructions. Sequencing in paired mode, 2x100 bp, was performed by the GenomEast platform, a member of the ‘France Génomique’ consortium (ANR-10-INBS-0009) on an Illumina HiSeq 4000 (reads were deposited at SRA under the BioProject accession number PRJNA580372). Raw reads were pre-processed with Trimmomatic v0.39 [122]. The software CLC Genomics Workbench 10 (QIAGEN Bioinformatics, Denmark) was used for all subsequent bioinformatic analyses. Briefly, high-quality read pairs were mapped (including a re-alignment step) onto *F*. *graminearum* PH-1 reference genome. Coverage analysis was performed to confirm the correct insertion of the deletion/complementation cassette in all mutants. Variants were called (ploidy = 1 and variant probability = 0.9), and those found in wild-type were subtracted from their corresponding mutants. Each remaining variant was manually curated to ensure no false positive was retained. Fragments including compensatory mutations identified by whole genome sequencing of *ΔH2A*.*Z*, *ΔH2A*.*Z*::*H2AZ* and their corresponding wild-type strains were amplified by PCR. Products were sent to GENEWIZ for Sanger sequencing. Fragments were sequenced by two primers from the direction of forward and reverse, respectively (S1 Table). The software CLC Genomics Workbench 10 (QIAGEN Bioinformatics, Denmark) was used for sequence alignment and trace examination.

### Statistical analyses

For growth, sporulation, and germination assays, profiles were obtained by curve fitting through all points and differences statistically analyzed by Kruskal-Wallis test combined with Tukey-Kramer correction for multiple testing using Matlab R2019a (Mathworks). The statistical threshold was set at *p* = 0.05 throughout.

## Supporting information

S1 Text Additional materials and methods, and additional references. (DOCX)

S1 File BLASTP report for query translated protein FGRAMPH1_01T26109. (PDF)

S2 File BLASTP report for query translated protein FGRAMPH1_01T03973. (PDF)

S1 Fig Schematic representations of *F. graminearum* H2A (FGRAMPH1_01G26109) *vs.* H2AZ (FGRAMPH1_01G03973) gene and protein sequences. (A) Genomic sequences. Blue boxes are exons (e); green boxes are introns; black lines are UTRs. Translation start (ATG) and stop sites are indicated with orange and yellow arrows, respectively. Scaling key is provided in grey. (B). *FgH2A* and *FgH2A*.*Z* expression levels (in FPKM) previously measured by Zhao and Colleagues [1] in asexual spores (black bars) or actively growing mycelium (white bars). Displayed values are means of three replicates and error bars are standard deviations. (C). Protein alignment of FgH2A and FgH2A.Z. Residues were colored according to Rasmol scheme. Red boxes indicate domains essential for H2A.Z function (*vs*. H2A) according to Suto et al. [2]. All datasets can be viewed at FungiDB [3,4] (see ‘Additional References’ in S1 Text).
(TIFF)

S2 Fig Split-marker approach used for the deletion of H2A.Z in *F. graminearum*. **(A)** The upstream and downstream flanking regions of *H2A*.*Z*, *HygroR* cassette were amplified from wild type *F*. *graminearum* genomic DNA and plasmid DNA of pBlueScriptSK(-)_NeoHygroR, respectively. **(B)** Fragment assembly in yeast. The three fragments were assembled by plasmid pRS426. **(C)** Extraction of yeast genomic DNA and amplification of fragments for split-marker method in *F*. *graminearum*. **(D)** Transformation in *F*. *graminearum*. *H2A*.*Z* was replaced by *HygroR* cassette.
(TIFF)

S3 Fig I349 deletion mutant screening by PCR and Southern-Blot (see ‘Additional Materials and Methods’ in S1 Text). **(A)** Schematic representation of the *loci* amplified. Primers matching the upstream and downstream flanking regions of *H2A*.*Z* were used to amplify a 1,588 bp- or 2,717 bp-long fragments from wild-type INRA349 gDNA or Δ*H2A*.*Z* mutant gDNA, respectively. **(B)** Result of the agarose gel electrophoresis of the fragments obtained from S3A Fig. Lane 1: ladder; lane 2: INRA349 WT; lane 3: I349Δ*H2A*.*Z*. **(C)** Schematic representation of the *Southern blot* strategy used. Digestion with *NdeI* of gDNA extracted from I349 wild-type or ΔH2A.Z leads to fragments of 7,687 bp and 3,847 bp in size, respectively. A probe marked with digoxygenin and matching the upstream region of H2A.Z CDS as well as part of its 5’ end was then synthesized and used to reveal the two digestion fragments obtained on X-ray films (as displayed in **(D)**; Lane 1: INRA349 WT; lane 2: I349ΔH2A.Z. WT = INRA349 WT; Δ = I349ΔH2A.Z.
(TIFF)

S4 Fig Schematic representation of the split-marker strategy used to add back *H2A*.*Z* to INRA349*ΔH2A*.*Z* at its original locus **(A)**, or place it under the control of the strong constitutive promoter pGPD **(B)**. **(C)** Rq expression levels for H2A.Z in INRA349OE:H2A.Z (grey bar; Rq = 7.6) and INRA349*ΔH2A*.*Z* (stripped bar; Rq = 0.978) relative to wild type (black bar; Rq = 1), measured by RT-qPCR. Displayed values are means of three replicates, error bars are standard deviations. The star indicates significant difference with *p* < 0.01.
(TIFF)

S5 Fig Effect of abiotic stress on radial growth. **(A)** 3 dpi and **(B)** 6 dpi radial growth in I349Δ*H2A*.*Z* (light grey), I349Δ*H2A*.*Z*::*H2A*.*Z* (stripes), and I349OE::*H2A*.*Z* (dark grey) expressed relative to growth of wild-type in not supplemented CM medium (black solid line marking 100% of growth). **(C)** and **(D)** Fitted radial growth kinetics followed for six days for I349 OE:*H2A*.*Z* **(C)** and I349Δ*H2A*.*Z*::*H2A*.*Z* **(D)**. Black = CM; red = CM + NaCl 1M; blue = CM + KCl 1M; purple = CM + H_2_O_2_ 5 mM; green = CM + H_2_O_2_ 15 mM. In all panels, letters indicate statistically significant curve groups after Kruskal-Wallis testing and Tukey-Kramer correction for multiple testing (*p* < 0.05).
(TIFF)

S6 Fig Visualization of deep sequencing results at H2A.Z for INRA349 wild-type, its deletion mutant I349Δ*H2A.Z*, and the add-back I349Δ*H2A.Z*::*H2A.Z*. **(A)** JBrowse screenshot of read coverages per base. **(B)** and **(C)** Mutations detected elsewhere in the genomes of I349Δ:*H2A*.*Z* **(B)** and I349Δ*H2A*.*Z*::*H2A*.*Z* **(C)**.
(TIFF)

S7 Fig Snapshot of Sanger sequencing results of *F. graminearum* wild-type and mutant strains. Nucleotides in black are nucleotides that are different.
(TIFF)

S8 Fig Consequences of the Ile673fs mutation on the primary sequence of the protein encoded by FGRAMPH1_01G23597 in I349ΔH2A.Z. Protein sequence alignment of wild-type (WT) and mutated (ΔH2A.Z) FGRAMPH1_01G23591. Only the C-terminal end of the protein, containing the mutation is depicted. Pink boxes highlight differences between sequences.
(TIFF)

S9 Fig Fitted radial growth kinetics followed for six days for INRA156 wild-type **(A)** and its four ΔH2A.Z mutants **(B)** to **(E)**, and PH-1 wild-type **(F)** and its Δ*H2A*.*Z* mutant **(G).** Black = CM; red = CM + NaCl 1M; blue = CM + KCl 1M; purple = CM + H_2_O_2_ 5 mM; green = CM + H_2_O_2_ 15 mM. Letters indicate statistically significant curve groups after Kruskal-Wallis testing and Tukey-Kramer correction for multiple testing (*p* < 0.05).
(TIFF)

S10 Fig Visual comparison of developmental defects in the deletion mutants I349ΔH2A.Z and I156ΔH2A.Z#4 that both carry a frameshift mutation in FGRAMPH1_01G23597. **(A)** Fitted radial growth kinetics followed for six days. **(B)** Fitted kinetics of sporulation. **(A)** and **(B)** Solid black = wild-type I349; dashed black = wild-type I156; solid red = I349Δ*H2A*.*Z*; dashed red = I156Δ*H2A*.*Z*#4. **(C)** Effect of NaCl (red bars), KCl (blue bars), H_2_O_2_ 5 mM (purple bars), and H_2_O_2_ 15 mM (green bars) after 6 day-long radial growth of INRA349 wild-type (I349WT), INRA156 wild-type (I156WT), and their deletion mutants I349ΔH2A.Z (I349Δ) and I156ΔH2A.Z#4 (I156Δ4). For comparison sake, growth of a given strain is expressed in percentage relative to itself grown in not supplemented CM medium (black bars).
(TIFF)

S11 Fig Domain architecture of proteins affected by secondary mutations in INRA156 and PH-1 Δ*H2A.Z* mutants. **(A)** Protein of unknown function (FGRAMPH1_01G26683; 537 aa) containing a Clr5 domain (PFAM14420). “Pro136Ser” indicates a mutation. **(B)** Transcription factor (FGRAMPH1_01G26173; 297 aa) containing a bZIP_ATF2 domain (CL21462). “Ala248fs” indicates a mutation. **(C)** SET2 protein (FGRAMPH1_01G11173; 786 aa) containing a SET_ASH1L domain (CL1917) and an AWS domain (PFAM17907). “His537Tyr” indicates a mutation. **(D)** Yusz oxidoreductase (FGRAMPH1_01G16577; 292 aa) containing a 17beta-HSD-like_SDR_c domain (CD05374). “Asp88Tyr” indicates a mutation. **(E)** Jarid1 histone demethylase (FGRAMPH1_01G18925; 1,731 aa) containing various domains (PLU-1 PFAM08429, JmjC PFAM02373, ARID SMART01014, PHD_Ecm5p_Lid2p_like CD15518, JmjN SMART00545, PHD_SF super family CL22851, zf-C5HC2 PFAM02928). “Val81fs” indicates a mutation. Architectures are displayed with SnapGene Viewer 5.0.6.
(TIFF)

S12 Fig Summary of *H2A*.Z deletion attempts in *F. fujikuroi*. **(A)** Schematic representation of the deletion strategy and position of the primers used for PCR validation **(B)**. Visualization of typical PCR results after three rounds of single-spore isolation. + = Δ*ffH2A*.*Z* gDNA before single spore isolation; FfWT = *F*. *fujikuroi* wild-type gDNA. Results are shown for three transformants per strain tested **(C)** Visualization of PCR results after protoplast isolation. + = Δ*ffH2A*.*Z* gDNA before protoplast isolation.
(TIFF)

S1 Table Primers used in the *F. graminearum* study. (XLSX)

S2 Table Primers used in the *F. fujikuroi* study. (XLSX)

## References

[1] Balzer A, Tardieu D, Bailly J, Guerre P. The trichothecenes: Toxins nature, natural occurrence in food and feeds, and ways of struggle. Rev Med Veterinaire. 2004 6 1;155:299–314.

[2] Goswami RS, Kistler HC. Heading for disaster: Fusarium graminearum on cereal crops. Mol Plant Pathol. 2004 11 1;5(6):515–25. 10.1111/j.1364-3703.2004.00252.x 20565626

[3] Sutton JC. Epidemiology of wheat head blight and maize ear rot caused by Fusarium graminearum. Can J Plant Pathol. 1982 6 1;4(2):195–209.

[4] McMullen M, Jones R, Gallenberg D. Scab of Wheat and Barley: A Re-emerging Disease of Devastating Impact. Plant Dis. 1997 12 1;81(12):1340–8. 10.1094/PDIS.1997.81.12.1340 30861784

[5] Desjardins AE, Hohn TM, McCormick SP. Trichothecene biosynthesis in Fusarium species: chemistry, genetics, and significance. Microbiol Rev. 1993;57(3):595–604. 8246841 10.1128/mr.57.3.595-604.1993 PMC372927

[6] Kimura M, Tokai T, Takahashi-Ando N, Ohsato S, Fujimura M. Molecular and genetic studies of fusarium trichothecene biosynthesis: pathways, genes, and evolution. Biosci Biotechnol Biochem. 2007 9;71(9):2105–23. 10.1271/bbb.70183 17827683

[7] Figueroa M, Hammond-Kosack KE, Solomon PS. A review of wheat diseases—a field perspective. Mol Plant Pathol. 2018;19(6):1523–36. 10.1111/mpp.12618 29045052 PMC6638159

[8] Richmond TJ, Finch JT, Rushton B, Rhodes D, Klug A. Structure of the nucleosome core particle at 7 Å resolution. Nature. 1984 10 11;311:532 10.1038/311532a0 6482966

[9] Luger K, Dechassa ML, Tremethick DJ. New insights into nucleosome and chromatin structure: an ordered state or a disordered affair? Nat Rev Mol Cell Biol. 2012 6 22;13:436 10.1038/nrm3382 22722606 PMC3408961

[10] Audia JE, Campbell RM. Histone Modifications and Cancer. Cold Spring Harb Perspect Biol [Internet]. 2016 4 [cited 2019 Aug 12];8(4). Available from: https://www.ncbi.nlm.nih.gov/pmc/articles/PMC4817802/ 10.1101/cshperspect.a019521 27037415 PMC4817802

[11] Malik HS, Henikoff S. Phylogenomics of the nucleosome. Nat Struct Mol Biol. 2003 11;10(11):882–91. 10.1038/nsb996 14583738

[12] Bonisch C, Hake SB. Histone H2A variants in nucleosomes and chromatin: more or less stable? Nucleic Acids Res. 2012 11 1;40(21):10719–41. 10.1093/nar/gks865 23002134 PMC3510494

[13] Bernstein E, Hake SB. The nucleosome: a little variation goes a long wayThis paper is one of a selection of papers published in this Special Issue, entitled 27th International West Coast Chromatin and Chromosome Conference, and has undergone the Journal’s usual peer review process. Biochem Cell Biol. 2006 8;84(4):505–7. 10.1139/o06-085 16936823

[14] van Daal A, White EM, Elgin SC, Gorovsky MA. Conservation of intron position indicates separation of major and variant H2As is an early event in the evolution of eukaryotes. J Mol Evol. 1990 5;30(5):449–55. 10.1007/BF02101116 2111857

[15] Thatcher TH, Gorovsky MA. Phylogenetic analysis of the core histones H2A, H2B, H3, and H4. Nucleic Acids Res. 1994 1 25;22(2):174–9. 10.1093/nar/22.2.174 8121801 PMC307768

[16] Luger K, Mäder AW, Richmond RK, Sargent DF, Richmond TJ. Crystal structure of the nucleosome core particle at 2.8 Å resolution. Nature. 1997 9;389(6648):251–60. 10.1038/38444 9305837

[17] Suto RK, Clarkson MJ, Tremethick DJ, Luger K. Crystal structure of a nucleosome core particle containing the variant histone H2A.Z. Nat Struct Biol. 2000 12;7(12):1121–4. 10.1038/81971 11101893

[18] March-Díaz R, Reyes JC. The beauty of being a variant: H2A.Z and the SWR1 complex in plants. Mol Plant. 2009 7;2(4):565–77. 10.1093/mp/ssp019 19825639

[19] Jackson JD, Falciano VT, Gorovsky MA. A likely histone H2A.F/Z variant in Saccharomyces cerevisiae. Trends Biochem Sci. 1996 12;21(12):466–7. 10.1016/s0968-0004(96)20028-3 9009827

[20] van Daal A, Elgin SC. A histone variant, H2AvD, is essential in Drosophila melanogaster. Mol Biol Cell. 1992 6;3(6):593–602. 10.1091/mbc.3.6.593 1498368 PMC275615

[21] Hatch CL, Bonner WM. The human histone H2A.Z gene. Sequence and regulation. J Biol Chem. 1990 9 5;265(25):15211–8. 1697587

[22] Downs JA, Allard S, Jobin-Robitaille O, Javaheri A, Auger A, Bouchard N, et al Binding of chromatin-modifying activities to phosphorylated histone H2A at DNA damage sites. Mol Cell. 2004 12 22;16(6):979–90. 10.1016/j.molcel.2004.12.003 15610740

[23] Keogh M-C, Kim J-A, Downey M, Fillingham J, Chowdhury D, Harrison JC, et al A phosphatase complex that dephosphorylates gammaH2AX regulates DNA damage checkpoint recovery. Nature. 2006 1 26;439(7075):497–501. 10.1038/nature04384 16299494

[24] Krogan NJ, Peng W-T, Cagney G, Robinson MD, Haw R, Zhong G, et al High-definition macromolecular composition of yeast RNA-processing complexes. Mol Cell. 2004 1 30;13(2):225–39. 10.1016/s1097-2765(04)00003-6 14759368

[25] Kalocsay M, Hiller NJ, Jentsch S. Chromosome-wide Rad51 spreading and SUMO-H2A.Z-dependent chromosome fixation in response to a persistent DNA double-strand break. Mol Cell. 2009 2 13;33(3):335–43. 10.1016/j.molcel.2009.01.016 19217407

[26] Xu Y, Ayrapetov MK, Xu C, Gursoy-Yuzugullu O, Hu Y, Price BD. Histone H2A.Z Controls a Critical Chromatin Remodeling Step Required for DNA Double-Strand Break Repair. Mol Cell. 2012 12 14;48(5):723–33. 10.1016/j.molcel.2012.09.026 23122415 PMC3525728

[27] Gursoy-Yuzugullu O, Ayrapetov MK, Price BD. Histone chaperone Anp32e removes H2A.Z from DNA double-strand breaks and promotes nucleosome reorganization and DNA repair. Proc Natl Acad Sci. 2015 6 16;112(24):7507–12. 10.1073/pnas.1504868112 26034280 PMC4475971

[28] Alatwi HE, Downs JA. Removal of H2A.Z by INO80 promotes homologous recombination. EMBO Rep. 2015 8 1;16(8):986–94. 10.15252/embr.201540330 26142279 PMC4552491

[29] Marques JT, Kim K, Wu P-H, Alleyne TM, Jafari N, Carthew RW. Loqs and R2D2 act sequentially in the siRNA pathway in Drosophila. Nat Struct Mol Biol. 2010 1;17(1):24–30. 10.1038/nsmb.1735 20037596 PMC2919300

[30] Deal RB, Henikoff S. The INTACT method for cell type-specific gene expression and chromatin profiling in Arabidopsis thaliana. Nat Protoc. 2011 1;6(1):56–68. 10.1038/nprot.2010.175 21212783 PMC7219316

[31] Soboleva TA, Nekrasov M, Ryan DP, Tremethick DJ. Histone variants at the transcription start-site. Trends Genet. 2014 5 1;30(5):199–209. 10.1016/j.tig.2014.03.002 24768041

[32] Faast R, Thonglairoam V, Schulz TC, Beall J, Wells JR, Taylor H, et al Histone variant H2A.Z is required for early mammalian development. Curr Biol CB. 2001 8 7;11(15):1183–7. 10.1016/s0960-9822(01)00329-3 11516949

[33] Iouzalen N, Moreau J, Méchali M. H2A.ZI, a new variant histone expressed during Xenopus early development exhibits several distinct features from the core histone H2A. Nucleic Acids Res. 1996 10 15;24(20):3947–52. 10.1093/nar/24.20.3947 8918796 PMC146197

[34] Liu X, Li B, GorovskyMA null. Essential and nonessential histone H2A variants in Tetrahymena thermophila. Mol Cell Biol. 1996 8;16(8):4305–11. 10.1128/mcb.16.8.4305 8754831 PMC231429

[35] Adam M, Robert F, Larochelle M, Gaudreau L. H2A.Z is required for global chromatin integrity and for recruitment of RNA polymerase II under specific conditions. Mol Cell Biol. 2001 9;21(18):6270–9. 10.1128/mcb.21.18.6270-6279.2001 11509669 PMC87352

[36] Jackson JD, Gorovsky MA. Histone H2A.Z has a conserved function that is distinct from that of the major H2A sequence variants. Nucleic Acids Res. 2000 10 1;28(19):3811–6. 10.1093/nar/28.19.3811 11000274 PMC110762

[37] van Attikum H, Fritsch O, Gasser SM. Distinct roles for SWR1 and INO80 chromatin remodeling complexes at chromosomal double-strand breaks. EMBO J. 2007 9 19;26(18):4113–25. 10.1038/sj.emboj.7601835 17762868 PMC2230671

[38] Gerhold CB, Gasser SM. INO80 and SWR complexes: relating structure to function in chromatin remodeling. Trends Cell Biol. 2014 11;24(11):619–31. 10.1016/j.tcb.2014.06.004 25088669

[39] Morrison AJ, Shen X. Chromatin remodelling beyond transcription: the INO80 and SWR1 complexes. Nat Rev Mol Cell Biol. 2009 6;10(6):373–84. 10.1038/nrm2693 19424290 PMC6103619

[40] Tosi A, Haas C, Herzog F, Gilmozzi A, Berninghausen O, Ungewickell C, et al Structure and subunit topology of the INO80 chromatin remodeler and its nucleosome complex. Cell. 2013 9 12;154(6):1207–19. 10.1016/j.cell.2013.08.016 24034245

[41] Clapier CR, Cairns BR. The biology of chromatin remodeling complexes. Annu Rev Biochem. 2009;78:273–304. 10.1146/annurev.biochem.77.062706.153223 19355820

[42] Chen M, Licon K, Otsuka R, Pillus L, Ideker T. Decoupling epigenetic and genetic effects through systematic analysis of gene position. Cell Rep. 2013 1 31;3(1):128–37. 10.1016/j.celrep.2012.12.003 23291096 PMC3563736

[43] Chen Z, Ponts N. H2A.Z and chromatin remodelling complexes: a focus on fungi. Crit Rev Microbiol. 2020 5;46(3):321–37. 10.1080/1040841X.2020.1781784 32594818

[44] Ku M, Jaffe JD, Koche RP, Rheinbay E, Endoh M, Koseki H, et al H2A.Z landscapes and dual modifications in pluripotent and multipotent stem cells underlie complex genome regulatory functions. Genome Biol. 2012 10 3;13(10):R85 10.1186/gb-2012-13-10-r85 23034477 PMC3491413

[45] Kumar SV. H2A.Z at the Core of Transcriptional Regulation in Plants. Mol Plant. 2018 Sep;11(9):1112–4. 10.1016/j.molp.2018.07.002 30053606

[46] Ryan DP, Tremethick DJ. The interplay between H2A.Z and H3K9 methylation in regulating HP1α binding to linker histone-containing chromatin. Nucleic Acids Res. 2018 10 12;46(18):9353–66. 10.1093/nar/gky632 30007360 PMC6182156

[47] Creyghton MP, Markoulaki S, Levine SS, Hanna J, Lodato MA, Sha K, et al H2AZ Is Enriched at Polycomb Complex Target Genes in ES Cells and Is Necessary for Lineage Commitment. Cell. 2008 11 14;135(4):649–61. 10.1016/j.cell.2008.09.056 18992931 PMC2853257

[48] Liu Y, Liu N, Yin Y, Chen Y, Jiang J, Ma Z. Histone H3K4 methylation regulates hyphal growth, secondary metabolism and multiple stress responses in Fusarium graminearum. Environ Microbiol. 2015 11;17(11):4615–30. 10.1111/1462-2920.12993 26234386

[49] Studt L, Janevska S, Arndt B, Boedi S, Sulyok M, Humpf H-U, et al Lack of the COMPASS Component Ccl1 Reduces H3K4 Trimethylation Levels and Affects Transcription of Secondary Metabolite Genes in Two Plant–Pathogenic Fusarium Species. Front Microbiol [Internet]. 2017 [cited 2020 Jul 31];7 Available from: 10.3389/fmicb.2016.02144 28119673 PMC5220078

[50] Bachleitner S, Sørensen JL, Gacek-Matthews A, Sulyok M, Studt L, Strauss J. Evidence of a Demethylase-Independent Role for the H3K4-Specific Histone Demethylases in Aspergillus nidulans and Fusarium graminearum Secondary Metabolism. Front Microbiol [Internet]. 2019 [cited 2019 Nov 15];10 Available from: https://www.frontiersin.org/articles/10.3389/fmicb.2019.01759/full#h4 31456754 10.3389/fmicb.2019.01759 PMC6700381

[51] Connolly LR, Smith KM, Freitag M. The Fusarium graminearum Histone H3 K27 Methyltransferase KMT6 Regulates Development and Expression of Secondary Metabolite Gene Clusters. Madhani HD, editor. PLoS Genet. 2013 10 31;9(10):e1003916 10.1371/journal.pgen.1003916 24204317 PMC3814326

[52] Dong Q, Wang Y, Qi S, Gai K, He Q, Wang Y. Histone variant H2A.Z antagonizes the positive effect of the transcriptional activator CPC1 to regulate catalase-3 expression under normal and oxidative stress conditions. Free Radic Biol Med. 2018 6 1;121:136–48. 10.1016/j.freeradbiomed.2018.05.003 29738831

[53] Cui G, Dong Q, Duan J, Zhang C, Liu X, He Q. NC2 complex is a key factor for the activation of catalase-3 transcription by regulating H2A.Z deposition. Nucleic Acids Res [Internet]. 2020 [cited 2020 Jul 31]; Available from: 10.1093/nar/gkaa552 32633757 PMC7470962

[54] Courtney AJ, Kamei M, Ferraro AR, Gai K, He Q, Honda S, et al Normal Patterns of Histone H3K27 Methylation Require the Histone Variant H2A.Z in Neurospora crassa. Genetics. 2020 7 10; 10.1534/genetics.120.303442 32651262 PMC7463285

[55] Altschul SF, Madden TL, Schäffer AA, Zhang J, Zhang Z, Miller W, et al Gapped BLAST and PSI-BLAST: a new generation of protein database search programs. Nucleic Acids Res. 1997 9 1;25(17):3389–402. 10.1093/nar/25.17.3389 9254694 PMC146917

[56] Liu H, Wang Q, He Y, Chen L, Hao C, Jiang C, et al Genome-wide A-to-I RNA editing in fungi independent of ADAR enzymes. Genome Res. 2016 4;26(4):499–509. 10.1101/gr.199877.115 26934920 PMC4817773

[57] Bui D-C, Lee Y, Lim JY, Fu M, Kim J-C, Choi GJ, et al Heat shock protein 90 is required for sexual and asexual development, virulence, and heat shock response in Fusarium graminearum. Sci Rep. 2016 16;6:28154 10.1038/srep28154 27306495 PMC4910114

[58] Zhao C, Waalwijk C, de Wit PJGM, Tang D, van der Lee T. Relocation of genes generates non-conserved chromosomal segments in Fusarium graminearum that show distinct and co-regulated gene expression patterns. BMC Genomics. 2014 3 13;15:191 10.1186/1471-2164-15-191 24625133 PMC4022177

[59] Hays SM, Swanson J, Selker EU. Identification and characterization of the genes encoding the core histones and histone variants of Neurospora crassa. Genetics. 2002 3;160(3):961–73. 11901114 10.1093/genetics/160.3.961 PMC1462028

[60] May GS, Morris NR. The unique histone H2A gene of Aspergillus nidulans contains three introns. Gene. 1987;58(1):59–66. 10.1016/0378-1119(87)90029-1 3319784

[61] Kurat CF, Recht J, Radovani E, Durbic T, Andrews B, Fillingham J. Regulation of histone gene transcription in yeast. Cell Mol Life Sci CMLS. 2014 2;71(4):599–613. 10.1007/s00018-013-1443-9 23974242 PMC11113579

[62] Merhej J, Urban M, Dufresne M, Hammond-Kosack KE, Richard-Forget F, Barreau C. The velvet gene, FgVe1, affects fungal development and positively regulates trichothecene biosynthesis and pathogenicity in Fusarium graminearum. Mol Plant Pathol. 2012 5;13(4):363–74. 10.1111/j.1364-3703.2011.00755.x 22013911 PMC6638759

[63] Ponts N, Pinson-Gadais L, Verdal-Bonnin M-N, Barreau C, Richard-Forget F. Accumulation of deoxynivalenol and its 15-acetylated form is significantly modulated by oxidative stress in liquid cultures of Fusarium graminearum: DON-ADON accumulation is modulated by oxidative stress. FEMS Microbiol Lett. 2006 5;258(1):102–7. 10.1111/j.1574-6968.2006.00200.x 16630263

[64] Liu X, Dang Y, Matsu-Ura T, He Y, He Q, Hong CI, et al DNA Replication Is Required for Circadian Clock Function by Regulating Rhythmic Nucleosome Composition. Mol Cell. 2017 7 20;67(2):203–213.e4. 10.1016/j.molcel.2017.05.029 28648778 PMC5543814

[65] Kim H-S, Vanoosthuyse V, Fillingham J, Roguev A, Watt S, Kislinger T, et al An acetylated form of histone H2A.Z regulates chromosome architecture in Schizosaccharomyces pombe. Nat Struct Mol Biol. 2009 12;16(12):1286–93. 10.1038/nsmb.1688 19915592 PMC2788674

[66] Ponts N. Mycotoxins are a component of Fusarium graminearum stress-response system. Front Microbiol [Internet]. 2015 11 4 [cited 2019 Aug 1];6. Available from: http://journal.frontiersin.org/Article/10.3389/fmicb.2015.01234/abstract 26583017 10.3389/fmicb.2015.01234 PMC4631952

[67] Seong K-Y, Pasquali M, Zhou X, Song J, Hilburn K, McCormick S, et al Global gene regulation by Fusarium transcription factors Tri6 and Tri10 reveals adaptations for toxin biosynthesis. Mol Microbiol. 2009 4;72(2):354–67. 10.1111/j.1365-2958.2009.06649.x 19320833

[68] Davis BH, Poon AFY, Whitlock MC. Compensatory mutations are repeatable and clustered within proteins. Proc R Soc B Biol Sci. 2009 5 22;276(1663):1823–7. 10.1098/rspb.2008.1846 19324785 PMC2674493

[69] Marchler-Bauer A, Bo Y, Han L, He J, Lanczycki CJ, Lu S, et al CDD/SPARCLE: functional classification of proteins via subfamily domain architectures. Nucleic Acids Res. 2017 04;45(D1):D200–3. 10.1093/nar/gkw1129 27899674 PMC5210587

[70] Lu S, Wang J, Chitsaz F, Derbyshire MK, Geer RC, Gonzales NR, et al CDD/SPARCLE: the conserved domain database in 2020. Nucleic Acids Res. 2020 1 8;48(D1):D265–8. 10.1093/nar/gkz991 31777944 PMC6943070

[71] Wagner EJ, Carpenter PB. Understanding the language of Lys36 methylation at histone H3. Nat Rev Mol Cell Biol. 2012 2;13(2):115–26. 10.1038/nrm3274 22266761 PMC3969746

[72] Woo H, Ha SD, Lee SB, Buratowski S, Kim T. Modulation of gene expression dynamics by co-transcriptional histone methylations. Exp Mol Med. 2017 4;49(4):e326–e326. 10.1038/emm.2017.19 28450734 PMC6130219

[73] Bošković A, Bender A, Gall L, Ziegler-Birling C, Beaujean N, Torres-Padilla M-E. Analysis of active chromatin modifications in early mammalian embryos reveals uncoupling of H2A.Z acetylation and H3K36 trimethylation from embryonic genome activation. Epigenetics. 2012 7 1;7(7):747–57. 10.4161/epi.20584 22647320

[74] Liao S-M, Du Q-S, Meng J-Z, Pang Z-W, Huang R-B. The multiple roles of histidine in protein interactions. Chem Cent J. 2013 3 1;7:44 10.1186/1752-153X-7-44 23452343 PMC3599372

[75] Morris AL, MacArthur MW, Hutchinson EG, Thornton JM. Stereochemical quality of protein structure coordinates. Proteins Struct Funct Bioinforma. 1992;12(4):345–64. 10.1002/prot.340120407 1579569

[76] Filteau M, Hamel V, Pouliot M-C, Gagnon-Arsenault I, Dubé AK, Landry CR. Evolutionary rescue by compensatory mutations is constrained by genomic and environmental backgrounds. Mol Syst Biol [Internet]. 2015 10 12 [cited 2019 Aug 9];11(10). Available from: https://www.ncbi.nlm.nih.gov/pmc/articles/PMC4631203/ 10.15252/msb.20156444 26459777 PMC4631203

[77] Laurent B, Moinard M, Spataro C, Ponts N, Barreau C, Foulongne-Oriol M. Landscape of genomic diversity and host adaptation in Fusarium graminearum. BMC Genomics. 2017 23;18(1):203 10.1186/s12864-017-3524-x 28231761 PMC5324198

[78] Cuomo CA, Güldener U, Xu J-R, Trail F, Turgeon BG, Di Pietro A, et al The Fusarium graminearum genome reveals a link between localized polymorphism and pathogen specialization. Science. 2007 9 7;317(5843):1400–2. 10.1126/science.1143708 17823352

[79] King R, Urban M, Hammond-Kosack MCU, Hassani-Pak K, Hammond-Kosack KE. The completed genome sequence of the pathogenic ascomycete fungus Fusarium graminearum. BMC Genomics. 2015 7 22;16:544 10.1186/s12864-015-1756-1 26198851 PMC4511438

[80] King R, Urban M, Hammond-Kosack KE. Annotation of Fusarium graminearum (PH-1) Version 5.0. Genome Announc. 2017 1 12;5(2). 10.1128/genomeA.01479-16 28082505 PMC5256205

[81] Stajich JE, Harris T, Brunk BP, Brestelli J, Fischer S, Harb OS, et al FungiDB: an integrated functional genomics database for fungi. Nucleic Acids Res. 2012 1;40(Database issue):D675–681. 10.1093/nar/gkr918 22064857 PMC3245123

[82] Basenko EY, Pulman JA, Shanmugasundram A, Harb OS, Crouch K, Starns D, et al FungiDB: An Integrated Bioinformatic Resource for Fungi and Oomycetes. J Fungi. 2018 3;4(1):39 10.3390/jof4010039 30152809 PMC5872342

[83] Ruan K, Yamamoto TG, Asakawa H, Chikashige Y, Kimura H, Masukata H, et al Histone H4 acetylation required for chromatin decompaction during DNA replication. Sci Rep. 2015 7 30;5:12720 10.1038/srep12720 26223950 PMC4520004

[84] Hanes SD. Prolyl isomerases in gene transcription. Biochim Biophys Acta. 2015 10;1850(10):2017–34. 10.1016/j.bbagen.2014.10.028 25450176 PMC4417086

[85] Nelson CJ, Santos-Rosa H, Kouzarides T. Proline Isomerization of Histone H3 Regulates Lysine Methylation and Gene Expression. Cell. 2006 9 8;126(5):905–16. 10.1016/j.cell.2006.07.026 16959570

[86] Zhang Y, Shan C-M, Wang J, Bao K, Tong L, Jia S. Molecular basis for the role of oncogenic histone mutations in modulating H3K36 methylation. Sci Rep. 2017 03;7:43906 10.1038/srep43906 28256625 PMC5335568

[87] Rogawski DS, Ndoj J, Cho HJ, Maillard I, Grembecka J, Cierpicki T. Two Loops Undergoing Concerted Dynamics Regulate the Activity of the ASH1L Histone Methyltransferase. Biochemistry. 2015 9 8;54(35):5401–13. 10.1021/acs.biochem.5b00697 26292256 PMC4664444

[88] Kavanagh KL, Jörnvall H, Persson B, Oppermann U. Medium- and short-chain dehydrogenase/reductase gene and protein families: the SDR superfamily: functional and structural diversity within a family of metabolic and regulatory enzymes. Cell Mol Life Sci CMLS. 2008 12;65(24):3895–906. 10.1007/s00018-008-8588-y 19011750 PMC2792337

[89] Persson B, Kallberg Y, Oppermann U, Jörnvall H. Coenzyme-based functional assignments of short-chain dehydrogenases/reductases (SDRs). Chem Biol Interact. 2003 2 1;143–144:271–8. 10.1016/s0009-2797(02)00223-5 12604213

[90] Oppermann U, Filling C, Hult M, Shafqat N, Wu X, Lindh M, et al Short-chain dehydrogenases/reductases (SDR): the 2002 update. Chem Biol Interact. 2003 2 1;143–144:247–53. 10.1016/s0009-2797(02)00164-3 12604210

[91] Kleiger G, Eisenberg D. GXXXG and GXXXA motifs stabilize FAD and NAD(P)-binding Rossmann folds through C(alpha)-H… O hydrogen bonds and van der waals interactions. J Mol Biol. 2002 10 11;323(1):69–76. 10.1016/s0022-2836(02)00885-9 12368099

[92] Wang Q, Jiang C, Wang C, Chen C, Xu J-R, Liu H. Characterization of the Two-Speed Subgenomes of Fusarium graminearum Reveals the Fast-Speed Subgenome Specialized for Adaption and Infection. Front Plant Sci. 2017;8:140 10.3389/fpls.2017.00140 28261228 PMC5306128

[93] Secombe J, Eisenman RN. The function and regulation of the JARID1 family of histone H3 lysine 4 demethylases: the Myc connection. Cell Cycle Georget Tex. 2007 6 1;6(11):1324–8. 10.4161/cc.6.11.4269 17568193

[94] Horton JR, Engstrom A, Zoeller EL, Liu X, Shanks JR, Zhang X, et al Characterization of a Linked Jumonji Domain of the KDM5/JARID1 Family of Histone H3 Lysine 4 Demethylases. J Biol Chem. 2016 2 5;291(6):2631–46. 10.1074/jbc.M115.698449 26645689 PMC4742734

[95] Gacek-Matthews A, Berger H, Sasaki T, Wittstein K, Gruber C, Lewis ZA, et al KdmB, a Jumonji Histone H3 Demethylase, Regulates Genome-Wide H3K4 Trimethylation and Is Required for Normal Induction of Secondary Metabolism in Aspergillus nidulans. PLOS Genet. 2016 8 22;12(8):e1006222 10.1371/journal.pgen.1006222 27548260 PMC4993369

[96] Janevska S, Güldener U, Sulyok M, Tudzynski B, Studt L. Set1 and Kdm5 are antagonists for H3K4 methylation and regulators of the major conidiation-specific transcription factor gene ABA1 in Fusarium fujikuroi. Environ Microbiol. 2018;20(9):3343–62. 10.1111/1462-2920.14339 30047187 PMC6175112

[97] Wiemann P, Sieber CMK, von Bargen KW, Studt L, Niehaus EM, Espino JJ, et al Deciphering the cryptic genome: genome-wide analyses of the rice pathogen *Fusarium fujikuroi* reveal complex regulation of secondary metabolism and novel metabolites. PLoS Pathog. 2013;9(6):e1003475 10.1371/journal.ppat.1003475 23825955 PMC3694855

[98] Bömke C, Tudzynski B. Diversity, regulation, and evolution of the gibberellin biosynthetic pathway in fungi compared to plants and bacteria. Phytochemistry. 2009 10 1;70(15):1876–93. 19560174 10.1016/j.phytochem.2009.05.020

[99] Yu C, Zavaljevski N, Desai V, Reifman J. QuartetS: A fast and accurate algorithm for large-scale orthology detection [Internet]. Vol. 39, Nucleic Acids Research. 2011 [cited 2020 May 18]. Available from: https://www.ncbi.nlm.nih.gov/pmc/articles/PMC3141274/ 10.1093/nar/gkr308 21572104 PMC3141274

[100] Teng X, Dayhoff-Brannigan M, Cheng W-C, Gilbert CE, Sing CN, Diny NL, et al Genome-wide consequences of deleting any single gene. Mol Cell. 2013 11 21;52(4):485–94. 10.1016/j.molcel.2013.09.026 24211263 PMC3975072

[101] Rojas Echenique JI, Kryazhimskiy S, Nguyen Ba AN, Desai MM. Modular epistasis and the compensatory evolution of gene deletion mutants. Butler G, editor. PLOS Genet. 2019 2 15;15(2):e1007958 10.1371/journal.pgen.1007958 30768593 PMC6395002

[102] Li X, Fan Z, Yan M, Qu J, Xu J-R, Jin Q. Spontaneous mutations in FgSAD1 suppress the growth defect of the Fgprp4 mutant by affecting tri-snRNP stability and its docking in Fusarium graminearum. Environ Microbiol. 2019;21(12):4488–503. 10.1111/1462-2920.14736 31291045

[103] Ilgen P, Hadeler B, Maier FJ, Schäfer W. Developing kernel and rachis node induce the trichothecene pathway of Fusarium graminearum during wheat head infection. Mol Plant-Microbe Interact MPMI. 2009 8;22(8):899–908. 10.1094/MPMI-22-8-0899 19589066

[104] Cappellini RA, Peterson JL. Macroconidium Formation in Submerged Cultures by a Non-Sporulating Strain of Gibberella zeae. Mycologia. 1965;57(6):962–6.

[105] Boutigny A-L, Barreau C, Atanasova-Penichon V, Verdal-Bonnin M-N, Pinson-Gadais L, Richard-Forget F. Ferulic acid, an efficient inhibitor of type B trichothecene biosynthesis and Tri gene expression in Fusarium liquid cultures. Mycol Res. 2009 6 1;113(6):746–53. 10.1016/j.mycres.2009.02.010 19249362

[106] Darken MA, Jensen AL, Shu P. Production of gibberellic acid by fermentation. Appl Microbiol. 1959;7(12):301–3. 13814121 10.1128/am.7.5.301-303.1959 PMC1057525

[107] Geissman TA, Verbiscar AJ, Phinney BO, Cragg G. Studies on the biosynthesis of gibberellins from (-)-kaurenoic acid in cultures of Gibberella Fujikuroi. Phytochemistry. 1966;5(5):933–47.

[108] Pontecorvo G, Roper JA, Chemmons LM, Macdonald KD, Bufton AWJ. The Genetics of Aspergillus nidulans. Adv Genet. 1953; 10.1016/s0065-2660(08)60408-3 13040135

[109] Schumacher J. Tools for Botrytis cinerea: New expression vectors make the gray mold fungus more accessible to cell biology approaches. Fungal Genet Biol. 2012;49(6):483–97. 10.1016/j.fgb.2012.03.005 22503771

[110] Ponts N, Yang J, Chung D-WD, Prudhomme J, Girke T, Horrocks P, et al Deciphering the ubiquitin-mediated pathway in apicomplexan parasites: a potential strategy to interfere with parasite virulence. PloS One. 2008 6 11;3(6):e2386 10.1371/journal.pone.0002386 18545708 PMC2408969

[111] Altschul SF, Wootton JC, Gertz EM, Agarwala R, Morgulis A, Schäffer AA, et al Protein database searches using compositionally adjusted substitution matrices. FEBS J. 2005 10;272(20):5101–9. 10.1111/j.1742-4658.2005.04945.x 16218944 PMC1343503

[112] Catlett N, Lee B-N, Yoder O, Turgeon B. Split-Marker Recombination for Efficient Targeted Deletion of Fungal Genes. Fungal Genet Rep. 2003 12 1;50(1):9–11.

[113] Montibus M, Ducos C, Bonnin-Verdal M-N, Bormann J, Ponts N, Richard-Forget F, et al The bZIP Transcription Factor Fgap1 Mediates Oxidative Stress Response and Trichothecene Biosynthesis But Not Virulence in Fusarium graminearum. Lee Y-W, editor. PLoS ONE. 2013 12 12;8(12):e83377 10.1371/journal.pone.0083377 24349499 PMC3861502

[114] Collopy PD, Colot HV, Park G, Ringelberg C, Crew CM, Borkovich KA, et al High-throughput construction of gene deletion cassettes for generation of Neurospora crassa knockout strains. Methods Mol Biol Clifton NJ. 2010;638:33–40. 10.1007/978-1-60761-611-5_3 20238259 PMC3684412

[115] Colot H V., Park G, Turner GE, Ringelberg C, Crew CM, Litvinkova L, et al A high-throughput gene knockout procedure for Neurospora reveals functions for multiple transcription factors. Proc Natl Acad Sci. 2006;103(27):10352–7. 10.1073/pnas.0601456103 16801547 PMC1482798

[116] Staben C, Jensen B, Singer M, Pollock J, Schechtman M, Kinsey J, et al Use of a bacterial hygromycin B resistance gene as a dominant selectable marker in Neurospora crassa transformation. Fungal Genet Rep. 2017;

[117] Niehaus E-M, Münsterkötter M, Proctor RH, Brown DW, Sharon A, Idan Y, et al Comparative “Omics” of the Fusarium fujikuroi Species Complex Highlights Differences in Genetic Potential and Metabolite Synthesis. Genome Biol Evol. 2016 12 31;8(11):3574–99. 10.1093/gbe/evw259 28040774 PMC5203792

[118] Tudzynski B, Homann V, Feng B, Marzluf G a. Isolation, characterization and disruption of the *areA* nitrogen regulatory gene of *Gibberella fujikuroi*. Mol Gen Genet MGG. 1999;261:106–14. 10.1007/s004380050947 10071216

[119] Schneider CA, Rasband WS, Eliceiri KW. NIH Image to ImageJ: 25 years of image analysis. Nat Methods. 2012 7;9(7):671–5. 10.1038/nmeth.2089 22930834 PMC5554542

[120] Cavinder B, Sikhakolli U, Fellows KM, Trail F. Sexual Development and Ascospore Discharge in Fusarium graminearum. J Vis Exp JoVE [Internet]. 2012 3 29 [cited 2019 Aug 27];(61). Available from: https://www.ncbi.nlm.nih.gov/pmc/articles/PMC3460587/ 10.3791/3895 22491175 PMC3460587

[121] Javerzat JP, Bhattacherjee V, Barreau C. Isolation of telomeric DNA from the filamentous fungus Podospora anserina and construction of a self-replicating linear plasmid showing high transformation frequency. Nucleic Acids Res. 1993 2 11;21(3):497–504. 10.1093/nar/21.3.497 8441663 PMC309145

[122] Bolger A, Scossa F, Bolger ME, Lanz C, Maumus F, Tohge T, et al The genome of the stress-tolerant wild tomato species *Solanum pennellii*. Nat Genet. 2014 9;46(9):1034–8. 10.1038/ng.3046 25064008 PMC7036041

