# Supplementary material for: Effect of H2A.Z deletion is rescued by compensatory mutations in Fusarium graminearum
Source: PLoS Genet. 2020 Oct 22;16(10):e1009125. doi: 10.1371/journal.pgen.1009125 (PMC7608984; doi:10.1371/journal.pgen.1009125)
Supplement: S1 Text — (DOCX) [file pgen.1009125.s001.docx]

**Supporting Information for:**

**Effect of *H2A*.Z deletion is rescued by compensatory mutations in *Fusarium graminearum***

Zhenhui Chen^1^, Enric Zehraoui^1^, Anna K. Atanasoff-Kardjalieff^2^, Joseph Strauss^2^, Lena Studt^2^, Nadia Ponts^1,*^

^1^INRAE, MycSA, F-33882 Villenave d’Ornon, France

^2^Department of Applied Genetics and Cell Biology, University of Natural Resources and Life Sciences, Vienna (BOKU), Vienna, Austria

* To whom correspondence should be addressed. **Email: nadia.ponts@inrae.fr**


**Additional Materials and Methods**

PCR and Southern Blot validation of deletion mutant

Genomic DNA was extracted from 50 mg of freeze-dried mycelium, from INRA349 wild-type and I349∆*H2A.Z* mutant strains, as previously described [5]. PCR was performed using the Phusion^TM^ High-Fidelity DNA polymerase (ThermoFisher Scientific) as follows: 98°C for 30 s, 35 × [98°C for 10 s, 68°C for 30 s, 72°C for 15 s/kb], 72°C for 5 min using primer pair 5f-UTR-H2AZ-F and 3r-3UTR-H2AZ-R (see **Table S2** and **Figure S3A**). Southern blot was performed using the DIG system (Roche) according to a previously published protocol [6]. Briefly, 5 µg of gDNA from INRA349 wild-type or I349∆*H2A.Z* was digested with *NdeI* (ThermoFisher Scientific) and separated by 0.8% agarose gel electrophoresis. After transfer onto nylon membranes (Hybond N+, Amersham), a DIG-labeled probe matching the upstream region of the native *H2A*.Z *locus* and part of its CDS (**Figure S3C**) was synthesized with a PCR DIG probe synthesis kit (Roche) using the primer pair 5f-UTR-H2AZ-F (**Table S2**) and 5’- CCTCAAGGTCAAGCGTATCACAC -3’, and hybridized onto the membrane following manufacturer’s instructions.

qPCR assays to evaluate H2A.Z expression levels

RNA extraction and reverse transcription were performed according to a previously published protocol [6]. Regarding qPCR assays, analyses were performed using 1 µL of each cDNA preparation (corresponding to 1 ng of total RNA input in the reverse transcription) mixed in a 10 µL-reaction volume, using the QuantiFast™ SYBR® Green PCR kit (Qiagen). Reactions were carried out on a QuantStudio^TM^ 5 system (Applied Biosystem). For each gene, efficiency was evaluated with serial dilutions of the pooled cDNA samples and data analyses were performed as previously described [7]. Expression levels of Fg*H2A.Z* normalized to the expression of the housekeeping genes ß-tubulin, ubiquitin hydrolase, and eF1 were performed using the the QuantStudio™ Design & Analysis Software, Version 2.4 (Applied Biosystems). Primers used can be found in Table S1.

Standard molecular techniques for *F. fujikuroi*

For DNA isolation, lyophilized mycelium was ground to a fine powder, re-suspended in extraction buffer and isolated as previously described [8]. Isolated gDNA was used for PCR amplification according to the manufacturers’ instruction. For diagnostic PCR, the GoTaq® G2 DNA Polymerase (Promega) was used and the PCR reactions were set up according to the users’ manual. Generated plasmids were extracted and purified from *S. cerevisiae* with the GeneJET^TM^ plasmid miniprep kit (Fermentas GmbH, St. Leon-Rot, Germany).

**Additional references**

1. Zhao C, Waalwijk C, de Wit PJGM, Tang D, van der Lee T. Relocation of genes generates non-conserved chromosomal segments in Fusarium graminearum that show distinct and co-regulated gene expression patterns. BMC Genomics. 2014 Mar 13;15:191.

2. Suto RK, Clarkson MJ, Tremethick DJ, Luger K. Crystal structure of a nucleosome core particle containing the variant histone H2A.Z. Nat Struct Biol. 2000 Dec;7(12):1121–4.

3. Basenko EY, Pulman JA, Shanmugasundram A, Harb OS, Crouch K, Starns D, et al. FungiDB: An Integrated Bioinformatic Resource for Fungi and Oomycetes. Journal of Fungi. 2018 Mar;4(1):39.

4. Stajich JE, Harris T, Brunk BP, Brestelli J, Fischer S, Harb OS, et al. FungiDB: an integrated functional genomics database for fungi. Nucleic Acids Res. 2012 Jan;40(Database issue):D675-681.

5. Javerzat JP, Bhattacherjee V, Barreau C. Isolation of telomeric DNA from the filamentous fungus Podospora anserina and construction of a self-replicating linear plasmid showing high transformation frequency. Nucleic Acids Res. 1993 Feb 11;21(3):497–504.

6. Montibus M, Ducos C, Bonnin-Verdal M-N, Bormann J, Ponts N, Richard-Forget F, et al. The bZIP transcription factor Fgap1 mediates oxidative stress response and trichothecene biosynthesis but not virulence in Fusarium graminearum. PLoS ONE. 2013;8(12):e83377.

7. Ponts N, Pinson-Gadais L, Barreau C, Richard-Forget F, Ouellet T. Exogenous H2O2 and catalase treatments interfere with Tri genes expression in liquid cultures of Fusarium graminearum. FEBS Letters. 2007;581(3):443–7.

8. Cenis JL. Rapid extraction of fungal DNA for PCR amplification. Nucleic acids research. 1992;20(9):2380.
