# Supplementary material for: Effect of H2A.Z deletion is rescued by compensatory mutations in Fusarium graminearum
Source: PLoS Genet. 2020 Oct 22;16(10):e1009125. doi: 10.1371/journal.pgen.1009125 (PMC7608984; doi:10.1371/journal.pgen.1009125)
Supplement: S1 File — (PDF) [file pgen.1009125.s002.pdf]

BLASTP 2.10.1+
Reference: Stephen F. Altschul, Thomas L. Madden, Alejandro A. Schäffer,
Jinghui Zhang, Zheng Zhang, Webb Miller, and David J. Lipman (1997), "Gapped
BLAST and PSI-BLAST: a new generation of protein database search programs",
Nucleic Acids Res. 25:3389-3402.

RID: HE08R7VM016

Database: refseq_protein
Query= FGRAMPH1_01T26109
Length=134

Sequences producing significant alignments:		Score (Bits)	E Value
ref|XP_009259038.1|	hypothetical protein FPSE_07645 [Fusarium...	179	3E-55
ref|XP_018064945.1|	histone-fold-containing protein [Phialoce...	175	7E-54
ref|XP_024744074.1|	histone-fold-containing protein [Hyaloscy...	175	9E-54
ref|XP_007294773.1|	histone H2A [Marssonina brunnea f. sp. 'm...	174	1E-53
ref|XP_033461959.1|	histone H2A [Dissoconium aciculare CBS 34...	174	1E-53
ref|XP_003344381.1|	putative HTA2 protein [Sordaria macrospor...	174	2E-53
ref|XP_003051923.1|	histone 2A [Fusarium vanettenii 77-13-4] ...	174	2E-53
ref|XP_018184446.1|	histone-fold-containing protein [Xylona h...	174	3E-53
ref|XP_022475722.1|	histone H2A [Colletotrichum orchidophilum...	174	3E-53
ref|XP_008100773.1|	histone H2A [Colletotrichum graminicola M...	174	3E-53
ref|XP_002999953.1|	histone H2A [Verticillium alfalfae VaMs.1...	174	3E-53
ref|XP_002486399.1|	histone H2A [Talaromyces stipitatus ATCC ...	173	4E-53
ref|XP_022582571.1|	hypothetical protein ASPZODRAFT_129982 [P...	173	6E-53
ref|XP_003654109.1|	histone H2A-like protein [Thermothielavio...	173	6E-53
ref|XP_018181861.1|	histone H2A [Purpureocillium lilacinum] >...	173	6E-53
ref|XP_013332342.1|	Histone H2A [Rasamsonia emersonii CBS 393...	173	6E-53
ref|XP_033532232.1|	histone 2A [Eremomyces bilateralis CBS 78...	173	7E-53
ref|XP_031005213.1|	Histone H2A [Lachnellula hyalina] >gb|TVY...	173	7E-53

```
ref|XP_001550543.1|  hypothetical protein BCIN_02g06800 [Botry...   173    8E-53
ref|XP_001263909.1|  histone h2a [Aspergillus fischeri NRRL 18...   172    9E-53
ref|XP_022400783.1|  hypothetical protein ASPGLDRAFT_46875 [As...   172    9E-53
ref|XP_033400410.1|  uncharacterized protein K452DRAFT_306480 ...   172    9E-53
ref|XP_033557660.1|  histone H2A [Macroventuria anomochaeta] >...   172    9E-53
ref|XP_009158759.1|  histone H2A [Exophiala dermatitidis NIH/U...   172    9E-53
ref|XP_030999825.1|  uncharacterized protein E0L32_002623 [Phi...   172    1E-52
ref|XP_009221324.1|  histone H2A [Gaeumannomyces tritici R3-11...   172    1E-52
ref|XP_006695183.1|  hypothetical protein CTHT_0048200 [Chaeto...   172    1E-52
ref|XP_016582554.1|  histone H2A [Sporothrix schenckii 1099-18...   172    1E-52
ref|XP_018143750.1|  histone H2A [Pochonia chlamydosporia 170]...   172    1E-52
ref|XP_006964898.1|  histone H2A [Trichoderma reesei QM6a] >re...   172    1E-52
ref|XP_024717498.1|  hypothetical protein M430DRAFT_53732 [Amo...   172    1E-52
ref|XP_016643417.1|  hypothetical protein SAPIO_CDS4549 [Scedo...   172    1E-52
ref|XP_013957466.1|  hypothetical protein TRIVIDRAFT_215760 [T...   172    1E-52
ref|XP_008078485.1|  Histone-fold containing protein [Glarea l...   172    1E-52
ref|XP_002152696.1|  histone H2A [Talaromyces marneffei ATCC 1...   172    1E-52
ref|XP_024769015.1|  hypothetical protein M431DRAFT_154042 [Tr...   172    1E-52
ref|XP_007806467.1|  histone H2A [Metarhizium acridum CQMa 102...   172    1E-52
ref|XP_003716333.1|  histone H2A [Pyricularia oryzae 70-15] >r...   172    1E-52
ref|XP_031866196.1|  Histone H2A [Venustampulla echinocandica]...   172    2E-52
ref|XP_018153697.1|  Histone H2A [Colletotrichum higginsianum ...   172    2E-52
ref|XP_035320237.1|  histone H2A [Geosmithia morbida] >gb|KAF4...   171    2E-52
ref|XP_001217588.1|  histone H2A [Aspergillus terreus NIH2624]...   171    2E-52
ref|XP_035350572.1|  uncharacterized protein TRUGW13939_11573 ...   171    2E-52
ref|XP_006666427.1|  histone H2A [Cordyceps militaris CM01] >r...   171    2E-52
ref|XP_013321709.1|  histone H2A [Exophiala xenobiotica] >ref|...   171    2E-52
ref|XP_020122957.1|  Histone H2A [Talaromyces atroroseus] >gb|...   171    2E-52
ref|XP_001929713.1|  uncharacterized protein PODANS_5_5390 [Po...   171    2E-52
ref|XP_024707064.1|  putative histone H2A [Aspergillus steynii...   171    2E-52
ref|XP_033436560.1|  Histone H2A [Daldinia childiae] >gb|KAF30...   171    2E-52
ref|XP_020130993.1|  histone h2a [Diplodia corticola] >ref|XP_...   171    2E-52
```

ALIGNMENTS

```
>XP_009259038.1| hypothetical protein FPSE_07645 [Fusarium pseudograminearum CS3096]
 ref|XP_011327819.1| hypothetical protein FGSG_11627 [Fusarium graminearum PH-1]
 ref|XP_025582531.1| uncharacterized protein FVRRES_12833 [Fusarium venenatum]
 sp|Q4HTT1.3| RecName: Full=Histone H2A [Fusarium graminearum PH-1]
 gb|EYB31561.1| hypothetical protein FG05_11627 [Fusarium graminearum]
 gb|KAF0639505.1| hypothetical protein FPSE5266_07645 [Fusarium pseudograminearum]
 gb|KAF5240219.1| hypothetical protein FAUST_4383 [Fusarium austroamericanum]
 gb|KPA42452.1| histone h2a [Fusarium langsethiae]
 gb|OBS19194.1| hypothetical protein FPOA_10918 [Fusarium poae]
 gb|PTD01533.1| Histone H2A [Fusarium culmorum]
 gb|RGP59867.1| histone h2a [Fusarium sporotrichioides]
          Length=134

 Score = 178.7 bits (452),  Expect = 3E-55
 Identities = 119/119 (100%), Positives = 119/119 (100%), Gaps = 0/119 (0%)

Query  16   NAQSRSSKAGLAFPVGRVHRLLRKGNYAQRVGAGAPXXXXXXXXXXXXXXXXXXGNAARD  75
            NAQSRSSKAGLAFPVGRVHRLLRKGNYAQRVGAGAPVYLAAVLEYLAAEILELAGNAARD
Sbjct  16   NAQSRSSKAGLAFPVGRVHRLLRKGNYAQRVGAGAPVYLAAVLEYLAAEILELAGNAARD  75

Query  76   NKKTRIIPRHLQLAIRNDEELNKLLGHVTIAQGGVLPNIHQNLLPXXXXXXXXXSSMEL  134
            NKKTRIIPRHLQLAIRNDEELNKLLGHVTIAQGGVLPNIHQNLLPKKTGKTGKTSSMEL
Sbjct  76   NKKTRIIPRHLQLAIRNDEELNKLLGHVTIAQGGVLPNIHQNLLPKKTGKTGKTSSMEL  134


>XP_018064945.1| histone-fold-containing protein [Phialocephala scopiformis]
 gb|KUJ10590.1| histone-fold-containing protein [Phialocephala scopiformis]
          Length=134

 Score = 174.9 bits (442),  Expect = 7E-54
 Identities = 115/119 (96%), Positives = 116/119 (97%), Gaps = 0/119 (0%)

Query  16   NAQSRSSKAGLAFPVGRVHRLLRKGNYAQRVGAGAPXXXXXXXXXXXXXXXXXXGNAARD  75
            NAQSRSSKAGLAFPVGRVHRLLRKGNYAQRVGAGAPVYLAAVLEYLAAEILELAGNAARD
```

```
Sbjct  16   NAQSRSSKAGLAFPVGRVHRLLRKGNYAQRVGAGAPVYLAAVLEYLAAEILELAGNAARD  75

Query  76   NKKTRIIPRHLQLAIRNDEELNKLLGHVTIAQGGVLPNIHQNLLPXXXXXXXXXSSMEL  134
            NKKTRIIPRHLQLAIRNDEELNKLLGHVTIAQGGVLPNIHQNLLPKKT K GKT+S EL
Sbjct  76   NKKTRIIPRHLQLAIRNDEELNKLLGHVTIAQGGVLPNIHQNLLPKKTAKGGKTASQEL  134


>XP_024744074.1| histone-fold-containing protein [Hyaloscypha bicolor E]
 gb|KAA6408864.1| histone H2A [Lasallia pustulata]
 gb|KAE9369429.1| histone-fold-containing protein [Chalara longipes BDJ]
 gb|PMD14103.1| histone-fold-containing protein [Pezoloma ericae]
 gb|PMD42553.1| histone-fold-containing protein [Hyaloscypha variabilis F]
 gb|PVH71853.1| histone-fold-containing protein [Cadophora sp. DSE1049]
 emb|CZR57393.1| Histone H2A [Phialocephala subalpina]
          Length=133

 Score = 174.9 bits (442),  Expect = 9E-54
 Identities = 115/119 (96%), Positives = 116/119 (97%), Gaps = 0/119 (0%)

Query  16   NAQSRSSKAGLAFPVGRVHRLLRKGNYAQRVGAGAPXXXXXXXXXXXXXXXXXXGNAARD  75
            NAQSRSSKAGLAFPVGRVHRLLRKGNYAQRVGAGAPVYLAAVLEYLAAEILELAGNAARD
Sbjct  15   NAQSRSSKAGLAFPVGRVHRLLRKGNYAQRVGAGAPVYLAAVLEYLAAEILELAGNAARD  74

Query  76   NKKTRIIPRHLQLAIRNDEELNKLLGHVTIAQGGVLPNIHQNLLPXXXXXXXXXSSMEL  134
            NKKTRIIPRHLQLAIRNDEELNKLLGHVTIAQGGVLPNIHQNLLPKKT K GKT+S EL
Sbjct  75   NKKTRIIPRHLQLAIRNDEELNKLLGHVTIAQGGVLPNIHQNLLPKKTAKGGKTASQEL  133


>XP_007294773.1| histone H2A [Marssonina brunnea f. sp. 'multigermtubi' MB_m1]
 gb|EKD15123.1| histone H2A [Marssonina brunnea f. sp. 'multigermtubi' MB_m1]
          Length=133

 Score = 174.5 bits (441),  Expect = 1E-53
 Identities = 115/119 (96%), Positives = 116/119 (97%), Gaps = 0/119 (0%)
```

```
Query  16   NAQSRSSKAGLAFPVGRVHRLLRKGNYAQRVGAGAPXXXXXXXXXXXXXXXXXXGNAARD  75
            NAQSRSSKAGLAFPVGRVHRLLRKGNYAQRVGAGAPVYLAAVLEYLAAEILELAGNAARD
Sbjct  15   NAQSRSSKAGLAFPVGRVHRLLRKGNYAQRVGAGAPVYLAAVLEYLAAEILELAGNAARD  74

Query  76   NKKTRIIPRHLQLAIRNDEELNKLLGHVTIAQGGVLPNIHQNLLPXXXXXXXXXSSMEL  134
            NKKTRIIPRHLQLAIRNDEELNKLLGHVTIAQGGVLPNIHQNLLPKKT K GKT+S EL
Sbjct  75   NKKTRIIPRHLQLAIRNDEELNKLLGHVTIAQGGVLPNIHQNLLPKKTVKGGKTASQEL  133


>XP_033461959.1| histone H2A [Dissoconium aciculare CBS 342.82]
 gb|KAF1824923.1| histone H2A [Dissoconium aciculare CBS 342.82]
          Length=134

 Score = 174.5 bits (441),  Expect = 1E-53
 Identities = 111/119 (93%), Positives = 111/119 (93%), Gaps = 0/119 (0%)

Query  16   NAQSRSSKAGLAFPVGRVHRLLRKGNYAQRVGAGAPXXXXXXXXXXXXXXXXXXGNAARD  75
            NAQSRSSKAGLAFPVGRVHRLLRKGNYAQRVGAGAPVYLAAVLEYLAAEILELAGNAARD
Sbjct  16   NAQSRSSKAGLAFPVGRVHRLLRKGNYAQRVGAGAPVYLAAVLEYLAAEILELAGNAARD  75

Query  76   NKKTRIIPRHLQLAIRNDEELNKLLGHVTIAQGGVLPNIHQNLLPXXXXXXXXXSSMEL  134
            NKKTRIIPRHLQLAIRNDEELNKLLGHVTIAQGGVLPNIHQNLLPKKT      SS  L
Sbjct  76   NKKTRIIPRHLQLAIRNDEELNKLLGHVTIAQGGVLPNIHQNLLPKKTSTAKDKSSQNL  134


>XP_003344381.1| putative HTA2 protein [Sordaria macrospora k-hell]
 ref|XP_007912732.1| putative histone h2a protein [Phaeoacremonium minimum UCRPA7]
 ref|XP_009853330.1| histone H2A [Neurospora tetrasperma FGSC 2508]
 ref|XP_018244168.1| histone H2A [Fusarium oxysporum f. sp. lycopersici 4287]
 ref|XP_018749950.1| histone H2A [Fusarium verticillioides 7600]
 ref|XP_023429527.1| H2A histone H2A [Fusarium fujikuroi IMI 58289]
 ref|XP_031020765.1| uncharacterized protein FIESC28_01023 [Fusarium coffeatum]
 ref|XP_031050493.1| histone H2A [Fusarium oxysporum NRRL 32931]
```

ref|XP_031068348.1| histone H2A [Fusarium odoratissimum NRRL 54006]
ref|XP_031079033.1| Histone H2A [Fusarium proliferatum ET1]
ref|XP_959442.1| histone H2A [Neurospora crassa OR74A]
sp|Q8X132.3| RecName: Full=Histone H2A [Neurospora crassa OR74A]
gb|AAL38970.1| histone H2A [Neurospora crassa]
gb|EGU78637.1| hypothetical protein FOXB_10823 [Fusarium oxysporum Fo5176]
gb|EGZ69242.1| histone H2A [Neurospora tetrasperma FGSC 2509]
gb|EMT68716.1| Histone H2A [Fusarium odoratissimum]
gb|ENH68241.1| Histone H2A [Fusarium oxysporum f. sp. cubense race 1]
gb|EWZ47615.1| histone H2A [Fusarium oxysporum Fo47]
gb|EWZ92666.1| histone H2A [Fusarium oxysporum f. sp. lycopersici MN25]
gb|EXA42796.1| histone H2A [Fusarium oxysporum f. sp. pisi HDV247]
gb|EXK44944.1| histone H2A [Fusarium oxysporum f. sp. melonis 26406]
gb|EXK97790.1| histone H2A [Fusarium oxysporum f. sp. raphani 54005]
gb|EXL52944.1| histone H2A [Fusarium oxysporum f. sp. radicis-lycopersici 26381]
gb|EXL67236.1| histone H2A [Fusarium oxysporum f. sp. conglutinans race 2 54008]
gb|EXM25571.1| histone H2A [Fusarium oxysporum f. sp. vasinfectum 25433]
gb|KAA8630801.1| hypothetical protein SMACR_08324 [Sordaria macrospora]
gb|KAF4345798.1| histone H2A [Fusarium beomiforme]
gb|KAF4438433.1| Histone H2A [Fusarium acutatum]
gb|KAF4453530.1| histone H2A [Fusarium austroafricanum]
gb|KAF4463734.1| Histone H2A [Fusarium albosuccineum]
gb|KAF4497660.1| Histone H2A [Fusarium agapanthi]
gb|KAF4944350.1| hypothetical protein FSARC_14698 [Fusarium sarcochroum]
gb|KAF4947323.1| hypothetical protein FGADI_10504 [Fusarium gaditjirri]
gb|KAF4982867.1| hypothetical protein FDECE_17425 [Fusarium decemcellulare]
gb|KAF4992062.1| hypothetical protein FGRMN_7413 [Fusarium heterosporum]
gb|KAF5019009.1| hypothetical protein F66182_8983 [Fusarium sp. NRRL 66182]
gb|KAF5244485.1| hypothetical protein FANTH_7754 [Fusarium anthophilum]
gb|KAF5268059.1| hypothetical protein FOXYS1_1054 [Fusarium oxysporum]
gb|KAF5530854.1| histone H2A [Fusarium napiforme]
gb|KAF5535148.1| histone H2A [Fusarium phyllophilum]
gb|KAF5546170.1| histone H2A [Fusarium mexicanum]
gb|KIL91835.1| histone h2a [Fusarium avenaceum]

```
 gb|KLO78778.1| H2A histone H2A [Fusarium fujikuroi]
 gb|PCD41936.1| hypothetical protein AU210_004477 [Fusarium oxysporum f. sp. radicis-
cucumerinum]
 gb|PNP84345.1| hypothetical protein FNYG_01974 [Fusarium nygamai]
 gb|RBA15007.1| histone H2A [Fusarium proliferatum]
 gb|RBQ69682.1| hypothetical protein FVER14953_05100 [Fusarium verticillioides]
 gb|RFN46440.1| histone h2a [Fusarium fasciculatum]
 gb|RGP60347.1| histone h2a [Fusarium longipes]
 gb|RKK25550.1| hypothetical protein BFJ65_g3457 [Fusarium oxysporum f. sp. cepae]
 gb|RYC81222.1| hypothetical protein BFJ63_vAg15893 [Fusarium oxysporum f. sp. narcissi]
 gb|TVY76913.1| Histone H2A [Fusarium oxysporum f. sp. cubense]
 emb|CVK86639.1| Histone H2A [Fusarium mangiferae]
         Length=134

 Score = 174.1 bits (440),  Expect = 2E-53
 Identities = 116/119 (97%), Positives = 117/119 (98%), Gaps = 0/119 (0%)

Query  16   NAQSRSSKAGLAFPVGRVHRLLRKGNYAQRVGAGAPXXXXXXXXXXXXXXXXXXGNAARD  75
            NAQSRSSKAGLAFPVGRVHRLLRKGNYAQRVGAGAPVYLAAVLEYLAAEILELAGNAARD
Sbjct  16   NAQSRSSKAGLAFPVGRVHRLLRKGNYAQRVGAGAPVYLAAVLEYLAAEILELAGNAARD  75

Query  76   NKKTRIIPRHLQLAIRNDEELNKLLGHVTIAQGGVLPNIHQNLLPXXXXXXXXXSSMEL  134
            NKKTRIIPRHLQLAIRNDEELNKLLGHVTIAQGGVLPNIHQNLLPKKTGKTGK +S EL
Sbjct  76   NKKTRIIPRHLQLAIRNDEELNKLLGHVTIAQGGVLPNIHQNLLPKKTGKTGKNASQEL  134


>XP_003051923.1| histone 2A [Fusarium vanettenii 77-13-4]
 gb|EEU46210.1| histone 2A [Fusarium vanettenii 77-13-4]
 gb|RSL46972.1| Histone H2A [Fusarium sp. AF-6]
         Length=134

 Score = 173.7 bits (439),  Expect = 2E-53
 Identities = 114/119 (95%), Positives = 115/119 (97%), Gaps = 0/119 (0%)
```

```
Query  16   NAQSRSSKAGLAFPVGRVHRLLRKGNYAQRVGAGAPXXXXXXXXXXXXXXXXXXGNAARD  75
            NAQSRSSKAGLAFPVGRVHRLLRKGNYAQRVGAGAPVYLAAVLEYLAAEILELAGNAARD
Sbjct  16   NAQSRSSKAGLAFPVGRVHRLLRKGNYAQRVGAGAPVYLAAVLEYLAAEILELAGNAARD  75

Query  76   NKKTRIIPRHLQLAIRNDEELNKLLGHVTIAQGGVLPNIHQNLLPXXXXXXXXXSSMEL  134
            NKKTRIIPRHLQLAIRNDEELNKLLGHVTIAQGGVLPNIHQNLLPKKT K GK +S EL
Sbjct  76   NKKTRIIPRHLQLAIRNDEELNKLLGHVTIAQGGVLPNIHQNLLPKKTVKGGKNASQEL  134


>XP_018184446.1| histone-fold-containing protein [Xylona heveae TC161]
 gb|KZF18891.1| histone-fold-containing protein [Xylona heveae TC161]
         Length=135

 Score = 173.7 bits (439),  Expect = 3E-53
 Identities = 114/119 (95%), Positives = 115/119 (97%), Gaps = 0/119 (0%)

Query  16   NAQSRSSKAGLAFPVGRVHRLLRKGNYAQRVGAGAPXXXXXXXXXXXXXXXXXXGNAARD  75
            NAQSRSSKAGLAFPVGRVHRLLRKGNYAQRVGAGAPVYLAAVLEYLAAEILELAGNAARD
Sbjct  17   NAQSRSSKAGLAFPVGRVHRLLRKGNYAQRVGAGAPVYLAAVLEYLAAEILELAGNAARD  76

Query  76   NKKTRIIPRHLQLAIRNDEELNKLLGHVTIAQGGVLPNIHQNLLPXXXXXXXXXSSMEL  134
            NKKTRIIPRHLQLAIRNDEELNKLLGHVTIAQGGVLPNIHQNLLPKKT K GK +S EL
Sbjct  77   NKKTRIIPRHLQLAIRNDEELNKLLGHVTIAQGGVLPNIHQNLLPKKTSKPGKPASQEL  135


>XP_022475722.1| histone H2A [Colletotrichum orchidophilum]
 ref|XP_031877650.1| uncharacterized protein CGMCC3_g15738 [Colletotrichum fructicola]
 ref|XP_035326357.1| histone h2a [Colletotrichum scovillei]
 gb|EQB54347.1| histone H2A [Colletotrichum gloeosporioides Cg-14]
 gb|EXF85825.1| histone H2A [Colletotrichum fioriniae PJ7]
 gb|KAF0314969.1| histone h2a [Colletotrichum asianum]
 gb|KAF3796989.1| Histone H2A [Colletotrichum gloeosporioides]
 gb|KAF4477738.1| Histone H2A [Colletotrichum fructicola Nara gc5]
 gb|KAF4811425.1| Histone H2A [Colletotrichum tropicale]
```

```
 gb|KAF4823799.1| Histone H2A [Colletotrichum siamense]
 gb|KAF4898627.1| Histone H2A [Colletotrichum viniferum]
 gb|KAF5517402.1| Histone H2A [Colletotrichum aenigma]
 gb|KXH26699.1| histone H2A [Colletotrichum simmondsii]
 gb|KXH39228.1| histone H2A [Colletotrichum nymphaeae SA-01]
 gb|KXH54829.1| histone H2A [Colletotrichum salicis]
 gb|KZL75419.1| Histone H2A [Colletotrichum tofieldiae]
 gb|OHX00217.1| histone h2a [Colletotrichum incanum]
 gb|OLN87736.1| Histone H2A [Colletotrichum chlorophyti]
 gb|TDZ19758.1| Histone H2A [Colletotrichum orbiculare MAFF 240422]
 gb|TDZ32558.1| Histone H2A [Colletotrichum spinosum]
 gb|TDZ34069.1| Histone H2A [Colletotrichum trifolii]
          Length=134

 Score = 173.7 bits (439),  Expect = 3E-53
 Identities = 116/119 (97%), Positives = 116/119 (97%), Gaps = 0/119 (0%)

Query  16   NAQSRSSKAGLAFPVGRVHRLLRKGNYAQRVGAGAPXXXXXXXXXXXXXXXXXXGNAARD  75
            NAQSRSSKAGLAFPVGRVHRLLRKGNYAQRVGAGAPVYLAAVLEYLAAEILELAGNAARD
Sbjct  16   NAQSRSSKAGLAFPVGRVHRLLRKGNYAQRVGAGAPVYLAAVLEYLAAEILELAGNAARD  75

Query  76   NKKTRIIPRHLQLAIRNDEELNKLLGHVTIAQGGVLPNIHQNLLPXXXXXXXXXXSSMEL  134
            NKKTRIIPRHLQLAIRNDEELNKLLGHVTIAQGGVLPNIHQNLLPKKTGKTGK  S EL
Sbjct  76   NKKTRIIPRHLQLAIRNDEELNKLLGHVTIAQGGVLPNIHQNLLPKKTGKTGKNQSQEL  134


>XP_008100773.1| histone H2A [Colletotrichum graminicola M1.001]
 gb|EFQ36753.1| histone H2A [Colletotrichum graminicola M1.001]
          Length=134

 Score = 173.7 bits (439),  Expect = 3E-53
 Identities = 115/119 (96%), Positives = 116/119 (97%), Gaps = 0/119 (0%)

Query  16   NAQSRSSKAGLAFPVGRVHRLLRKGNYAQRVGAGAPXXXXXXXXXXXXXXXXXXGNAARD  75
```

```
           NAQSRSSKAGLAFPVGRVHRLLRKGNYAQRVGAGAPVYLAAVLEYLAAEILELAGNAARD
Sbjct  16   NAQSRSSKAGLAFPVGRVHRLLRKGNYAQRVGAGAPVYLAAVLEYLAAEILELAGNAARD  75

Query  76   NKKTRIIPRHLQLAIRNDEELNKLLGHVTIAQGGVLPNIHQNLLPXXXXXXXXXSSMEL  134
            NKKTRIIPRHLQLAIRNDEELNKLLGHVTIAQGGVLPNIHQNLLPKKTGK+GK  S EL
Sbjct  76   NKKTRIIPRHLQLAIRNDEELNKLLGHVTIAQGGVLPNIHQNLLPKKTGKSGKNQSQEL  134


>XP_002999953.1| histone H2A [Verticillium alfalfae VaMs.102]
 ref|XP_009656670.1| histone H2A [Verticillium dahliae VdLs.17]
 gb|KAF3351846.1| Indoleamine 2,3-dioxygenase family protein [Verticillium dahliae VDG2]
 gb|KAF3352213.1| Putative DNA repair helicase ercc3 [Verticillium dahliae VDG1]
 gb|PNH26664.1| hypothetical protein BJF96_g10051 [Verticillium dahliae]
 emb|CRJ79961.1| hypothetical protein BN1708_000131 [Verticillium longisporum]
 gb|EEY23563.1| histone H2A [Verticillium alfalfae VaMs.102]
          Length=134

 Score = 173.7 bits (439),  Expect = 3E-53
 Identities = 115/119 (96%), Positives = 116/119 (97%), Gaps = 0/119 (0%)

Query  16   NAQSRSSKAGLAFPVGRVHRLLRKGNYAQRVGAGAPXXXXXXXXXXXXXXXXXXGNAARD  75
            NAQSRSSKAGLAFPVGRVHRLLRKGNYAQRVGAGAPVYLAAVLEYLAAEILELAGNAARD
Sbjct  16   NAQSRSSKAGLAFPVGRVHRLLRKGNYAQRVGAGAPVYLAAVLEYLAAEILELAGNAARD  75

Query  76   NKKTRIIPRHLQLAIRNDEELNKLLGHVTIAQGGVLPNIHQNLLPXXXXXXXXXSSMEL  134
            NKKTRIIPRHLQLAIRNDEELNKLLGHVTIAQGGVLPNIHQNLLPKK+GKTGK  S EL
Sbjct  76   NKKTRIIPRHLQLAIRNDEELNKLLGHVTIAQGGVLPNIHQNLLPKKSGKTGKNQSQEL  134


>XP_002486399.1| histone H2A [Talaromyces stipitatus ATCC 10500]
 gb|EED14161.1| histone H2A [Talaromyces stipitatus ATCC 10500]
          Length=133

 Score = 172.9 bits (437),  Expect = 4E-53
```

```
 Identities = 114/119 (95%), Positives = 115/119 (97%), Gaps = 0/119 (0%)

Query  16   NAQSRSSKAGLAFPVGRVHRLLRKGNYAQRVGAGAPXXXXXXXXXXXXXXXXXXGNAARD  75
            NAQSRSSKAGLAFPVGRVHRLLRKGNYAQRVGAGAPVYLAAVLEYLAAEILELAGNAARD
Sbjct  15   NAQSRSSKAGLAFPVGRVHRLLRKGNYAQRVGAGAPVYLAAVLEYLAAEILELAGNAARD  74

Query  76   NKKTRIIPRHLQLAIRNDEELNKLLGHVTIAQGGVLPNIHQNLLPXXXXXXXXXSSMEL  134
            NKKTRIIPRHLQLAIRNDEELNKLLGHVTIAQGGVLPNIHQNLLPKKT K+GK  S EL
Sbjct  75   NKKTRIIPRHLQLAIRNDEELNKLLGHVTIAQGGVLPNIHQNLLPKKTPKSGKGQSQEL  133


>XP_022582571.1| hypothetical protein ASPZODRAFT_129982 [Penicilliopsis zonata CBS 506.65]
 gb|OJJ48061.1| hypothetical protein ASPZODRAFT_129982 [Penicilliopsis zonata CBS 506.65]
 gb|TQB69902.1| histone H2A [Monascus purpureus]
          Length=133

 Score = 172.6 bits (436),  Expect = 6E-53
 Identities = 114/119 (95%), Positives = 115/119 (97%), Gaps = 0/119 (0%)

Query  16   NAQSRSSKAGLAFPVGRVHRLLRKGNYAQRVGAGAPXXXXXXXXXXXXXXXXXXGNAARD  75
            NAQSRSSKAGLAFPVGRVHRLLRKGNYAQRVGAGAPVYLAAVLEYLAAEILELAGNAARD
Sbjct  15   NAQSRSSKAGLAFPVGRVHRLLRKGNYAQRVGAGAPVYLAAVLEYLAAEILELAGNAARD  74

Query  76   NKKTRIIPRHLQLAIRNDEELNKLLGHVTIAQGGVLPNIHQNLLPXXXXXXXXXSSMEL  134
            NKKTRIIPRHLQLAIRNDEELNKLLGHVTIAQGGVLPNIHQNLLPKKT K+GK  S EL
Sbjct  75   NKKTRIIPRHLQLAIRNDEELNKLLGHVTIAQGGVLPNIHQNLLPKKTPKSGKGPSQEL  133


>XP_003654109.1| histone H2A-like protein [Thermothielavioides terrestris NRRL 8126]
 ref|XP_003665420.1| histone H2A-like protein [Thermothelomyces thermophilus ATCC 42464]
 gb|KXX74669.1| Histone H2A [Madurella mycetomatis]
 emb|SPQ25900.1| 6cf58e13-b2dc-420f-8952-7c338b196cf2 [Thermothielavioides terrestris]
 gb|AEO60175.1| histone H2A-like protein [Thermothelomyces thermophilus ATCC 42464]
 gb|AEO67773.1| histone H2A-like protein [Thermothielavioides terrestris NRRL 8126]
```

```
 gb|KXX74733.1| Histone H2A [Madurella mycetomatis]
          Length=134

 Score = 172.6 bits (436),  Expect = 6E-53
 Identities = 116/119 (97%), Positives = 116/119 (97%), Gaps = 0/119 (0%)

Query  16   NAQSRSSKAGLAFPVGRVHRLLRKGNYAQRVGAGAPXXXXXXXXXXXXXXXXXXGNAARD  75
            NAQSRSSKAGLAFPVGRVHRLLRKGNYAQRVGAGAPVYLAAVLEYLAAEILELAGNAARD
Sbjct  16   NAQSRSSKAGLAFPVGRVHRLLRKGNYAQRVGAGAPVYLAAVLEYLAAEILELAGNAARD  75

Query  76   NKKTRIIPRHLQLAIRNDEELNKLLGHVTIAQGGVLPNIHQNLLPXXXXXXXXXSSMEL  134
            NKKTRIIPRHLQLAIRNDEELNKLLGHVTIAQGGVLPNIHQNLLPKKTGKTGK  S EL
Sbjct  76   NKKTRIIPRHLQLAIRNDEELNKLLGHVTIAQGGVLPNIHQNLLPKKTGKTGKNLSQEL  134


>XP_018181861.1| histone H2A [Purpureocillium lilacinum]
 gb|OAQ79104.1| histone H2A [Purpureocillium lilacinum]
 gb|OAQ93142.1| histone H2A [Purpureocillium lilacinum]
 gb|PWI72587.1| histone H2A [Purpureocillium lilacinum]
          Length=134

 Score = 172.6 bits (436),  Expect = 6E-53
 Identities = 115/119 (96%), Positives = 116/119 (97%), Gaps = 0/119 (0%)

Query  16   NAQSRSSKAGLAFPVGRVHRLLRKGNYAQRVGAGAPXXXXXXXXXXXXXXXXXXGNAARD  75
            NAQSRSSKAGLAFPVGRVHRLLRKGNYAQRVGAGAPVYLAAVLEYLAAEILELAGNAARD
Sbjct  16   NAQSRSSKAGLAFPVGRVHRLLRKGNYAQRVGAGAPVYLAAVLEYLAAEILELAGNAARD  75

Query  76   NKKTRIIPRHLQLAIRNDEELNKLLGHVTIAQGGVLPNIHQNLLPXXXXXXXXXSSMEL  134
            NKKTRIIPRHLQLAIRNDEELNKLLGHVTIAQGGVLPNIHQNLLPKKTGK+GK  S EL
Sbjct  76   NKKTRIIPRHLQLAIRNDEELNKLLGHVTIAQGGVLPNIHQNLLPKKTGKSGKGPSQEL  134


>XP_013332342.1| Histone H2A [Rasamsonia emersonii CBS 393.64]
```

```
 ref|XP_028488459.1| histone-fold-containing protein [Byssochlamys spectabilis]
 gb|OXV06061.1| hypothetical protein Egran_06171 [Elaphomyces granulatus]
 dbj|GAD99849.1| histone H2A [Byssochlamys spectabilis No. 5]
 gb|KKA25730.1| Histone H2A [Rasamsonia emersonii CBS 393.64]
 gb|RWQ98814.1| histone-fold-containing protein [Byssochlamys spectabilis]
          Length=133

 Score = 172.6 bits (436),  Expect = 6E-53
 Identities = 114/119 (95%), Positives = 115/119 (97%), Gaps = 0/119 (0%)

Query  16   NAQSRSSKAGLAFPVGRVHRLLRKGNYAQRVGAGAPXXXXXXXXXXXXXXXXXXGNAARD  75
            NAQSRSSKAGLAFPVGRVHRLLRKGNYAQRVGAGAPVYLAAVLEYLAAEILELAGNAARD
Sbjct  15   NAQSRSSKAGLAFPVGRVHRLLRKGNYAQRVGAGAPVYLAAVLEYLAAEILELAGNAARD  74

Query  76   NKKTRIIPRHLQLAIRNDEELNKLLGHVTIAQGGVLPNIHQNLLPXXXXXXXXXSSMEL  134
            NKKTRIIPRHLQLAIRNDEELNKLLGHVTIAQGGVLPNIHQNLLPKKT K+GK  S EL
Sbjct  75   NKKTRIIPRHLQLAIRNDEELNKLLGHVTIAQGGVLPNIHQNLLPKKTPKSGKNPSQEL  133


>XP_033532232.1| histone 2A [Eremomyces bilateralis CBS 781.70]
 gb|KAF1810601.1| histone 2A [Eremomyces bilateralis CBS 781.70]
          Length=134

 Score = 172.6 bits (436),  Expect = 7E-53
 Identities = 112/119 (94%), Positives = 115/119 (97%), Gaps = 0/119 (0%)

Query  16   NAQSRSSKAGLAFPVGRVHRLLRKGNYAQRVGAGAPXXXXXXXXXXXXXXXXXXGNAARD  75
             AQSRSSKAGLAFPVGRVHRLLRKGNYAQRVGAGAPVYLAAVLEYLAAEILELAGNAARD
Sbjct  16   TAQSRSSKAGLAFPVGRVHRLLRKGNYAQRVGAGAPVYLAAVLEYLAAEILELAGNAARD  75

Query  76   NKKTRIIPRHLQLAIRNDEELNKLLGHVTIAQGGVLPNIHQNLLPXXXXXXXXXSSMEL  134
            NKKTRIIPRHLQLAIRNDEELNKLLGHVTIAQGGVLPNIHQNLLPKKT K GK +S+E+
Sbjct  76   NKKTRIIPRHLQLAIRNDEELNKLLGHVTIAQGGVLPNIHQNLLPKKTAKGGKGASLEM  134
```

```
>XP_031005213.1| Histone H2A [Lachnellula hyalina]
 gb|TVY26425.1| Histone H2A [Lachnellula hyalina]
 gb|TVY36020.1| Histone H2A [Lachnellula subtilissima]
 gb|TVY59223.1| Histone H2A [Lachnellula suecica]
          Length=133

 Score = 172.6 bits (436),  Expect = 7E-53
 Identities = 114/119 (95%), Positives = 114/119 (96%), Gaps = 0/119 (0%)

Query  16   NAQSRSSKAGLAFPVGRVHRLLRKGNYAQRVGAGAPXXXXXXXXXXXXXXXXXXGNAARD  75
            NAQSRSSKAGLAFPVGRVHRLLRKGNYAQRVGAGAPVYLAAVLEYLAAEILELAGNAARD
Sbjct  15   NAQSRSSKAGLAFPVGRVHRLLRKGNYAQRVGAGAPVYLAAVLEYLAAEILELAGNAARD  74

Query  76   NKKTRIIPRHLQLAIRNDEELNKLLGHVTIAQGGVLPNIHQNLLPXXXXXXXXXSSMEL  134
            NKKTRIIPRHLQLAIRNDEELNKLLGHVTIAQGGVLPNIHQNLLPKKT K GK  S EL
Sbjct  75   NKKTRIIPRHLQLAIRNDEELNKLLGHVTIAQGGVLPNIHQNLLPKKTAKGGKGPSQEL  133


>XP_001550543.1| hypothetical protein BCIN_02g06800 [Botrytis cinerea B05.10]
 sp|O74268.4| RecName: Full=Histone H2A [Botrytis cinerea B05.10]
 gb|EMR82204.1| putative histone h2a protein [Botrytis cinerea BcDW1]
 gb|TGO18747.1| hypothetical protein BTUL_0008g01320 [Botrytis tulipae]
 gb|TGO20501.1| hypothetical protein BPAE_0290g00080 [Botrytis paeoniae]
 gb|TGO41766.1| hypothetical protein BHYA_0016g00030 [Botrytis hyacinthi]
 gb|TGO51322.1| hypothetical protein BCON_0163g00030 [Botryotinia convoluta]
 gb|TGO75333.1| hypothetical protein BELL_0220g00170 [Botrytis elliptica]
 gb|THV48699.1| hypothetical protein BGAL_0232g00030 [Botrytis galanthina]
 emb|CCD55848.1| hypothetical protein BofuT4_P154100.1 [Botrytis cinerea T4]
          Length=136

 Score = 172.6 bits (436),  Expect = 8E-53
 Identities = 105/105 (100%), Positives = 105/105 (100%), Gaps = 0/105 (0%)
```

```
Query  16   NAQSRSSKAGLAFPVGRVHRLLRKGNYAQRVGAGAPXXXXXXXXXXXXXXXXXXGNAARD  75
            NAQSRSSKAGLAFPVGRVHRLLRKGNYAQRVGAGAPVYLAAVLEYLAAEILELAGNAARD
Sbjct  16   NAQSRSSKAGLAFPVGRVHRLLRKGNYAQRVGAGAPVYLAAVLEYLAAEILELAGNAARD  75

Query  76   NKKTRIIPRHLQLAIRNDEELNKLLGHVTIAQGGVLPNIHQNLLP  120
            NKKTRIIPRHLQLAIRNDEELNKLLGHVTIAQGGVLPNIHQNLLP
Sbjct  76   NKKTRIIPRHLQLAIRNDEELNKLLGHVTIAQGGVLPNIHQNLLP  120


>XP_001263909.1| histone h2a [Aspergillus fischeri NRRL 181]
 ref|XP_001270560.1| histone h2a [Aspergillus clavatus NRRL 1]
 ref|XP_001824390.1| unnamed protein product [Aspergillus oryzae RIB40]
 ref|XP_002384273.1| histone H2A [Aspergillus flavus NRRL3357]
 ref|XP_015408926.1| histone H2A [Aspergillus nomiae NRRL 13137]
 ref|XP_022383485.1| histone H2A [Aspergillus bombycis]
 ref|XP_026613516.1| histone H2A [Aspergillus thermomutatus]
 ref|XP_031903547.1| Histone H2A [Aspergillus alliaceus]
 ref|XP_031926047.1| Histone H2A [Aspergillus caelatus]
 ref|XP_031936651.1| Histone H2A [Aspergillus pseudonomius]
 ref|XP_033418874.1| histone h2a [Aspergillus lentulus]
 sp|A1CJ10.1| RecName: Full=Histone H2A [Aspergillus clavatus NRRL 1]
 sp|A1D8G8.1| RecName: Full=Histone H2A [Aspergillus fischeri NRRL 181]
 sp|Q2U5A8.3| RecName: Full=Histone H2A [Aspergillus oryzae RIB40]
 sp|Q4WWC6.3| RecName: Full=Histone H2A [Aspergillus fumigatus Af293]
 gb|EIT77930.1| histone 2A [Aspergillus oryzae 3.042]
 gb|KAB8076682.1| Histone H2A [Aspergillus leporis]
 gb|KAB8199338.1| Histone H2A [Aspergillus parasiticus]
 gb|KAB8215182.1| Histone H2A [Aspergillus novoparasiticus]
 gb|KAB8245264.1| Histone H2A [Aspergillus flavus]
 gb|KAB8269660.1| Histone H2A [Aspergillus minisclerotigenes]
 gb|KAE8131144.1| Histone H2A [Aspergillus pseudotamarii]
 gb|KAE8148827.1| Histone H2A [Aspergillus avenaceus]
 gb|KAE8166177.1| Histone H2A [Aspergillus tamarii]
 gb|KAE8308699.1| Histone H2A [Aspergillus transmontanensis]
```

```
 gb|KAE8321468.1| Histone H2A [Aspergillus sergii]
 gb|KAE8336526.1| Histone H2A [Aspergillus arachidicola]
 gb|KAE8349067.1| Histone H2A [Aspergillus coremiiformis]
 gb|KAE8375332.1| Histone H2A [Aspergillus bertholletius]
 gb|KAE8410988.1| Histone H2A [Aspergillus pseudocaelatus]
 gb|KAF4222556.1| hypothetical protein CNMCM5878_003594 [Aspergillus fumigatiaffinis]
 gb|KAF4257174.1| hypothetical protein CNMCM8714_003084 [Aspergillus fumigatus]
 gb|KDE75807.1| histone 2A [Aspergillus oryzae 100-8]
 gb|KJK67648.1| Core histone H2A/H2B/H3/H4 [Aspergillus parasiticus SU-1]
 gb|KMK60799.1| histone h2a [Aspergillus fumigatus Z5]
 gb|KOC17400.1| histone h2a [Aspergillus flavus AF70]
 gb|OJJ34308.1| hypothetical protein ASPWEDRAFT_42278 [Aspergillus wentii DTO 134E9]
 gb|OOO08899.1| Histone core domain protein [Aspergillus oryzae]
 gb|RHZ52186.1| histone H2A [Aspergillus turcosus]
 gb|RJE19382.1| hypothetical protein PHISCL_08278 [Aspergillus sclerotialis]
 gb|RMJ23429.1| hypothetical protein PHISP_05709 [Aspergillus sp. HF37]
 dbj|GAO85727.1| histone H2A [Aspergillus udagawae]
          Length=133

 Score = 172.2 bits (435),  Expect = 9E-53
 Identities = 114/119 (95%), Positives = 115/119 (97%), Gaps = 0/119 (0%)

Query  16   NAQSRSSKAGLAFPVGRVHRLLRKGNYAQRVGAGAPXXXXXXXXXXXXXXXXXXGNAARD  75
            NAQSRSSKAGLAFPVGRVHRLLRKGNYAQRVGAGAPVYLAAVLEYLAAEILELAGNAARD
Sbjct  15   NAQSRSSKAGLAFPVGRVHRLLRKGNYAQRVGAGAPVYLAAVLEYLAAEILELAGNAARD  74

Query  76   NKKTRIIPRHLQLAIRNDEELNKLLGHVTIAQGGVLPNIHQNLLPXXXXXXXXXSSMEL  134
            NKKTRIIPRHLQLAIRNDEELNKLLGHVTIAQGGVLPNIHQNLLPKKT K+GK  S EL
Sbjct  75   NKKTRIIPRHLQLAIRNDEELNKLLGHVTIAQGGVLPNIHQNLLPKKTPKSGKGPSQEL  133


>XP_022400783.1| hypothetical protein ASPGLDRAFT_46875 [Aspergillus glaucus CBS 516.65]
 gb|EYE96320.1| histone-fold-containing protein [Aspergillus ruber CBS 135680]
 gb|ODM18315.1| Histone H2A [Aspergillus cristatus]
```

```
 gb|OJJ84085.1| hypothetical protein ASPGLDRAFT_46875 [Aspergillus glaucus CBS 516.65]
          Length=133

 Score = 172.2 bits (435),  Expect = 9E-53
 Identities = 114/119 (95%), Positives = 114/119 (96%), Gaps = 0/119 (0%)

Query  16   NAQSRSSKAGLAFPVGRVHRLLRKGNYAQRVGAGAPXXXXXXXXXXXXXXXXXGNAARD  75
            NAQSRSSKAGLAFPVGRVHRLLRKGNYAQRVGAGAPVYLAAVLEYLAAEILELAGNAARD
Sbjct  15   NAQSRSSKAGLAFPVGRVHRLLRKGNYAQRVGAGAPVYLAAVLEYLAAEILELAGNAARD  74

Query  76   NKKTRIIPRHLQLAIRNDEELNKLLGHVTIAQGGVLPNIHQNLLPXXXXXXXXXSSMEL  134
            NKKTRIIPRHLQLAIRNDEELNKLLGHVTIAQGGVLPNIHQNLLPKKT K GK  S EL
Sbjct  75   NKKTRIIPRHLQLAIRNDEELNKLLGHVTIAQGGVLPNIHQNLLPKKTPKAGKGPSQEL  133


>XP_033400410.1| uncharacterized protein K452DRAFT_306480 [Aplosporella prunicola CBS 121167]
 gb|KAF2144698.1| hypothetical protein K452DRAFT_306480 [Aplosporella prunicola CBS 121167]
          Length=134

 Score = 172.2 bits (435),  Expect = 9E-53
 Identities = 114/119 (95%), Positives = 114/119 (96%), Gaps = 0/119 (0%)

Query  16   NAQSRSSKAGLAFPVGRVHRLLRKGNYAQRVGAGAPXXXXXXXXXXXXXXXXXGNAARD  75
            NAQSRSSKAGLAFPVGRVHRLLRKGNYAQRVGAGAPVYLAAVLEYLAAEILELAGNAARD
Sbjct  16   NAQSRSSKAGLAFPVGRVHRLLRKGNYAQRVGAGAPVYLAAVLEYLAAEILELAGNAARD  75

Query  76   NKKTRIIPRHLQLAIRNDEELNKLLGHVTIAQGGVLPNIHQNLLPXXXXXXXXXSSMEL  134
            NKKTRIIPRHLQLAIRNDEELNKLLGHVTIAQGGVLPNIHQNLLPKKT K GK  S EL
Sbjct  76   NKKTRIIPRHLQLAIRNDEELNKLLGHVTIAQGGVLPNIHQNLLPKKTSKPGKGPSQEL  134


>XP_033557660.1| histone H2A [Macroventuria anomochaeta]
 gb|KAF2257646.1| histone H2A [Didymosphaeria enalia]
 gb|KAF2730269.1| histone H2A [Polyplosphaeria fusca]
```

```
 gb|KAF3005056.1| histone H2A [Curvularia kusanoi]
 gb|KAF3047239.1| histone H2A [Didymella keratinophila]
 gb|KAF3047442.1| histone H2A [Didymella heteroderae]
 gb|KZM23353.1| DNA binding [Ascochyta rabiei]
 gb|OSS46296.1| hypothetical protein B5807_08630 [Epicoccum nigrum]
          Length=134

 Score = 172.2 bits (435),  Expect = 9E-53
 Identities = 114/119 (95%), Positives = 114/119 (96%), Gaps = 0/119 (0%)

Query  16   NAQSRSSKAGLAFPVGRVHRLLRKGNYAQRVGAGAPXXXXXXXXXXXXXXXXXXGNAARD  75
            NAQSRSSKAGLAFPVGRVHRLLRKGNYAQRVGAGAPVYLAAVLEYLAAEILELAGNAARD
Sbjct  16   NAQSRSSKAGLAFPVGRVHRLLRKGNYAQRVGAGAPVYLAAVLEYLAAEILELAGNAARD  75

Query  76   NKKTRIIPRHLQLAIRNDEELNKLLGHVTIAQGGVLPNIHQNLLPXXXXXXXXXSSMEL  134
            NKKTRIIPRHLQLAIRNDEELNKLLGHVTIAQGGVLPNIHQNLLPKKT K GK  S EL
Sbjct  76   NKKTRIIPRHLQLAIRNDEELNKLLGHVTIAQGGVLPNIHQNLLPKKTAKPGKGPSQEL  134


>XP_009158759.1| histone H2A [Exophiala dermatitidis NIH/UT8656]
 gb|EHY58298.1| histone H2A [Exophiala dermatitidis NIH/UT8656]
          Length=131

 Score = 172.2 bits (435),  Expect = 9E-53
 Identities = 105/105 (100%), Positives = 105/105 (100%), Gaps = 0/105 (0%)

Query  16   NAQSRSSKAGLAFPVGRVHRLLRKGNYAQRVGAGAPXXXXXXXXXXXXXXXXXXGNAARD  75
            NAQSRSSKAGLAFPVGRVHRLLRKGNYAQRVGAGAPVYLAAVLEYLAAEILELAGNAARD
Sbjct  15   NAQSRSSKAGLAFPVGRVHRLLRKGNYAQRVGAGAPVYLAAVLEYLAAEILELAGNAARD  74

Query  76   NKKTRIIPRHLQLAIRNDEELNKLLGHVTIAQGGVLPNIHQNLLP  120
            NKKTRIIPRHLQLAIRNDEELNKLLGHVTIAQGGVLPNIHQNLLP
Sbjct  75   NKKTRIIPRHLQLAIRNDEELNKLLGHVTIAQGGVLPNIHQNLLP  119
```

```
>XP_030999825.1| uncharacterized protein E0L32_002623 [Phialemoniopsis curvata]
 gb|TPX18114.1| hypothetical protein E0L32_002623 [Phialemoniopsis curvata]
          Length=134

 Score = 172.2 bits (435),  Expect = 1E-52
 Identities = 114/119 (95%), Positives = 117/119 (98%), Gaps = 0/119 (0%)

Query  16   NAQSRSSKAGLAFPVGRVHRLLRKGNYAQRVGAGAPXXXXXXXXXXXXXXXXXXGNAARD  75
            N+Q+RSSKAGLAFPVGRVHRLLRKGNYAQRVGAGAPVYLAAVLEYLAAEILELAGNAARD
Sbjct  16   NSQTRSSKAGLAFPVGRVHRLLRKGNYAQRVGAGAPVYLAAVLEYLAAEILELAGNAARD  75

Query  76   NKKTRIIPRHLQLAIRNDEELNKLLGHVTIAQGGVLPNIHQNLLPXXXXXXXXXSSMEL  134
            NKKTRIIPRHLQLAIRNDEELNKLLGHVTIAQGGVLPNIHQNLLPKKTGKTGK +S EL
Sbjct  76   NKKTRIIPRHLQLAIRNDEELNKLLGHVTIAQGGVLPNIHQNLLPKKTGKTGKNASQEL  134


>XP_009221324.1| histone H2A [Gaeumannomyces tritici R3-111a-1]
 gb|EJT75324.1| histone H2A [Gaeumannomyces tritici R3-111a-1]
 gb|KLU84600.1| histone H2A [Magnaporthiopsis poae ATCC 64411]
          Length=136

 Score = 172.2 bits (435),  Expect = 1E-52
 Identities = 105/105 (100%), Positives = 105/105 (100%), Gaps = 0/105 (0%)

Query  16   NAQSRSSKAGLAFPVGRVHRLLRKGNYAQRVGAGAPXXXXXXXXXXXXXXXXXXGNAARD  75
            NAQSRSSKAGLAFPVGRVHRLLRKGNYAQRVGAGAPVYLAAVLEYLAAEILELAGNAARD
Sbjct  16   NAQSRSSKAGLAFPVGRVHRLLRKGNYAQRVGAGAPVYLAAVLEYLAAEILELAGNAARD  75

Query  76   NKKTRIIPRHLQLAIRNDEELNKLLGHVTIAQGGVLPNIHQNLLP  120
            NKKTRIIPRHLQLAIRNDEELNKLLGHVTIAQGGVLPNIHQNLLP
Sbjct  76   NKKTRIIPRHLQLAIRNDEELNKLLGHVTIAQGGVLPNIHQNLLP  120
```

```
>XP_006695183.1| hypothetical protein CTHT_0048200 [Chaetomium thermophilum var. thermophilum
DSM 1495]
 gb|EGS19361.1| hypothetical protein CTHT_0048200 [Chaetomium thermophilum var. thermophilum
DSM 1495]
          Length=133

 Score = 171.8 bits (434),  Expect = 1E-52
 Identities = 114/119 (95%), Positives = 115/119 (97%), Gaps = 0/119 (0%)

Query  16   NAQSRSSKAGLAFPVGRVHRLLRKGNYAQRVGAGAPXXXXXXXXXXXXXXXXXXGNAARD  75
            +AQSRSSKAGLAFPVGRVHRLLRKGNYAQRVGAGAPVYLAAVLEYLAAEILELAGNAARD
Sbjct  15   SAQSRSSKAGLAFPVGRVHRLLRKGNYAQRVGAGAPVYLAAVLEYLAAEILELAGNAARD  74

Query  76   NKKTRIIPRHLQLAIRNDEELNKLLGHVTIAQGGVLPNIHQNLLPXXXXXXXXXSSMEL  134
            NKKTRIIPRHLQLAIRNDEELNKLLGHVTIAQGGVLPNIHQNLLPKKT KTGK  S EL
Sbjct  75   NKKTRIIPRHLQLAIRNDEELNKLLGHVTIAQGGVLPNIHQNLLPKKTAKTGKNLSQEL  133


>XP_016582554.1| histone H2A [Sporothrix schenckii 1099-18]
 gb|EPE03688.1| histone h2a [Ophiostoma piceae UAMH 11346]
 gb|ERT02787.1| histone H2A [Sporothrix schenckii ATCC 58251]
 gb|KIH89123.1| histone H2A [Sporothrix brasiliensis 5110]
 gb|KJR79878.1| histone H2A [Sporothrix schenckii 1099-18]
          Length=134

 Score = 171.8 bits (434),  Expect = 1E-52
 Identities = 113/119 (94%), Positives = 116/119 (97%), Gaps = 0/119 (0%)

Query  16   NAQSRSSKAGLAFPVGRVHRLLRKGNYAQRVGAGAPXXXXXXXXXXXXXXXXXXGNAARD  75
            N+Q+RSSKAGLAFPVGRVHRLLRKGNYAQRVGAGAPVYLAAVLEYLAAEILELAGNAARD
Sbjct  16   NSQTRSSKAGLAFPVGRVHRLLRKGNYAQRVGAGAPVYLAAVLEYLAAEILELAGNAARD  75

Query  76   NKKTRIIPRHLQLAIRNDEELNKLLGHVTIAQGGVLPNIHQNLLPXXXXXXXXXSSMEL  134
            NKKTRIIPRHLQLAIRNDEELNKLLGHVTIAQGGVLPNIHQNLLPKKTGK GK +S EL
```

```
Sbjct  76   NKKTRIIPRHLQLAIRNDEELNKLLGHVTIAQGGVLPNIHQNLLPKKTGKAGKNASQEL  134


>XP_018143750.1| histone H2A [Pochonia chlamydosporia 170]
 gb|KDB10832.1| histone H2A [Ustilaginoidea virens]
 gb|KZZ93423.1| histone H2A [Moelleriella libera RCEF 2490]
 gb|RZR66097.1| histone H2A [Pochonia chlamydosporia 123]
 gb|OAQ66663.1| histone H2A [Pochonia chlamydosporia 170]
 dbj|GAO19906.1| hypothetical protein UVI_02058580 [Ustilaginoidea virens]
          Length=135

 Score = 171.8 bits (434),  Expect = 1E-52
 Identities = 105/105 (100%), Positives = 105/105 (100%), Gaps = 0/105 (0%)

Query  16   NAQSRSSKAGLAFPVGRVHRLLRKGNYAQRVGAGAPXXXXXXXXXXXXXXXXXGNAARD  75
            NAQSRSSKAGLAFPVGRVHRLLRKGNYAQRVGAGAPVYLAAVLEYLAAEILELAGNAARD
Sbjct  16   NAQSRSSKAGLAFPVGRVHRLLRKGNYAQRVGAGAPVYLAAVLEYLAAEILELAGNAARD  75

Query  76   NKKTRIIPRHLQLAIRNDEELNKLLGHVTIAQGGVLPNIHQNLLP  120
            NKKTRIIPRHLQLAIRNDEELNKLLGHVTIAQGGVLPNIHQNLLP
Sbjct  76   NKKTRIIPRHLQLAIRNDEELNKLLGHVTIAQGGVLPNIHQNLLP  120


>XP_006964898.1| histone H2A [Trichoderma reesei QM6a]
 ref|XP_024745810.1| histone-fold-containing protein [Trichoderma citrinoviride]
 gb|ETS02672.1| histone H2A [Trichoderma reesei RUT C-30]
 gb|OTA03406.1| histone H2A [Trichoderma parareesei]
 gb|PTB77158.1| histone-fold-containing protein [Trichoderma longibrachiatum ATCC 18648]
 gb|EGR48861.1| histone H2A [Trichoderma reesei QM6a]
 gb|PTB62490.1| histone-fold-containing protein [Trichoderma citrinoviride]
          Length=135

 Score = 171.8 bits (434),  Expect = 1E-52
 Identities = 105/105 (100%), Positives = 105/105 (100%), Gaps = 0/105 (0%)
```

```
Query  16   NAQSRSSKAGLAFPVGRVHRLLRKGNYAQRVGAGAPXXXXXXXXXXXXXXXXXXGNAARD  75
            NAQSRSSKAGLAFPVGRVHRLLRKGNYAQRVGAGAPVYLAAVLEYLAAEILELAGNAARD
Sbjct  16   NAQSRSSKAGLAFPVGRVHRLLRKGNYAQRVGAGAPVYLAAVLEYLAAEILELAGNAARD  75

Query  76   NKKTRIIPRHLQLAIRNDEELNKLLGHVTIAQGGVLPNIHQNLLP  120
            NKKTRIIPRHLQLAIRNDEELNKLLGHVTIAQGGVLPNIHQNLLP
Sbjct  76   NKKTRIIPRHLQLAIRNDEELNKLLGHVTIAQGGVLPNIHQNLLP  120


>XP_024717498.1| hypothetical protein M430DRAFT_53732 [Amorphotheca resinae ATCC 22711]
 gb|PSS09200.1| hypothetical protein M430DRAFT_53732 [Amorphotheca resinae ATCC 22711]
          Length=133

 Score = 171.8 bits (434),  Expect = 1E-52
 Identities = 114/119 (95%), Positives = 114/119 (96%), Gaps = 0/119 (0%)

Query  16   NAQSRSSKAGLAFPVGRVHRLLRKGNYAQRVGAGAPXXXXXXXXXXXXXXXXXXGNAARD  75
            NAQSRSSKAGLAFPVGRVHRLLRKGNYAQRVGAGAPVYLAAVLEYLAAEILELAGNAARD
Sbjct  15   NAQSRSSKAGLAFPVGRVHRLLRKGNYAQRVGAGAPVYLAAVLEYLAAEILELAGNAARD  74

Query  76   NKKTRIIPRHLQLAIRNDEELNKLLGHVTIAQGGVLPNIHQNLLPXXXXXXXXXSSMEL  134
            NKKTRIIPRHLQLAIRNDEELNKLLGHVTIAQGGVLPNIHQNLLPKKT K GK  S EL
Sbjct  75   NKKTRIIPRHLQLAIRNDEELNKLLGHVTIAQGGVLPNIHQNLLPKKTPKGGKNPSQEL  133


>XP_016643417.1| hypothetical protein SAPIO_CDS4549 [Scedosporium apiospermum]
 gb|KEZ43618.1| hypothetical protein SAPIO_CDS4549 [Scedosporium apiospermum]
          Length=136

 Score = 171.8 bits (434),  Expect = 1E-52
 Identities = 105/105 (100%), Positives = 105/105 (100%), Gaps = 0/105 (0%)

Query  16   NAQSRSSKAGLAFPVGRVHRLLRKGNYAQRVGAGAPXXXXXXXXXXXXXXXXXXGNAARD  75
```

```
            NAQSRSSKAGLAFPVGRVHRLLRKGNYAQRVGAGAPVYLAAVLEYLAAEILELAGNAARD
Sbjct  16   NAQSRSSKAGLAFPVGRVHRLLRKGNYAQRVGAGAPVYLAAVLEYLAAEILELAGNAARD  75

Query  76   NKKTRIIPRHLQLAIRNDEELNKLLGHVTIAQGGVLPNIHQNLLP  120
            NKKTRIIPRHLQLAIRNDEELNKLLGHVTIAQGGVLPNIHQNLLP
Sbjct  76   NKKTRIIPRHLQLAIRNDEELNKLLGHVTIAQGGVLPNIHQNLLP  120


>XP_013957466.1| hypothetical protein TRIVIDRAFT_215760 [Trichoderma virens Gv29-8]
 gb|EHK23231.1| hypothetical protein TRIVIDRAFT_215760 [Trichoderma virens Gv29-8]
 gb|RFU75506.1| histone h2a [Trichoderma arundinaceum]
          Length=135

 Score = 171.8 bits (434),  Expect = 1E-52
 Identities = 105/105 (100%), Positives = 105/105 (100%), Gaps = 0/105 (0%)

Query  16   NAQSRSSKAGLAFPVGRVHRLLRKGNYAQRVGAGAPXXXXXXXXXXXXXXXXXGNAARD  75
            NAQSRSSKAGLAFPVGRVHRLLRKGNYAQRVGAGAPVYLAAVLEYLAAEILELAGNAARD
Sbjct  16   NAQSRSSKAGLAFPVGRVHRLLRKGNYAQRVGAGAPVYLAAVLEYLAAEILELAGNAARD  75

Query  76   NKKTRIIPRHLQLAIRNDEELNKLLGHVTIAQGGVLPNIHQNLLP  120
            NKKTRIIPRHLQLAIRNDEELNKLLGHVTIAQGGVLPNIHQNLLP
Sbjct  76   NKKTRIIPRHLQLAIRNDEELNKLLGHVTIAQGGVLPNIHQNLLP  120


>XP_008078485.1| Histone-fold containing protein [Glarea lozoyensis ATCC 20868]
 gb|EHL00038.1| putative Histone H2A [Glarea lozoyensis 74030]
 gb|EPE34550.1| Histone-fold containing protein [Glarea lozoyensis ATCC 20868]
          Length=134

 Score = 171.8 bits (434),  Expect = 1E-52
 Identities = 105/105 (100%), Positives = 105/105 (100%), Gaps = 0/105 (0%)

Query  16   NAQSRSSKAGLAFPVGRVHRLLRKGNYAQRVGAGAPXXXXXXXXXXXXXXXXXGNAARD  75
```

```
           NAQSRSSKAGLAFPVGRVHRLLRKGNYAQRVGAGAPVYLAAVLEYLAAEILELAGNAARD
Sbjct  15   NAQSRSSKAGLAFPVGRVHRLLRKGNYAQRVGAGAPVYLAAVLEYLAAEILELAGNAARD  74

Query  76   NKKTRIIPRHLQLAIRNDEELNKLLGHVTIAQGGVLPNIHQNLLP  120
            NKKTRIIPRHLQLAIRNDEELNKLLGHVTIAQGGVLPNIHQNLLP
Sbjct  75   NKKTRIIPRHLQLAIRNDEELNKLLGHVTIAQGGVLPNIHQNLLP  119


>XP_002152696.1| histone H2A [Talaromyces marneffei ATCC 18224]
 gb|KAE8548065.1| hypothetical protein EYB25_009858 [Talaromyces marneffei]
 gb|KAF3396881.1| Histone H2A [Talaromyces pinophilus]
 gb|KFX41866.1| Histone H2A [Talaromyces marneffei PM1]
 gb|KUL83365.1| hypothetical protein ZTR_11232 [Talaromyces verruculosus]
 gb|PCH02279.1| Histone-fold [Penicillium sp. 'occitanis']
 gb|RAO72971.1| hypothetical protein BHQ10_008983 [Talaromyces amestolkiae]
 dbj|GAM43578.1| histone [Talaromyces cellulolyticus]
          Length=133

 Score = 171.8 bits (434),  Expect = 1E-52
 Identities = 113/119 (94%), Positives = 115/119 (97%), Gaps = 0/119 (0%)

Query  16   NAQSRSSKAGLAFPVGRVHRLLRKGNYAQRVGAGAPXXXXXXXXXXXXXXXXXXGNAARD  75
            N+QSRSSKAGLAFPVGRVHRLLRKGNYAQRVGAGAPVYLAAVLEYLAAEILELAGNAARD
Sbjct  15   NSQSRSSKAGLAFPVGRVHRLLRKGNYAQRVGAGAPVYLAAVLEYLAAEILELAGNAARD  74

Query  76   NKKTRIIPRHLQLAIRNDEELNKLLGHVTIAQGGVLPNIHQNLLPXXXXXXXXXSSMEL  134
            NKKTRIIPRHLQLAIRNDEELNKLLGHVTIAQGGVLPNIHQNLLPKKT K+GK  S EL
Sbjct  75   NKKTRIIPRHLQLAIRNDEELNKLLGHVTIAQGGVLPNIHQNLLPKKTPKSGKGQSQEL  133


>XP_024769015.1| hypothetical protein M431DRAFT_154042 [Trichoderma harzianum CBS 226.95]
 gb|KAF3059124.1| Histone H2A [Trichoderma lentiforme]
 gb|KKP02237.1| histone H2A [Trichoderma harzianum]
 gb|OPB36953.1| Histone H2A [Trichoderma guizhouense]
```

```
 gb|PKK51312.1| hypothetical protein CI102_3093 [Trichoderma harzianum]
 gb|PNP45982.1| hypothetical protein THARTR1_10855 [Trichoderma harzianum]
          Length=135

 Score = 171.8 bits (434),  Expect = 1E-52
 Identities = 105/105 (100%), Positives = 105/105 (100%), Gaps = 0/105 (0%)

Query  16   NAQSRSSKAGLAFPVGRVHRLLRKGNYAQRVGAGAPXXXXXXXXXXXXXXXXXXGNAARD  75
            NAQSRSSKAGLAFPVGRVHRLLRKGNYAQRVGAGAPVYLAAVLEYLAAEILELAGNAARD
Sbjct  16   NAQSRSSKAGLAFPVGRVHRLLRKGNYAQRVGAGAPVYLAAVLEYLAAEILELAGNAARD  75

Query  76   NKKTRIIPRHLQLAIRNDEELNKLLGHVTIAQGGVLPNIHQNLLP  120
            NKKTRIIPRHLQLAIRNDEELNKLLGHVTIAQGGVLPNIHQNLLP
Sbjct  76   NKKTRIIPRHLQLAIRNDEELNKLLGHVTIAQGGVLPNIHQNLLP  120


>XP_007806467.1| histone H2A [Metarhizium acridum CQMa 102]
 ref|XP_007820759.1| Histone H2A [Metarhizium robertsii ARSEF 23]
 ref|XP_014547579.1| Histone H2A, partial [Metarhizium brunneum ARSEF 3297]
 gb|EXV06217.1| histone 2A [Metarhizium robertsii]
 gb|KAF5130503.1| Histone H2A [Metarhizium anisopliae]
 gb|KHO01121.1| histone H2A [Metarhizium album ARSEF 1941]
 gb|KID62355.1| Histone H2A, partial [Metarhizium anisopliae ARSEF 549]
 gb|KID92549.1| Histone H2A [Metarhizium guizhouense ARSEF 977]
 gb|KIE01520.1| Histone H2A, partial [Metarhizium majus ARSEF 297]
 gb|KJK84133.1| hypothetical protein H634G_00496 [Metarhizium anisopliae BRIP 53293]
 gb|KJK93949.1| hypothetical protein H633G_02214 [Metarhizium anisopliae BRIP 53284]
          Length=135

 Score = 171.8 bits (434),  Expect = 1E-52
 Identities = 119/120 (99%), Positives = 119/120 (99%), Gaps = 0/120 (0%)

Query  1    MTXXXXXXXXXXXXXXNAQSRSSKAGLAFPVGRVHRLLRKGNYAQRVGAGAPXXXXXXXXX  60
            MTG GKSGGKASGSKNAQSRSSKAGLAFPVGRVHRLLRKGNYAQRVGAGAPVYLAAVLEY
```

```
Sbjct  1    MTGAGKSGGKASGSKNAQSRSSKAGLAFPVGRVHRLLRKGNYAQRVGAGAPVYLAAVLEY  60

Query  61   XXXXXXXXXGNAARDNKKTRIIPRHLQLAIRNDEELNKLLGHVTIAQGGVLPNIHQNLLP  120
            LAAEILELAGNAARDNKKTRIIPRHLQLAIRNDEELNKLLGHVTIAQGGVLPNIHQNLLP
Sbjct  61   LAAEILELAGNAARDNKKTRIIPRHLQLAIRNDEELNKLLGHVTIAQGGVLPNIHQNLLP  120


>XP_003716333.1| histone H2A [Pyricularia oryzae 70-15]
 ref|XP_030986776.1| uncharacterized protein PgNI_00437 [Pyricularia grisea]
 sp|L7HZV6.1| RecName: Full=Histone H2A [Pyricularia oryzae Y34]
 sp|P0CT12.1| RecName: Full=Histone H2A [Pyricularia oryzae 70-15]
 gb|ELQ59706.1| histone H2A [Pyricularia oryzae P131]
 gb|QBZ60545.1| hypothetical protein PoMZ_07487 [Pyricularia oryzae]
 gb|TLD32745.1| hypothetical protein PspLS_01276 [Pyricularia sp. CBS 133598]
 gb|AAW69352.1| histone H2A-like protein [Pyricularia grisea]
 gb|EHA50014.1| histone H2A [Pyricularia oryzae 70-15]
          Length=136

 Score = 171.8 bits (434),  Expect = 1E-52
 Identities = 105/105 (100%), Positives = 105/105 (100%), Gaps = 0/105 (0%)

Query  16   NAQSRSSKAGLAFPVGRVHRLLRKGNYAQRVGAGAPXXXXXXXXXXXXXXXXXXGNAARD  75
            NAQSRSSKAGLAFPVGRVHRLLRKGNYAQRVGAGAPVYLAAVLEYLAAEILELAGNAARD
Sbjct  16   NAQSRSSKAGLAFPVGRVHRLLRKGNYAQRVGAGAPVYLAAVLEYLAAEILELAGNAARD  75

Query  76   NKKTRIIPRHLQLAIRNDEELNKLLGHVTIAQGGVLPNIHQNLLP  120
            NKKTRIIPRHLQLAIRNDEELNKLLGHVTIAQGGVLPNIHQNLLP
Sbjct  76   NKKTRIIPRHLQLAIRNDEELNKLLGHVTIAQGGVLPNIHQNLLP  120


>XP_031866196.1| Histone H2A [Venustampulla echinocandica]
 gb|RDL32474.1| Histone H2A [Venustampulla echinocandica]
          Length=134
```

```
 Score = 171.8 bits (434),  Expect = 2E-52
 Identities = 105/105 (100%), Positives = 105/105 (100%), Gaps = 0/105 (0%)

Query  16   NAQSRSSKAGLAFPVGRVHRLLRKGNYAQRVGAGAPXXXXXXXXXXXXXXXXXXGNAARD  75
            NAQSRSSKAGLAFPVGRVHRLLRKGNYAQRVGAGAPVYLAAVLEYLAAEILELAGNAARD
Sbjct  15   NAQSRSSKAGLAFPVGRVHRLLRKGNYAQRVGAGAPVYLAAVLEYLAAEILELAGNAARD  74

Query  76   NKKTRIIPRHLQLAIRNDEELNKLLGHVTIAQGGVLPNIHQNLLP  120
            NKKTRIIPRHLQLAIRNDEELNKLLGHVTIAQGGVLPNIHQNLLP
Sbjct  75   NKKTRIIPRHLQLAIRNDEELNKLLGHVTIAQGGVLPNIHQNLLP  119


>XP_018153697.1| Histone H2A [Colletotrichum higginsianum IMI 349063]
 gb|OBR05179.1| Histone H2A [Colletotrichum higginsianum IMI 349063]
 gb|TIC94304.1| Histone H2A [Colletotrichum higginsianum]
 emb|CCF43662.1| histone H2A [Colletotrichum higginsianum]
          Length=133

 Score = 171.8 bits (434),  Expect = 2E-52
 Identities = 105/105 (100%), Positives = 105/105 (100%), Gaps = 0/105 (0%)

Query  16   NAQSRSSKAGLAFPVGRVHRLLRKGNYAQRVGAGAPXXXXXXXXXXXXXXXXXXGNAARD  75
            NAQSRSSKAGLAFPVGRVHRLLRKGNYAQRVGAGAPVYLAAVLEYLAAEILELAGNAARD
Sbjct  16   NAQSRSSKAGLAFPVGRVHRLLRKGNYAQRVGAGAPVYLAAVLEYLAAEILELAGNAARD  75

Query  76   NKKTRIIPRHLQLAIRNDEELNKLLGHVTIAQGGVLPNIHQNLLP  120
            NKKTRIIPRHLQLAIRNDEELNKLLGHVTIAQGGVLPNIHQNLLP
Sbjct  76   NKKTRIIPRHLQLAIRNDEELNKLLGHVTIAQGGVLPNIHQNLLP  120


>XP_035320237.1| histone H2A [Geosmithia morbida]
 gb|KAF4121585.1| histone H2A [Geosmithia morbida]
          Length=133
```

```
 Score = 171.4 bits (433),  Expect = 2E-52
 Identities = 105/105 (100%), Positives = 105/105 (100%), Gaps = 0/105 (0%)

Query  16   NAQSRSSKAGLAFPVGRVHRLLRKGNYAQRVGAGAPXXXXXXXXXXXXXXXXXXGNAARD  75
            NAQSRSSKAGLAFPVGRVHRLLRKGNYAQRVGAGAPVYLAAVLEYLAAEILELAGNAARD
Sbjct  16   NAQSRSSKAGLAFPVGRVHRLLRKGNYAQRVGAGAPVYLAAVLEYLAAEILELAGNAARD  75

Query  76   NKKTRIIPRHLQLAIRNDEELNKLLGHVTIAQGGVLPNIHQNLLP  120
            NKKTRIIPRHLQLAIRNDEELNKLLGHVTIAQGGVLPNIHQNLLP
Sbjct  76   NKKTRIIPRHLQLAIRNDEELNKLLGHVTIAQGGVLPNIHQNLLP  120


>XP_001217588.1| histone H2A [Aspergillus terreus NIH2624]
 sp|Q0CBD2.1| RecName: Full=Histone H2A [Aspergillus terreus NIH2624]
 gb|EAU31134.1| histone H2A [Aspergillus terreus NIH2624]
 dbj|GES65593.1| hypothetical protein ATETN484_0012049800 [Aspergillus terreus]
 dbj|GFF19736.1| histone H2A [Aspergillus terreus]
          Length=131

 Score = 171.4 bits (433),  Expect = 2E-52
 Identities = 105/105 (100%), Positives = 105/105 (100%), Gaps = 0/105 (0%)

Query  16   NAQSRSSKAGLAFPVGRVHRLLRKGNYAQRVGAGAPXXXXXXXXXXXXXXXXXXGNAARD  75
            NAQSRSSKAGLAFPVGRVHRLLRKGNYAQRVGAGAPVYLAAVLEYLAAEILELAGNAARD
Sbjct  15   NAQSRSSKAGLAFPVGRVHRLLRKGNYAQRVGAGAPVYLAAVLEYLAAEILELAGNAARD  74

Query  76   NKKTRIIPRHLQLAIRNDEELNKLLGHVTIAQGGVLPNIHQNLLP  120
            NKKTRIIPRHLQLAIRNDEELNKLLGHVTIAQGGVLPNIHQNLLP
Sbjct  75   NKKTRIIPRHLQLAIRNDEELNKLLGHVTIAQGGVLPNIHQNLLP  119


>XP_035350572.1| uncharacterized protein TRUGW13939_11573 [Talaromyces rugulosus]
 gb|QKX64399.1| hypothetical protein TRUGW13939_11573 [Talaromyces rugulosus]
 emb|CRG83261.1| Histone H2A [Talaromyces islandicus]
```

```
          Length=133

 Score = 171.4 bits (433),  Expect = 2E-52
 Identities = 113/119 (94%), Positives = 115/119 (97%), Gaps = 0/119 (0%)

Query  16   NAQSRSSKAGLAFPVGRVHRLLRKGNYAQRVGAGAPXXXXXXXXXXXXXXXXXXGNAARD  75
            N+QSRSSKAGLAFPVGRVHRLLRKGNYAQRVGAGAPVYLAAVLEYLAAEILELAGNAARD
Sbjct  15   NSQSRSSKAGLAFPVGRVHRLLRKGNYAQRVGAGAPVYLAAVLEYLAAEILELAGNAARD  74

Query  76   NKKTRIIPRHLQLAIRNDEELNKLLGHVTIAQGGVLPNIHQNLLPXXXXXXXXXSSMEL  134
            NKKTRIIPRHLQLAIRNDEELNKLLGHVTIAQGGVLPNIHQNLLPKKT K+GK  S EL
Sbjct  75   NKKTRIIPRHLQLAIRNDEELNKLLGHVTIAQGGVLPNIHQNLLPKKTPKSGKNPSQEL  133


>XP_006666427.1| histone H2A [Cordyceps militaris CM01]
 ref|XP_008603263.1| histone H2A-like protein [Beauveria bassiana ARSEF 2860]
 ref|XP_018708721.1| histone H2A [Cordyceps fumosorosea ARSEF 2679]
 gb|ATY64822.1| histone H2A [Cordyceps militaris]
 gb|KAF1737045.1| Histone H2A [Beauveria bassiana]
 gb|KGQ02647.1| Histone H2A [Beauveria bassiana D1-5]
 gb|OAA34581.1| Histone H2A [Beauveria brongniartii RCEF 3172]
 gb|OAA81899.1| histone H2A [Akanthomyces lecanii RCEF 1005]
 gb|TQV98326.1| histone H2A [Cordyceps javanica]
          Length=136

 Score = 171.4 bits (433),  Expect = 2E-52
 Identities = 105/105 (100%), Positives = 105/105 (100%), Gaps = 0/105 (0%)

Query  16   NAQSRSSKAGLAFPVGRVHRLLRKGNYAQRVGAGAPXXXXXXXXXXXXXXXXXXGNAARD  75
            NAQSRSSKAGLAFPVGRVHRLLRKGNYAQRVGAGAPVYLAAVLEYLAAEILELAGNAARD
Sbjct  16   NAQSRSSKAGLAFPVGRVHRLLRKGNYAQRVGAGAPVYLAAVLEYLAAEILELAGNAARD  75

Query  76   NKKTRIIPRHLQLAIRNDEELNKLLGHVTIAQGGVLPNIHQNLLP  120
            NKKTRIIPRHLQLAIRNDEELNKLLGHVTIAQGGVLPNIHQNLLP
```

```
Sbjct  76   NKKTRIIPRHLQLAIRNDEELNKLLGHVTIAQGGVLPNIHQNLLP  120


>XP_013321709.1| histone H2A [Exophiala xenobiotica]
 ref|XP_016231130.1| histone H2A [Exophiala spinifera]
 ref|XP_016263616.1| histone H2A [Exophiala oligosperma]
 gb|KIW10914.1| histone H2A [Exophiala spinifera]
 gb|KIW43400.1| histone H2A [Exophiala oligosperma]
 gb|KIW61125.1| histone H2A [Exophiala xenobiotica]
          Length=132

 Score = 171.4 bits (433),  Expect = 2E-52
 Identities = 105/105 (100%), Positives = 105/105 (100%), Gaps = 0/105 (0%)

Query  16   NAQSRSSKAGLAFPVGRVHRLLRKGNYAQRVGAGAPXXXXXXXXXXXXXXXXXXGNAARD  75
            NAQSRSSKAGLAFPVGRVHRLLRKGNYAQRVGAGAPVYLAAVLEYLAAEILELAGNAARD
Sbjct  15   NAQSRSSKAGLAFPVGRVHRLLRKGNYAQRVGAGAPVYLAAVLEYLAAEILELAGNAARD  74

Query  76   NKKTRIIPRHLQLAIRNDEELNKLLGHVTIAQGGVLPNIHQNLLP  120
            NKKTRIIPRHLQLAIRNDEELNKLLGHVTIAQGGVLPNIHQNLLP
Sbjct  75   NKKTRIIPRHLQLAIRNDEELNKLLGHVTIAQGGVLPNIHQNLLP  119


>XP_020122957.1| Histone H2A [Talaromyces atroroseus]
 gb|OKL62836.1| Histone H2A [Talaromyces atroroseus]
          Length=132

 Score = 171.4 bits (433),  Expect = 2E-52
 Identities = 105/105 (100%), Positives = 105/105 (100%), Gaps = 0/105 (0%)

Query  16   NAQSRSSKAGLAFPVGRVHRLLRKGNYAQRVGAGAPXXXXXXXXXXXXXXXXXXGNAARD  75
            NAQSRSSKAGLAFPVGRVHRLLRKGNYAQRVGAGAPVYLAAVLEYLAAEILELAGNAARD
Sbjct  15   NAQSRSSKAGLAFPVGRVHRLLRKGNYAQRVGAGAPVYLAAVLEYLAAEILELAGNAARD  74
```

```
Query  76   NKKTRIIPRHLQLAIRNDEELNKLLGHVTIAQGGVLPNIHQNLLP  120
            NKKTRIIPRHLQLAIRNDEELNKLLGHVTIAQGGVLPNIHQNLLP
Sbjct  75   NKKTRIIPRHLQLAIRNDEELNKLLGHVTIAQGGVLPNIHQNLLP  119


>XP_001929713.1| uncharacterized protein PODANS_5_5390 [Podospora anserina S mat+]
 sp|Q875B8.3| RecName: Full=Histone H2A [Podospora anserina]
 emb|VBB81311.1| Histone H2A [Podospora comata]
 emb|CAD60693.1| unnamed protein product [Podospora anserina]
 emb|CAP49213.1| unnamed protein product [Podospora anserina S mat+]
 emb|CDP29517.1| Histone H2A [Podospora anserina S mat+]
          Length=135

 Score = 171.4 bits (433),  Expect = 2E-52
 Identities = 105/105 (100%), Positives = 105/105 (100%), Gaps = 0/105 (0%)

Query  16   NAQSRSSKAGLAFPVGRVHRLLRKGNYAQRVGAGAPXXXXXXXXXXXXXXXXXXGNAARD  75
            NAQSRSSKAGLAFPVGRVHRLLRKGNYAQRVGAGAPVYLAAVLEYLAAEILELAGNAARD
Sbjct  16   NAQSRSSKAGLAFPVGRVHRLLRKGNYAQRVGAGAPVYLAAVLEYLAAEILELAGNAARD  75

Query  76   NKKTRIIPRHLQLAIRNDEELNKLLGHVTIAQGGVLPNIHQNLLP  120
            NKKTRIIPRHLQLAIRNDEELNKLLGHVTIAQGGVLPNIHQNLLP
Sbjct  76   NKKTRIIPRHLQLAIRNDEELNKLLGHVTIAQGGVLPNIHQNLLP  120


>XP_024707064.1| putative histone H2A [Aspergillus steynii IBT 23096]
 ref|XP_033424891.1| histone H2A [Aspergillus tanneri]
 gb|KAA8645530.1| histone H2A [Aspergillus tanneri]
 gb|PLB51762.1| putative histone H2A [Aspergillus steynii IBT 23096]
 gb|THC91432.1| hypothetical protein EYZ11_009099 [Aspergillus tanneri]
          Length=134

 Score = 171.4 bits (433),  Expect = 2E-52
 Identities = 105/105 (100%), Positives = 105/105 (100%), Gaps = 0/105 (0%)
```

```
Query  16   NAQSRSSKAGLAFPVGRVHRLLRKGNYAQRVGAGAPXXXXXXXXXXXXXXXXXXGNAARD  75
            NAQSRSSKAGLAFPVGRVHRLLRKGNYAQRVGAGAPVYLAAVLEYLAAEILELAGNAARD
Sbjct  15   NAQSRSSKAGLAFPVGRVHRLLRKGNYAQRVGAGAPVYLAAVLEYLAAEILELAGNAARD  74

Query  76   NKKTRIIPRHLQLAIRNDEELNKLLGHVTIAQGGVLPNIHQNLLP  120
            NKKTRIIPRHLQLAIRNDEELNKLLGHVTIAQGGVLPNIHQNLLP
Sbjct  75   NKKTRIIPRHLQLAIRNDEELNKLLGHVTIAQGGVLPNIHQNLLP  119


>XP_033436560.1| Histone H2A [Daldinia childiae]
 gb|KAF3061411.1| Histone H2A [Daldinia childiae]
 gb|OTB12596.1| hypothetical protein K445DRAFT_321043 [Daldinia sp. EC12]
          Length=136

 Score = 171.4 bits (433),  Expect = 2E-52
 Identities = 105/105 (100%), Positives = 105/105 (100%), Gaps = 0/105 (0%)

Query  16   NAQSRSSKAGLAFPVGRVHRLLRKGNYAQRVGAGAPXXXXXXXXXXXXXXXXXXGNAARD  75
            NAQSRSSKAGLAFPVGRVHRLLRKGNYAQRVGAGAPVYLAAVLEYLAAEILELAGNAARD
Sbjct  16   NAQSRSSKAGLAFPVGRVHRLLRKGNYAQRVGAGAPVYLAAVLEYLAAEILELAGNAARD  75

Query  76   NKKTRIIPRHLQLAIRNDEELNKLLGHVTIAQGGVLPNIHQNLLP  120
            NKKTRIIPRHLQLAIRNDEELNKLLGHVTIAQGGVLPNIHQNLLP
Sbjct  76   NKKTRIIPRHLQLAIRNDEELNKLLGHVTIAQGGVLPNIHQNLLP  120


>XP_020130993.1| histone h2a [Diplodia corticola]
 ref|XP_035363876.1| Histone H2A [Lasiodiplodia theobromae]
 gb|EKG10735.1| Histone H2A [Macrophomina phaseolina MS6]
 gb|KAF4305735.1| histone h2a [Botryosphaeria dothidea]
 gb|KKY14639.1| putative histone h2a [Diplodia seriata]
 gb|KAB2576277.1| Histone H2A [Lasiodiplodia theobromae]
 gb|KAF4535175.1| Histone H2A [Lasiodiplodia theobromae]
```

```
          Length=135

 Score = 171.4 bits (433),  Expect = 2E-52
 Identities = 105/105 (100%), Positives = 105/105 (100%), Gaps = 0/105 (0%)

Query  16   NAQSRSSKAGLAFPVGRVHRLLRKGNYAQRVGAGAPXXXXXXXXXXXXXXXXXXGNAARD  75
            NAQSRSSKAGLAFPVGRVHRLLRKGNYAQRVGAGAPVYLAAVLEYLAAEILELAGNAARD
Sbjct  16   NAQSRSSKAGLAFPVGRVHRLLRKGNYAQRVGAGAPVYLAAVLEYLAAEILELAGNAARD  75

Query  76   NKKTRIIPRHLQLAIRNDEELNKLLGHVTIAQGGVLPNIHQNLLP  120
            NKKTRIIPRHLQLAIRNDEELNKLLGHVTIAQGGVLPNIHQNLLP
Sbjct  76   NKKTRIIPRHLQLAIRNDEELNKLLGHVTIAQGGVLPNIHQNLLP  120
```
