# Supplementary material for: Effect of H2A.Z deletion is rescued by compensatory mutations in Fusarium graminearum
Source: PLoS Genet. 2020 Oct 22;16(10):e1009125. doi: 10.1371/journal.pgen.1009125 (PMC7608984; doi:10.1371/journal.pgen.1009125)
Supplement: S2 File — (PDF) [file pgen.1009125.s003.pdf]

```
BLASTP 2.10.1+
Reference: Stephen F. Altschul, Thomas L. Madden, Alejandro A. Sch&auml;ffer,
Jinghui Zhang, Zheng Zhang, Webb Miller, and David J. Lipman (1997), "Gapped
BLAST and PSI-BLAST: a new generation of protein database search programs",
Nucleic Acids Res. 25:3389-3402.

RID: HE2HSZZM01N


Database: refseq_protein
Query= FGRAMPH1_01T03973
Length=144

                                                                      Score     E
Sequences producing significant alignments:                          (Bits)  Value

ref|XP_009258393.1|  hypothetical protein FPSE_07000 [Fusarium...   248    2E-82
ref|XP_018249064.1|  histone H2A.Z [Fusarium oxysporum f. sp. ...   240    2E-79
ref|XP_003049781.1|  histone 2A variant [Fusarium vanettenii 7...   239    6E-79
ref|XP_024747021.1|  histone-fold-containing protein [Trichode...   238    2E-78
ref|XP_013949104.1|  hypothetical protein TRIATDRAFT_296920 [T...   236    1E-77
ref|XP_018177678.1|  histone H2A [Purpureocillium lilacinum] >...   235    4E-77
ref|XP_024759879.1|  hypothetical protein M441DRAFT_428737 [Tr...   234    9E-77
ref|XP_006691294.1|  hypothetical protein CTHT_0007640 [Chaeto...   233    3E-76
ref|XP_003657342.1|  H2A-like protein [Thermothielavioides ter...   232    3E-76
ref|XP_008595796.1|  Histone H2A [Beauveria bassiana ARSEF 286...   232    3E-76
ref|XP_003346683.1|  putative HTA1 protein [Sordaria macrospor...   232    5E-76
ref|XP_033442277.1|  Histone H2A.Z [Daldinia childiae] >gb|KAF...   232    5E-76
ref|XP_003666520.1|  histone H2A like protein [Thermothelomyce...   231    6E-76
ref|XP_022477125.1|  histone H2A.Z [Colletotrichum orchidophil...   231    6E-76
ref|XP_007830473.1|  Histone H2A.Z [Pestalotiopsis fici W106-1...   231    7E-76
ref|XP_008095165.1|  histone H2A.Z [Colletotrichum graminicola...   231    8E-76
ref|XP_009654826.1|  histone H2A.Z [Verticillium dahliae VdLs....   230    2E-75
ref|XP_024719387.1|  hypothetical protein M430DRAFT_28846 [Amo...   229    6E-75
```

```
ref|XP_018186095.1|  histone-fold-containing protein [Xylona h...   229    8E-75
ref|XP_008082453.1|  Histone-fold containing protein [Glarea l...   227    4E-74
ref|XP_031867203.1|  Histone H2A [Venustampulla echinocandica]...   227    5E-74
ref|XP_016646064.1|  hypothetical protein SAPIO_CDS1167 [Scedo...   227    5E-74
ref|XP_017994961.1|  Histone H2A.Z [Phialophora attinorum] >gb...   226    1E-73
ref|XP_030993423.1|  uncharacterized protein E0L32_007691 [Phi...   225    2E-73
ref|XP_001597856.1|  histone H2A [Sclerotinia sclerotiorum 198...   225    4E-73
ref|XP_018146336.1|  histone H2A [Pochonia chlamydosporia 170]...   225    4E-73
ref|XP_013283629.1|  histone H2A.Z [Fonsecaea pedrosoi CBS 271...   224    4E-73
ref|XP_018694917.1|  histone H2A.Z [Fonsecaea erecta] >gb|OAP6...   224    5E-73
ref|XP_007728785.1|  histone H2A.Z [Capronia epimyces CBS 606....   224    6E-73
ref|XP_016232314.1|  histone H2A.Z [Exophiala spinifera] >gb|K...   224    6E-73
ref|XP_018063296.1|  histone-fold-containing protein [Phialoce...   223    1E-72
ref|XP_028462876.1|  histone H2A.Z [Sodiomyces alkalinus F11] ...   223    2E-72
ref|XP_024733061.1|  histone-fold-containing protein, partial ...   222    2E-72
ref|XP_016224592.1|  histone H2A.Z [Exophiala mesophila] >gb|K...   223    2E-72
ref|XP_007293349.1|  histone H2A [Marssonina brunnea f. sp. 'm...   222    3E-72
ref|XP_013266185.1|  histone H2A.Z [Exophiala aquamarina CBS 1...   222    3E-72
ref|XP_031001079.1|  Histone H2A.Z [Lachnellula hyalina] >gb|T...   221    9E-72
ref|XP_020061199.1|  uncharacterized protein ASPACDRAFT_49223 ...   220    2E-71
ref|XP_002151102.1|  histone H2A [Talaromyces marneffei ATCC 1...   220    3E-71
ref|XP_002341942.1|  histone H2A [Talaromyces stipitatus ATCC ...   219    4E-71
ref|XP_020120058.1|  Histone H2A.Z [Talaromyces atroroseus] >g...   219    6E-71
ref|XP_024685068.1|  histone-fold-containing protein [Aspergil...   219    6E-71
ref|XP_001266338.1|  histone H2A [Aspergillus fischeri NRRL 18...   219    6E-71
ref|XP_035341716.1|  uncharacterized protein TRUGW13939_02632 ...   219    6E-71
ref|XP_022578503.1|  hypothetical protein ASPZODRAFT_153973 [P...   219    7E-71
ref|XP_001820387.1|  unnamed protein product [Aspergillus oryz...   218    9E-71
ref|XP_016760042.1|  histone H2A [Sphaerulina musiva SO2202] >...   218    1E-70
ref|XP_002544485.1|  histone H2A variant [Uncinocarpus reesii ...   218    1E-70
ref|XP_013326979.1|  Histone H2A [Rasamsonia emersonii CBS 393...   218    1E-70
ref|XP_028481541.1|  histone-fold-containing protein [Byssochl...   218    1E-70

ALIGNMENTS
```

```
>XP_009258393.1| hypothetical protein FPSE_07000 [Fusarium pseudograminearum CS3096]
 ref|XP_011317451.1| histone H2A.Z [Fusarium graminearum PH-1]
 ref|XP_025589171.1| uncharacterized protein FVRRES_01963 [Fusarium venenatum]
 ref|XP_031017970.1| uncharacterized protein FIESC28_03855 [Fusarium coffeatum]
 sp|Q4IMD1.1| RecName: Full=Histone H2A.Z [Fusarium graminearum PH-1]
 gb|EYB22689.1| hypothetical protein FG05_01627 [Fusarium graminearum]
 gb|KAF0645097.1| hypothetical protein FPSE5266_07000 [Fusarium pseudograminearum]
 gb|KAF5238922.1| hypothetical protein FAUST_5258 [Fusarium austroamericanum]
 gb|KPA45311.1| histone [Fusarium langsethiae]
 gb|OBS27731.1| hypothetical protein FPOA_01673 [Fusarium poae]
 gb|PTD11388.1| Histone H2A.Z [Fusarium culmorum]
 gb|RFN49439.1| histone h2a.z [Fusarium fasciculatum]
 gb|RGP68747.1| histone [Fusarium longipes]
 gb|RGP75417.1| histone [Fusarium sporotrichioides]
          Length=144

 Score = 248.1 bits (632),  Expect = 2E-82
 Identities = 123/123 (100%), Positives = 123/123 (100%), Gaps = 0/123 (0%)

Query  22   EGANKKQQSHSARAGLQFPCGRVKRFLKQNTQQKMRVGAKAAVYVTAVLEYLTAEVLELA  81
            EGANKKQQSHSARAGLQFPCGRVKRFLKQNTQQKMRVGAKAAVYVTAVLEYLTAEVLELA
Sbjct  22   EGANKKQQSHSARAGLQFPCGRVKRFLKQNTQQKMRVGAKAAVYVTAVLEYLTAEVLELA  81

Query  82   GNAAKDLKVKRITPRHLQLAIRGDEELDTLIRATIAYGGVLPHINRALLLKVEQKKKAKA  141
            GNAAKDLKVKRITPRHLQLAIRGDEELDTLIRATIAYGGVLPHINRALLLKVEQKKKAKA
Sbjct  82   GNAAKDLKVKRITPRHLQLAIRGDEELDTLIRATIAYGGVLPHINRALLLKVEQKKKAKA  141

Query  142  LEG  144
            LEG
Sbjct  142  LEG  144


>XP_018249064.1| histone H2A.Z [Fusarium oxysporum f. sp. lycopersici 4287]
 ref|XP_018756722.1| histone H2A.Z [Fusarium verticillioides 7600]
```

```
ref|XP_023423754.1| probable histone H2A F/Z family member HTZ1 [Fusarium fujikuroi IMI 58289]
ref|XP_031029425.1| histone H2A.Z [Fusarium oxysporum NRRL 32931]
ref|XP_031071918.1| histone H2A.Z [Fusarium odoratissimum NRRL 54006]
ref|XP_031076197.1| probable histone H2A F/Z family member HTZ1 [Fusarium proliferatum ET1]
gb|EGU74041.1| hypothetical protein FOXB_15431 [Fusarium oxysporum Fo5176]
gb|EMT64689.1| Histone H2A.Z [Fusarium odoratissimum]
gb|ENH72633.1| Histone H2A.Z [Fusarium oxysporum f. sp. cubense race 1]
gb|EWZ49393.1| histone H2A.Z [Fusarium oxysporum Fo47]
gb|EWZ89156.1| histone H2A.Z [Fusarium oxysporum f. sp. lycopersici MN25]
gb|EXA54483.1| histone H2A.Z [Fusarium oxysporum f. sp. pisi HDV247]
gb|EXK46634.1| histone H2A.Z [Fusarium oxysporum f. sp. melonis 26406]
gb|EXK93559.1| histone H2A.Z [Fusarium oxysporum f. sp. raphani 54005]
gb|EXL46207.1| histone H2A.Z [Fusarium oxysporum f. sp. radicis-lycopersici 26381]
gb|EXL80982.1| histone H2A.Z [Fusarium oxysporum f. sp. conglutinans race 2 54008]
gb|EXM29021.1| histone H2A.Z [Fusarium oxysporum f. sp. vasinfectum 25433]
gb|KAF4344871.1| histone H2A [Fusarium beomiforme]
gb|KAF4431838.1| histone H2A.Z [Fusarium austroafricanum]
gb|KAF4432724.1| Histone H2A [Fusarium acutatum]
gb|KAF4498485.1| Histone H2A [Fusarium agapanthi]
gb|KAF5252432.1| hypothetical protein FANTH_2568 [Fusarium anthophilum]
gb|KAF5259872.1| hypothetical protein FOXYS1_9494 [Fusarium oxysporum]
gb|KLO86595.1| putative histone H2A F/Z family member HTZ1 [Fusarium fujikuroi]
gb|PNP77788.1| hypothetical protein FNYG_08869 [Fusarium nygamai]
gb|RBA11284.1| histone H2A.Z [Fusarium proliferatum]
gb|RBQ99160.1| hypothetical protein FVER53263_09724 [Fusarium verticillioides]
gb|RKK29374.1| hypothetical protein BFJ65_g1300 [Fusarium oxysporum f. sp. cepae]
gb|RYC91200.1| hypothetical protein BFJ63_vAg6050 [Fusarium oxysporum f. sp. narcissi]
gb|TVY70680.1| Histone H2A.Z [Fusarium oxysporum f. sp. cubense]
emb|CVL02796.1| probable histone H2A F/Z family member HTZ1 [Fusarium mangiferae]
         Length=143

Score = 240.3 bits (612),  Expect = 2E-79
Identities = 119/121 (98%), Positives = 119/121 (98%), Gaps = 0/121 (0%)
```

```
Query  24   ANKKQQSHSARAGLQFPCGRVKRFLKQNTQQKMRVGAKAAVYVTAVLEYLTAEVLELAGN  83
              KKQQSHSARAGLQFPCGRVKRFLKQNTQQKMRVGAKAAVYVTAVLEYLTAEVLELAGN
Sbjct  23   GTKKQQSHSARAGLQFPCGRVKRFLKQNTQQKMRVGAKAAVYVTAVLEYLTAEVLELAGN  82

Query  84   AAKDLKVKRITPRHLQLAIRGDEELDTLIRATIAYGGVLPHINRALLLKVEQKKKAKALE  143
            AAKDLKVKRITPRHLQLAIRGDEELDTLIRATIAYGGVLPHINRALLLKVEQKKKAKALE
Sbjct  83   AAKDLKVKRITPRHLQLAIRGDEELDTLIRATIAYGGVLPHINRALLLKVEQKKKAKALE  142

Query  144  G  144
            G
Sbjct  143  G  143


>XP_003049781.1| histone 2A variant [Fusarium vanettenii 77-13-4]
 gb|RMJ02355.1| Histone H2A.Z [Fusarium kuroshium]
 gb|RSL51092.1| Histone H2A.Z [Fusarium floridanum]
 gb|RSL64309.1| Histone H2A.Z [Fusarium sp. AF-8]
 gb|RSM03539.1| Histone H2A.Z [Fusarium sp. AF-4]
 gb|RSM16763.1| Histone H2A.Z [Fusarium ambrosium]
 gb|RTE69270.1| Histone H2A.Z [Fusarium euwallaceae]
          Length=143

 Score = 239.2 bits (609),  Expect = 6E-79
 Identities = 118/121 (97%), Positives = 119/121 (98%), Gaps = 0/121 (0%)

Query  24   ANKKQQSHSARAGLQFPCGRVKRFLKQNTQQKMRVGAKAAVYVTAVLEYLTAEVLELAGN  83
             +KKQQSHSARAGLQFPCGRVKRFLKQNTQ KMRVGAKAAVYVTAVLEYLTAEVLELAGN
Sbjct  23   GSKKQQSHSARAGLQFPCGRVKRFLKQNTQNKMRVGAKAAVYVTAVLEYLTAEVLELAGN  82

Query  84   AAKDLKVKRITPRHLQLAIRGDEELDTLIRATIAYGGVLPHINRALLLKVEQKKKAKALE  143
            AAKDLKVKRITPRHLQLAIRGDEELDTLIRATIAYGGVLPHINRALLLKVEQKKKAKALE
Sbjct  83   AAKDLKVKRITPRHLQLAIRGDEELDTLIRATIAYGGVLPHINRALLLKVEQKKKAKALE  142

Query  144  G  144
```

```
            G
Sbjct  143  G  143


>XP_024747021.1| histone-fold-containing protein [Trichoderma citrinoviride]
 gb|PTB63701.1| histone-fold-containing protein [Trichoderma citrinoviride]
 gb|PTB72082.1| histone-fold-containing protein [Trichoderma longibrachiatum ATCC 18648]
          Length=142

 Score = 237.7 bits (605),  Expect = 2E-78
 Identities = 117/120 (97%), Positives = 118/120 (98%), Gaps = 0/120 (0%)

Query  24   ANKKQQSHSARAGLQFPCGRVKRFLKQNTQQKMRVGAKAAVYVTAVLEYLTAEVLELAGN  83
             NKKQQSHSARAGLQFPCGRVKRFLKQNTQ KMRVGAKAAVYVTAVLEYLTAEVLELAGN
Sbjct  22   GNKKQQSHSARAGLQFPCGRVKRFLKQNTQNKMRVGAKAAVYVTAVLEYLTAEVLELAGN  81

Query  84   AAKDLKVKRITPRHLQLAIRGDEELDTLIRATIAYGGVLPHINRALLLKVEQKKKAKALE  143
            AAKDLKVKRITPRHLQLAIRGDEELDTLIRATIA+GGVLPHINRALLLKVEQKKKAKALE
Sbjct  82   AAKDLKVKRITPRHLQLAIRGDEELDTLIRATIAFGGVLPHINRALLLKVEQKKKAKALE  141


>XP_013949104.1| hypothetical protein TRIATDRAFT_296920 [Trichoderma atroviride IMI 206040]
 ref|XP_013950361.1| hypothetical protein TRIVIDRAFT_39222 [Trichoderma virens Gv29-8]
 gb|EHK16165.1| hypothetical protein TRIVIDRAFT_39222 [Trichoderma virens Gv29-8]
 gb|EHK50949.1| hypothetical protein TRIATDRAFT_296920 [Trichoderma atroviride IMI 206040]
          Length=142

 Score = 236.1 bits (601),  Expect = 1E-77
 Identities = 119/122 (97%), Positives = 120/122 (98%), Gaps = 1/122 (1%)

Query  22   EGANKKQQSHSARAGLQFPCGRVKRFLKQNTQQKMRVGAKAAVYVTAVLEYLTAEVLELA  81
            EGA KKQQSHSARAGLQFPCGRVKRFLKQNTQ KMRVGAKAAVYVTAVLEYLTAEVLELA
Sbjct  21   EGA-KKQQSHSARAGLQFPCGRVKRFLKQNTQNKMRVGAKAAVYVTAVLEYLTAEVLELA  79
```

```
Query  82   GNAAKDLKVKRITPRHLQLAIRGDEELDTLIRATIAYGGVLPHINRALLLKVEQKKKAKA  141
            GNAAKDLKVKRITPRHLQLAIRGDEELDTLIRATIA+GGVLPHINRALLLKVEQKKKAKA
Sbjct  80   GNAAKDLKVKRITPRHLQLAIRGDEELDTLIRATIAFGGVLPHINRALLLKVEQKKKAKA  139

Query  142  LE  143
            LE
Sbjct  140  LE  141


>XP_018177678.1| histone H2A [Purpureocillium lilacinum]
 gb|OAQ79641.1| histone H2A [Purpureocillium lilacinum]
 gb|OAQ88959.1| histone H2A [Purpureocillium lilacinum]
          Length=142

 Score = 234.6 bits (597),  Expect = 4E-77
 Identities = 116/120 (96%), Positives = 118/120 (98%), Gaps = 0/120 (0%)

Query  24   ANKKQQSHSARAGLQFPCGRVKRFLKQNTQQKMRVGAKAAVYVTAVLEYLTAEVLELAGN  83
             +KKQQSHSARAGLQFPCGRVKRFLKQNTQ KMRVGAKAAVYVTAVLEYLTAEVLELAGN
Sbjct  22   GHKKQQSHSARAGLQFPCGRVKRFLKQNTQNKMRVGAKAAVYVTAVLEYLTAEVLELAGN  81

Query  84   AAKDLKVKRITPRHLQLAIRGDEELDTLIRATIAYGGVLPHINRALLLKVEQKKKAKALE  143
            AAKDLKVKRITPRHLQLAIRGDEELDTLIRATIA+GGVLPHINRALLLKVEQKKKAKALE
Sbjct  82   AAKDLKVKRITPRHLQLAIRGDEELDTLIRATIAFGGVLPHINRALLLKVEQKKKAKALE  141


>XP_024759879.1| hypothetical protein M441DRAFT_428737 [Trichoderma asperellum CBS 433.97]
 gb|PTB40202.1| hypothetical protein M441DRAFT_428737 [Trichoderma asperellum CBS 433.97]
          Length=142

 Score = 233.8 bits (595),  Expect = 9E-77
 Identities = 118/122 (96%), Positives = 119/122 (98%), Gaps = 1/122 (1%)

Query  22   EGANKKQQSHSARAGLQFPCGRVKRFLKQNTQQKMRVGAKAAVYVTAVLEYLTAEVLELA  81
```

```
             EGA KKQQSHSARAGLQFPCGRVKRFLKQ TQ KMRVGAKAAVYVTAVLEYLTAEVLELA
Sbjct  21   EGA-KKQQSHSARAGLQFPCGRVKRFLKQKTQSKMRVGAKAAVYVTAVLEYLTAEVLELA  79

Query  82   GNAAKDLKVKRITPRHLQLAIRGDEELDTLIRATIAYGGVLPHINRALLLKVEQKKKAKA  141
            GNAAKDLKVKRITPRHLQLAIRGDEELDTLIRATIA+GGVLPHINRALLLKVEQKKKAKA
Sbjct  80   GNAAKDLKVKRITPRHLQLAIRGDEELDTLIRATIAFGGVLPHINRALLLKVEQKKKAKA  139

Query  142  LE  143
            LE
Sbjct  140  LE  141


>XP_006691294.1| hypothetical protein CTHT_0007640 [Chaetomium thermophilum var. thermophilum
DSM 1495]
 gb|EGS24052.1| hypothetical protein CTHT_0007640 [Chaetomium thermophilum var. thermophilum
DSM 1495]
          Length=143

 Score = 232.6 bits (592),  Expect = 3E-76
 Identities = 115/118 (97%), Positives = 116/118 (98%), Gaps = 0/118 (0%)

Query  26   KKQQSHSARAGLQFPCGRVKRFLKQNTQQKMRVGAKAAVYVTAVLEYLTAEVLELAGNAA  85
            KKQQSHSARAGLQFPCGRVKRFLKQNTQ KMRVGAKAAVYVTAVLEYLTAEVLELAGNAA
Sbjct  25   KKQQSHSARAGLQFPCGRVKRFLKQNTQNKMRVGAKAAVYVTAVLEYLTAEVLELAGNAA  84

Query  86   KDLKVKRITPRHLQLAIRGDEELDTLIRATIAYGGVLPHINRALLLKVEQKKKAKALE  143
            KDLKVKRITPRHLQLAIRGDEELDTLIRATIA+GGVLPHINRALLLKVEQKKKAKA E
Sbjct  85   KDLKVKRITPRHLQLAIRGDEELDTLIRATIAFGGVLPHINRALLLKVEQKKKAKAAE  142


>XP_003657342.1| H2A-like protein [Thermothielavioides terrestris NRRL 8126]
 gb|AEO71006.1| H2A-like protein [Thermothielavioides terrestris NRRL 8126]
 emb|SPQ20668.1| b1ca90ce-d1d8-43b4-b627-f7dd4f806fa3 [Thermothielavioides terrestris]
          Length=142
```

```
 Score = 232.3 bits (591),  Expect = 3E-76
 Identities = 115/118 (97%), Positives = 116/118 (98%), Gaps = 0/118 (0%)

Query  26   KKQQSHSARAGLQFPCGRVKRFLKQNTQQKMRVGAKAAVYVTAVLEYLTAEVLELAGNAA  85
            KKQQSHSARAGLQFPCGRVKRFLKQNTQ KMRVGAKAAVYVTAVLEYLTAEVLELAGNAA
Sbjct  24   KKQQSHSARAGLQFPCGRVKRFLKQNTQNKMRVGAKAAVYVTAVLEYLTAEVLELAGNAA  83

Query  86   KDLKVKRITPRHLQLAIRGDEELDTLIRATIAYGGVLPHINRALLLKVEQKKKAKALE  143
            KDLKVKRITPRHLQLAIRGDEELDTLIRATIA+GGVLPHINRALLLKVEQKKKAKA E
Sbjct  84   KDLKVKRITPRHLQLAIRGDEELDTLIRATIAFGGVLPHINRALLLKVEQKKKAKAAE  141


>XP_008595796.1| Histone H2A [Beauveria bassiana ARSEF 2860]
 gb|KAF1735357.1| Histone H2A.Z [Beauveria bassiana]
 gb|KGQ10102.1| Histone H2A.Z [Beauveria bassiana D1-5]
 gb|EJP68475.1| Histone H2A [Beauveria bassiana ARSEF 2860]
 gb|PMB68561.1| Histone H2A.Z [Beauveria bassiana]
 gb|PQK15066.1| hypothetical protein BB8028_0005g05820 [Beauveria bassiana]
          Length=143

 Score = 232.3 bits (591),  Expect = 3E-76
 Identities = 115/118 (97%), Positives = 115/118 (97%), Gaps = 0/118 (0%)

Query  26   KKQQSHSARAGLQFPCGRVKRFLKQNTQQKMRVGAKAAVYVTAVLEYLTAEVLELAGNAA  85
            KKQQSHSARAGLQFPCGRVKRFLKQNTQ KMRVGAKAAVYVTAVLEYLTAEVLELAGNAA
Sbjct  25   KKQQSHSARAGLQFPCGRVKRFLKQNTQSKMRVGAKAAVYVTAVLEYLTAEVLELAGNAA  84

Query  86   KDLKVKRITPRHLQLAIRGDEELDTLIRATIAYGGVLPHINRALLLKVEQKKKAKALE  143
            KDLKVKRITPRHLQLAIRGDEELDTLIRATIAYGGVLPHINRALLLKVEQKKK K LE
Sbjct  85   KDLKVKRITPRHLQLAIRGDEELDTLIRATIAYGGVLPHINRALLLKVEQKKKNKTLE  142


>XP_003346683.1| putative HTA1 protein [Sordaria macrospora k-hell]
```

```
 ref|XP_009852423.1| hypothetical protein NEUTE1DRAFT_84413 [Neurospora tetrasperma FGSC 2508]
 ref|XP_011393560.1| histone H2A.Z, variant 2 [Neurospora crassa OR74A]
 ref|XP_011393561.1| histone H2A.Z [Neurospora crassa OR74A]
 ref|XP_011393562.1| histone H2A.Z, variant 1 [Neurospora crassa OR74A]
 sp|Q873G4.1| RecName: Full=Histone H2A.Z [Neurospora crassa OR74A]
 gb|EGZ70243.1| histone-fold-containing protein [Neurospora tetrasperma FGSC 2509]
 gb|KAA8628690.1| hypothetical protein SMACR_04116 [Sordaria macrospora]
 gb|KHE80044.1| hypothetical protein GE21DRAFT_2945 [Neurospora crassa]
 gb|EGO56866.1| hypothetical protein NEUTE1DRAFT_84413 [Neurospora tetrasperma FGSC 2508]
 gb|ESA43531.1| histone H2A.Z [Neurospora crassa OR74A]
          Length=143

 Score = 231.9 bits (590),  Expect = 5E-76
 Identities = 115/118 (97%), Positives = 116/118 (98%), Gaps = 0/118 (0%)

Query  26   KKQQSHSARAGLQFPCGRVKRFLKQNTQQKMRVGAKAAVYVTAVLEYLTAEVLELAGNAA  85
            KKQQSHSARAGLQFPCGRVKRFLKQNTQ KMRVGAKAAVYVTAVLEYLTAEVLELAGNAA
Sbjct  25   KKQQSHSARAGLQFPCGRVKRFLKQNTQNKMRVGAKAAVYVTAVLEYLTAEVLELAGNAA  84

Query  86   KDLKVKRITPRHLQLAIRGDEELDTLIRATIAYGGVLPHINRALLLKVEQKKKAKALE  143
            KDLKVKRITPRHLQLAIRGDEELDTLIRATIA+GGVLPHINRALLLKVEQKKKAKA E
Sbjct  85   KDLKVKRITPRHLQLAIRGDEELDTLIRATIAFGGVLPHINRALLLKVEQKKKAKAQE  142


>XP_033442277.1| Histone H2A.Z [Daldinia childiae]
 gb|KAF3071240.1| Histone H2A.Z [Daldinia childiae]
          Length=143

 Score = 231.9 bits (590),  Expect = 5E-76
 Identities = 115/121 (95%), Positives = 117/121 (97%), Gaps = 0/121 (0%)

Query  24   ANKKQQSHSARAGLQFPCGRVKRFLKQNTQQKMRVGAKAAVYVTAVLEYLTAEVLELAGN  83
            A KKQQSHSARAGLQFPCGRVKRFLKQNTQ KMRVGAKAAVYVTAVLEYLTAEVLELAGN
Sbjct  23   APKKQQSHSARAGLQFPCGRVKRFLKQNTQNKMRVGAKAAVYVTAVLEYLTAEVLELAGN  82
```

```
Query  84   AAKDLKVKRITPRHLQLAIRGDEELDTLIRATIAYGGVLPHINRALLLKVEQKKKAKALE  143
            AAKDLKVKRITPRHLQLAIRGDEELDTLIRATIA+GGVLPHINRALLLKVEQKKK KA+E
Sbjct  83   AAKDLKVKRITPRHLQLAIRGDEELDTLIRATIAFGGVLPHINRALLLKVEQKKKNKAIE  142

Query  144  G  144

Sbjct  143  A  143


>XP_003666520.1| histone H2A like protein [Thermothelomyces thermophilus ATCC 42464]
 gb|AEO61275.1| histone H2A like protein [Thermothelomyces thermophilus ATCC 42464]
          Length=143

 Score = 231.5 bits (589),  Expect = 6E-76
 Identities = 115/118 (97%), Positives = 116/118 (98%), Gaps = 0/118 (0%)

Query  26   KKQQSHSARAGLQFPCGRVKRFLKQNTQQKMRVGAKAAVYVTAVLEYLTAEVLELAGNAA  85
            KKQQSHSARAGLQFPCGRVKRFLKQNTQ KMRVGAKAAVYVTAVLEYLTAEVLELAGNAA
Sbjct  25   KKQQSHSARAGLQFPCGRVKRFLKQNTQNKMRVGAKAAVYVTAVLEYLTAEVLELAGNAA  84

Query  86   KDLKVKRITPRHLQLAIRGDEELDTLIRATIAYGGVLPHINRALLLKVEQKKKAKALE  143
            KDLKVKRITPRHLQLAIRGDEELDTLIRATIA+GGVLPHINRALLLKVEQKKKAKA E
Sbjct  85   KDLKVKRITPRHLQLAIRGDEELDTLIRATIAFGGVLPHINRALLLKVEQKKKAKAPE  142


>XP_022477125.1| histone H2A.Z [Colletotrichum orchidophilum]
 ref|XP_035329009.1| histone H2A.Z [Colletotrichum scovillei]
 gb|EXF80862.1| histone H2A.Z [Colletotrichum fioriniae PJ7]
 gb|KXH41191.1| histone H2A.Z [Colletotrichum simmondsii]
 gb|KXH65205.1| histone H2A.Z [Colletotrichum nymphaeae SA-01]
 gb|KXH67986.1| histone H2A.Z [Colletotrichum salicis]
 gb|KZL76886.1| histone H2A [Colletotrichum tofieldiae]
 gb|OHX00308.1| histone [Colletotrichum incanum]
```

```
         Length=141

 Score = 231.5 bits (589),  Expect = 6E-76
 Identities = 115/118 (97%), Positives = 116/118 (98%), Gaps = 0/118 (0%)

Query  26   KKQQSHSARAGLQFPCGRVKRFLKQNTQQKMRVGAKAAVYVTAVLEYLTAEVLELAGNAA  85
            KKQQSHSARAGLQFPCGRVKRFLK NTQ KMRVGAKAAVYVTAVLEYLTAEVLELAGNAA
Sbjct  23   KKQQSHSARAGLQFPCGRVKRFLKANTQNKMRVGAKAAVYVTAVLEYLTAEVLELAGNAA  82

Query  86   KDLKVKRITPRHLQLAIRGDEELDTLIRATIAYGGVLPHINRALLLKVEQKKKAKALE  143
            KDLKVKRITPRHLQLAIRGDEELDTLIRATIA+GGVLPHINRALLLKVEQKKKAKALE
Sbjct  83   KDLKVKRITPRHLQLAIRGDEELDTLIRATIAFGGVLPHINRALLLKVEQKKKAKALE  140


>XP_007830473.1| Histone H2A.Z [Pestalotiopsis fici W106-1]
 gb|ETS85676.1| Histone H2A.Z [Pestalotiopsis fici W106-1]
 gb|KAF2998782.1| histone H2A.Z [Neopestalotiopsis sp. 37M]
         Length=143

 Score = 231.5 bits (589),  Expect = 7E-76
 Identities = 114/118 (96%), Positives = 116/118 (98%), Gaps = 0/118 (0%)

Query  26   KKQQSHSARAGLQFPCGRVKRFLKQNTQQKMRVGAKAAVYVTAVLEYLTAEVLELAGNAA  85
            KKQQSHSARAGLQFPCGRVKRFLKQNTQ KMRVGAKAAVYVTAVLEYLTAEVLELAGNAA
Sbjct  25   KKQQSHSARAGLQFPCGRVKRFLKQNTQNKMRVGAKAAVYVTAVLEYLTAEVLELAGNAA  84

Query  86   KDLKVKRITPRHLQLAIRGDEELDTLIRATIAYGGVLPHINRALLLKVEQKKKAKALE  143
            KDLKVKRITPRHLQLAIRGDEELDTLIRATIA+GGVLPHINRALLLKVEQKKK KA+E
Sbjct  85   KDLKVKRITPRHLQLAIRGDEELDTLIRATIAFGGVLPHINRALLLKVEQKKKNKAIE  142


>XP_008095165.1| histone H2A.Z [Colletotrichum graminicola M1.001]
 gb|EFQ31145.1| histone H2A.Z [Colletotrichum graminicola M1.001]
         Length=141
```

```
 Score = 231.1 bits (588),  Expect = 8E-76
 Identities = 115/118 (97%), Positives = 116/118 (98%), Gaps = 0/118 (0%)

Query  26   KKQQSHSARAGLQFPCGRVKRFLKQNTQQKMRVGAKAAVYVTAVLEYLTAEVLELAGNAA  85
            KKQQSHSARAGLQFPCGRVKRFLK NTQ KMRVGAKAAVYVTAVLEYLTAEVLELAGNAA
Sbjct  23   KKQQSHSARAGLQFPCGRVKRFLKANTQNKMRVGAKAAVYVTAVLEYLTAEVLELAGNAA  82

Query  86   KDLKVKRITPRHLQLAIRGDEELDTLIRATIAYGGVLPHINRALLLKVEQKKKAKALE  143
            KDLKVKRITPRHLQLAIRGDEELDTLIRATIA+GGVLPHINRALLLKVEQKKKAKALE
Sbjct  83   KDLKVKRITPRHLQLAIRGDEELDTLIRATIAFGGVLPHINRALLLKVEQKKKAKALE  140


>XP_009654826.1| histone H2A.Z [Verticillium dahliae VdLs.17]
 ref|XP_028497421.1| histone H2A.Z [Verticillium nonalfalfae]
 gb|KAF3349496.1| GTP-binding protein 1 [Verticillium dahliae VDG2]
 gb|KAF3358626.1| hypothetical protein VdG1_00186 [Verticillium dahliae VDG1]
 gb|PNH32794.1| hypothetical protein BJF96_g4001 [Verticillium dahliae]
 gb|EGY16462.1| histone H2A.Z [Verticillium dahliae VdLs.17]
 gb|PNH38822.1| hypothetical protein VD0004_g8037 [Verticillium dahliae]
          Length=142

 Score = 230.3 bits (586),  Expect = 2E-75
 Identities = 114/122 (93%), Positives = 116/122 (95%), Gaps = 0/122 (0%)

Query  22   EGANKKQQSHSARAGLQFPCGRVKRFLKQNTQQKMRVGAKAAVYVTAVLEYLTAEVLELA  81
            +G  KKQQSHSARAGLQFPCGRVKRFLK NTQ KMRVGAKAAVY TAVLEYLTAEVLELA
Sbjct  20   DGGPKKQQSHSARAGLQFPCGRVKRFLKSNTQNKMRVGAKAAVYTTAVLEYLTAEVLELA  79

Query  82   GNAAKDLKVKRITPRHLQLAIRGDEELDTLIRATIAYGGVLPHINRALLLKVEQKKKAKA  141
            GNAAKDLKVKRITPRHLQLAIRGDEELDTLIRATIA+GGVLPHINRALLLKVEQKKKAK
Sbjct  80   GNAAKDLKVKRITPRHLQLAIRGDEELDTLIRATIAFGGVLPHINRALLLKVEQKKKAKQ  139

Query  142  LE  143
```

```
            LE
Sbjct  140  LE  141


>XP_024719387.1| hypothetical protein M430DRAFT_28846 [Amorphotheca resinae ATCC 22711]
 gb|PSS14788.1| hypothetical protein M430DRAFT_28846 [Amorphotheca resinae ATCC 22711]
          Length=142

 Score = 229.2 bits (583),  Expect = 6E-75
 Identities = 112/119 (94%), Positives = 117/119 (98%), Gaps = 0/119 (0%)

Query  25   NKKQQSHSARAGLQFPCGRVKRFLKQNTQQKMRVGAKAAVYVTAVLEYLTAEVLELAGNA  84
            +KKQQSHS++AGLQFPCGRVKRFLK NTQ KMRVGAKAAVYVTAVLEYLTAEVLELAGNA
Sbjct  23   SKKQQSHSSKAGLQFPCGRVKRFLKNNTQNKMRVGAKAAVYVTAVLEYLTAEVLELAGNA  82

Query  85   AKDLKVKRITPRHLQLAIRGDEELDTLIRATIAYGGVLPHINRALLLKVEQKKKAKALE  143
            AKDLKVKRITPRHLQLAIRGDEELDTLIRATIA+GGVLPHINRALLLKVEQKKKAKA+E
Sbjct  83   AKDLKVKRITPRHLQLAIRGDEELDTLIRATIAFGGVLPHINRALLLKVEQKKKAKAIE  141


>XP_018186095.1| histone-fold-containing protein [Xylona heveae TC161]
 gb|KZF20540.1| histone-fold-containing protein [Xylona heveae TC161]
          Length=139

 Score = 228.8 bits (582),  Expect = 8E-75
 Identities = 112/122 (91%), Positives = 117/122 (96%), Gaps = 0/122 (0%)

Query  22   EGANKKQQSHSARAGLQFPCGRVKRFLKQNTQQKMRVGAKAAVYVTAVLEYLTAEVLELA  81
            E A K Q+SHSA+AGLQFPCGRVKRFLK NTQ KMRVGAKAAVYVTAVLEYLTAEVLELA
Sbjct  17   EAAGKSQKSHSAKAGLQFPCGRVKRFLKNNTQNKMRVGAKAAVYVTAVLEYLTAEVLELA  76

Query  82   GNAAKDLKVKRITPRHLQLAIRGDEELDTLIRATIAYGGVLPHINRALLLKVEQKKKAKA  141
            GNAAKDLKVKRITPRHLQLAIRGDEELDTLIRATIA+GGVLPHINRALLLKVEQKKK+KA
Sbjct  77   GNAAKDLKVKRITPRHLQLAIRGDEELDTLIRATIAFGGVLPHINRALLLKVEQKKKSKA  136
```

```
Query  142  LE  143
            +E
Sbjct  137  IE  138


>XP_008082453.1| Histone-fold containing protein [Glarea lozoyensis ATCC 20868]
 gb|EPE31042.1| Histone-fold containing protein [Glarea lozoyensis ATCC 20868]
          Length=142

 Score = 226.9 bits (577),  Expect = 4E-74
 Identities = 111/119 (93%), Positives = 116/119 (97%), Gaps = 0/119 (0%)

Query  25   NKKQQSHSARAGLQFPCGRVKRFLKQNTQQKMRVGAKAAVYVTAVLEYLTAEVLELAGNA  84
            +KKQQSHS++AGLQFPCGRVKRFLK NTQ KMRVGAKAAVYVTAVLEYLTAEVLELAGNA
Sbjct  23   SKKQQSHSSKAGLQFPCGRVKRFLKNNTQNKMRVGAKAAVYVTAVLEYLTAEVLELAGNA  82

Query  85   AKDLKVKRITPRHLQLAIRGDEELDTLIRATIAYGGVLPHINRALLLKVEQKKKAKALE  143
            AKDLKVKRITPRHLQLAIRGDEELDTLIRATIA+GGVLPHINRALLLKVEQKKK KA+E
Sbjct  83   AKDLKVKRITPRHLQLAIRGDEELDTLIRATIAFGGVLPHINRALLLKVEQKKKNKAIE  141


>XP_031867203.1| Histone H2A [Venustampulla echinocandica]
 gb|RDL33921.1| Histone H2A [Venustampulla echinocandica]
          Length=142

 Score = 226.9 bits (577),  Expect = 5E-74
 Identities = 111/119 (93%), Positives = 116/119 (97%), Gaps = 0/119 (0%)

Query  25   NKKQQSHSARAGLQFPCGRVKRFLKQNTQQKMRVGAKAAVYVTAVLEYLTAEVLELAGNA  84
            +KKQQSHS++AGLQFPCGRVKRFLK NTQ KMRVGAKAAVYVTAVLEYLTAEVLELAGNA
Sbjct  23   SKKQQSHSSKAGLQFPCGRVKRFLKNNTQNKMRVGAKAAVYVTAVLEYLTAEVLELAGNA  82

Query  85   AKDLKVKRITPRHLQLAIRGDEELDTLIRATIAYGGVLPHINRALLLKVEQKKKAKALE  143
```

```
            AKDLKVKRITPRHLQLAIRGDEELDTLIRATIA+GGVLPHINRALLLKVEQKKK KA+E
Sbjct  83   AKDLKVKRITPRHLQLAIRGDEELDTLIRATIAFGGVLPHINRALLLKVEQKKKNKAIE  141


>XP_016646064.1| hypothetical protein SAPIO_CDS1167 [Scedosporium apiospermum]
 gb|KEZ46265.1| hypothetical protein SAPIO_CDS1167 [Scedosporium apiospermum]
          Length=142

 Score = 226.9 bits (577),  Expect = 5E-74
 Identities = 112/116 (96%), Positives = 113/116 (97%), Gaps = 0/116 (0%)

Query  26   KKQQSHSARAGLQFPCGRVKRFLKQNTQQKMRVGAKAAVYVTAVLEYLTAEVLELAGNAA  85
            KKQQSHSARAGLQFPCGRVKRFLKQNTQ KMRVGAKAAVYVTAVLEYLTAEVLELAGNAA
Sbjct  24   KKQQSHSARAGLQFPCGRVKRFLKQNTQNKMRVGAKAAVYVTAVLEYLTAEVLELAGNAA  83

Query  86   KDLKVKRITPRHLQLAIRGDEELDTLIRATIAYGGVLPHINRALLLKVEQKKKAKA  141
            KDLKVKRITPRHLQLAIRGDEELDTLIRATIA+GGVLPHINRALLLKVEQKKK  A
Sbjct  84   KDLKVKRITPRHLQLAIRGDEELDTLIRATIAFGGVLPHINRALLLKVEQKKKKAA  139


>XP_017994961.1| Histone H2A.Z [Phialophora attinorum]
 gb|KPI34998.1| Histone H2A.Z [Phialophora attinorum]
          Length=140

 Score = 225.7 bits (574),  Expect = 1E-73
 Identities = 110/122 (90%), Positives = 116/122 (95%), Gaps = 0/122 (0%)

Query  22   EGANKKQQSHSARAGLQFPCGRVKRFLKQNTQQKMRVGAKAAVYVTAVLEYLTAEVLELA  81
            +  +K Q+SHSA+AGLQFPCGRVKRFLK NTQ KMRVGAKAAVYVTAVLEYLTAEVLELA
Sbjct  18   DSGSKNQKSHSAKAGLQFPCGRVKRFLKNNTQNKMRVGAKAAVYVTAVLEYLTAEVLELA  77

Query  82   GNAAKDLKVKRITPRHLQLAIRGDEELDTLIRATIAYGGVLPHINRALLLKVEQKKKAKA  141
            GNAAKDLKVKRITPRHLQLAIRGDEELDTLIRATIA+GGVLPHINRALLLKVEQKKK KA
Sbjct  78   GNAAKDLKVKRITPRHLQLAIRGDEELDTLIRATIAFGGVLPHINRALLLKVEQKKKNKA  137
```

```
Query  142  LE  143
            +E
Sbjct  138  IE  139


>XP_030993423.1| uncharacterized protein E0L32_007691 [Phialemoniopsis curvata]
 gb|TPX11712.1| hypothetical protein E0L32_007691 [Phialemoniopsis curvata]
          Length=142

 Score = 225.3 bits (573),  Expect = 2E-73
 Identities = 112/119 (94%), Positives = 114/119 (96%), Gaps = 0/119 (0%)

Query  26   KKQQSHSARAGLQFPCGRVKRFLKQNTQQKMRVGAKAAVYVTAVLEYLTAEVLELAGNAA  85
            KKQQSHSARAGLQFPCGRVKRFLK NTQ KMRVGAKAAVYVTAVLEYLTAEVLELAGNAA
Sbjct  24   KKQQSHSARAGLQFPCGRVKRFLKANTQNKMRVGAKAAVYVTAVLEYLTAEVLELAGNAA  83

Query  86   KDLKVKRITPRHLQLAIRGDEELDTLIRATIAYGGVLPHINRALLLKVEQKKKAKALEG  144
            KDLKVKRITPRHLQLAIRGDEELDTLIRATIA+GGVLPHINRALLLKVEQKKK +A E
Sbjct  84   KDLKVKRITPRHLQLAIRGDEELDTLIRATIAFGGVLPHINRALLLKVEQKKKKQAAEA  142


>XP_001597856.1| histone H2A [Sclerotinia sclerotiorum 1980 UF-70]
 gb|APA05626.1| hypothetical protein sscle_01g003960 [Sclerotinia sclerotiorum 1980 UF-70]
 gb|EDN97124.1| histone H2A [Sclerotinia sclerotiorum 1980 UF-70]
          Length=142

 Score = 224.6 bits (571),  Expect = 4E-73
 Identities = 110/117 (94%), Positives = 113/117 (97%), Gaps = 0/117 (0%)

Query  25   NKKQQSHSARAGLQFPCGRVKRFLKQNTQQKMRVGAKAAVYVTAVLEYLTAEVLELAGNA  84
            NKKQQSHS++AGLQFPCGRVKRFLK NTQ KMRVGAKAAVYVTAVLEYLTAEVLELAGNA
Sbjct  23   NKKQQSHSSKAGLQFPCGRVKRFLKNNTQNKMRVGAKAAVYVTAVLEYLTAEVLELAGNA  82
```

```
Query  85   AKDLKVKRITPRHLQLAIRGDEELDTLIRATIAYGGVLPHINRALLLKVEQKKKAKA  141
            AKDLKVKRITPRHLQLAIRGDEELDTLIRATIA+GGVLPHINRALLLKVEQKKK  A
Sbjct  83   AKDLKVKRITPRHLQLAIRGDEELDTLIRATIAFGGVLPHINRALLLKVEQKKKKTA  139


>XP_018146336.1| histone H2A [Pochonia chlamydosporia 170]
 gb|OAQ69799.1| histone H2A [Pochonia chlamydosporia 170]
          Length=143

 Score = 224.6 bits (571),  Expect = 4E-73
 Identities = 113/121 (93%), Positives = 117/121 (97%), Gaps = 1/121 (1%)

Query  25   NKKQQSHSARAGLQFPCGRVKRFLKQNTQQKMRVGAKAAVYVTAVLEYLTAEVLELAGNA  84
            +KKQQSHSARAGLQFPCGRVKRFLK NTQ KMRVGAKAAVYVTAVLEYLTAEVLELAGNA
Sbjct  23   HKKQQSHSARAGLQFPCGRVKRFLKANTQNKMRVGAKAAVYVTAVLEYLTAEVLELAGNA  82

Query  85   AKDLKVKRITPRHLQLAIRGDEELDTLIRATIAYGGVLPHINRALLLKVEQ-KKKAKALE  143
            AKDLKVKRITPRHLQLAIRGDEELDTLIRATIA+GGVLPHINRALLLKVEQ KKKA+A+E
Sbjct  83   AKDLKVKRITPRHLQLAIRGDEELDTLIRATIAFGGVLPHINRALLLKVEQKKKKAQAIE  142

Query  144  G  144

Sbjct  143  A  143


>XP_013283629.1| histone H2A.Z [Fonsecaea pedrosoi CBS 271.37]
 ref|XP_016626579.1| histone H2A.Z [Fonsecaea multimorphosa CBS 102226]
 ref|XP_022503217.1| histone H2A.Z [Fonsecaea nubica]
 ref|XP_022514661.1| histone H2A.Z [Fonsecaea monophora]
 gb|OAL19572.1| histone H2A.Z [Fonsecaea multimorphosa]
 gb|KIW79821.1| histone H2A.Z [Fonsecaea pedrosoi CBS 271.37]
 gb|KIX92456.1| histone H2A.Z [Fonsecaea multimorphosa CBS 102226]
 gb|OAG42709.1| histone H2A.Z [Fonsecaea monophora]
 gb|OAL38205.1| histone H2A.Z [Fonsecaea nubica]
```

```
          Length=139

 Score = 224.2 bits (570),  Expect = 4E-73
 Identities = 109/120 (90%), Positives = 114/120 (95%), Gaps = 0/120 (0%)

Query  22   EGANKKQQSHSARAGLQFPCGRVKRFLKQNTQQKMRVGAKAAVYVTAVLEYLTAEVLELA  81
            +   K Q+SHSA+AGLQFPCGRVKRFLK NTQ KMRVGAKAAVYVTAVLEYLTAEVLELA
Sbjct  18   DSTTKTQKSHSAKAGLQFPCGRVKRFLKNNTQNKMRVGAKAAVYVTAVLEYLTAEVLELA  77

Query  82   GNAAKDLKVKRITPRHLQLAIRGDEELDTLIRATIAYGGVLPHINRALLLKVEQKKKAKA  141
            GNAAKDLKVKRITPRHLQLAIRGDEELDTLIRATIA+GGVLPHINRALLLKVEQKKK+KA
Sbjct  78   GNAAKDLKVKRITPRHLQLAIRGDEELDTLIRATIAFGGVLPHINRALLLKVEQKKKSKA  137


>XP_018694917.1| histone H2A.Z [Fonsecaea erecta]
 gb|OAP61550.1| histone H2A.Z [Fonsecaea erecta]
          Length=139

 Score = 224.2 bits (570),  Expect = 5E-73
 Identities = 109/119 (91%), Positives = 114/119 (96%), Gaps = 0/119 (0%)

Query  22   EGANKKQQSHSARAGLQFPCGRVKRFLKQNTQQKMRVGAKAAVYVTAVLEYLTAEVLELA  81
            + A K Q+SHSA+AGLQFPCGRVKRFLK NTQ KMRVGAKAAVYVTAVLEYLTAEVLELA
Sbjct  18   DSATKTQKSHSAKAGLQFPCGRVKRFLKNNTQNKMRVGAKAAVYVTAVLEYLTAEVLELA  77

Query  82   GNAAKDLKVKRITPRHLQLAIRGDEELDTLIRATIAYGGVLPHINRALLLKVEQKKKAK  140
            GNAAKDLKVKRITPRHLQLAIRGDEELDTLIRATIA+GGVLPHINRALLLKVEQKKK+K
Sbjct  78   GNAAKDLKVKRITPRHLQLAIRGDEELDTLIRATIAFGGVLPHINRALLLKVEQKKKSK  136


>XP_007728785.1| histone H2A.Z [Capronia epimyces CBS 606.96]
 gb|EXJ91895.1| histone H2A.Z [Capronia epimyces CBS 606.96]
          Length=139
```

```
 Score = 223.8 bits (569),  Expect = 6E-73
 Identities = 109/116 (93%), Positives = 113/116 (97%), Gaps = 0/116 (0%)

Query  26   KKQQSHSARAGLQFPCGRVKRFLKQNTQQKMRVGAKAAVYVTAVLEYLTAEVLELAGNAA  85
            K Q+SHSA+AGLQFPCGRVKRFLK NTQ KMRVGAKAAVYVTAVLEYLTAEVLELAGNAA
Sbjct  22   KSQKSHSAKAGLQFPCGRVKRFLKNNTQNKMRVGAKAAVYVTAVLEYLTAEVLELAGNAA  81

Query  86   KDLKVKRITPRHLQLAIRGDEELDTLIRATIAYGGVLPHINRALLLKVEQKKKAKA  141
            KDLKVKRITPRHLQLAIRGDEELDTLIRATIA+GGVLPHINRALLLKVEQKKK+KA
Sbjct  82   KDLKVKRITPRHLQLAIRGDEELDTLIRATIAFGGVLPHINRALLLKVEQKKKSKA  137


>XP_016232314.1| histone H2A.Z [Exophiala spinifera]
 gb|KIW12098.1| histone H2A.Z [Exophiala spinifera]
          Length=138

 Score = 223.8 bits (569),  Expect = 6E-73
 Identities = 109/120 (90%), Positives = 115/120 (96%), Gaps = 0/120 (0%)

Query  22   EGANKKQQSHSARAGLQFPCGRVKRFLKQNTQQKMRVGAKAAVYVTAVLEYLTAEVLELA  81
            + + K Q+SHSA+AGLQFPCGRVKRFLK NTQ KMRVGAKAAVYVTAVLEYLTAEVLELA
Sbjct  18   DSSVKSQKSHSAKAGLQFPCGRVKRFLKNNTQNKMRVGAKAAVYVTAVLEYLTAEVLELA  77

Query  82   GNAAKDLKVKRITPRHLQLAIRGDEELDTLIRATIAYGGVLPHINRALLLKVEQKKKAKA  141
            GNAAKDLKVKRITPRHLQLAIRGDEELDTLIRATIA+GGVLPHINRALLLKVEQKKK+KA
Sbjct  78   GNAAKDLKVKRITPRHLQLAIRGDEELDTLIRATIAFGGVLPHINRALLLKVEQKKKSKA  137


>XP_018063296.1| histone-fold-containing protein [Phialocephala scopiformis]
 gb|KUJ08941.1| histone-fold-containing protein [Phialocephala scopiformis]
          Length=141

 Score = 223.4 bits (568),  Expect = 1E-72
 Identities = 109/117 (93%), Positives = 113/117 (97%), Gaps = 0/117 (0%)
```

```
Query  24   ANKKQQSHSARAGLQFPCGRVKRFLKQNTQQKMRVGAKAAVYVTAVLEYLTAEVLELAGN  83
            + KKQQSHS++AGLQFPCGRVKRFLK NTQ KMRVGAKAAVYVTAVLEYLTAEVLELAGN
Sbjct  22   SGKKQQSHSSKAGLQFPCGRVKRFLKNNTQNKMRVGAKAAVYVTAVLEYLTAEVLELAGN  81

Query  84   AAKDLKVKRITPRHLQLAIRGDEELDTLIRATIAYGGVLPHINRALLLKVEQKKKAK  140
            AAKDLKVKRITPRHLQLAIRGDEELDTLIRATIA+GGVLPHINRALLLKVEQKKK K
Sbjct  82   AAKDLKVKRITPRHLQLAIRGDEELDTLIRATIAFGGVLPHINRALLLKVEQKKKTK  138


>XP_028462876.1| histone H2A.Z [Sodiomyces alkalinus F11]
 gb|ROT35070.1| histone H2A.Z [Sodiomyces alkalinus F11]
          Length=141

 Score = 223.0 bits (567),  Expect = 2E-72
 Identities = 111/118 (94%), Positives = 113/118 (96%), Gaps = 0/118 (0%)

Query  26   KKQQSHSARAGLQFPCGRVKRFLKQNTQQKMRVGAKAAVYVTAVLEYLTAEVLELAGNAA  85
            KK QSHSARAGLQFPCGRVKRFLK NTQ KMRVGAKAAVY TAVLEYLTAEVLELAGNAA
Sbjct  23   KKPQSHSARAGLQFPCGRVKRFLKANTQGKMRVGAKAAVYTTAVLEYLTAEVLELAGNAA  82

Query  86   KDLKVKRITPRHLQLAIRGDEELDTLIRATIAYGGVLPHINRALLLKVEQKKKAKALE  143
            KDLKVKRITPRHLQLAIRGDEELDTLIRATIA+GGVLPHINRALLLKVEQKKKAK +E
Sbjct  83   KDLKVKRITPRHLQLAIRGDEELDTLIRATIAFGGVLPHINRALLLKVEQKKKAKQIE  140


>XP_024733061.1| histone-fold-containing protein, partial [Hyaloscypha bicolor E]
 gb|PMD38620.1| histone-fold-containing protein, partial [Hyaloscypha variabilis F]
 gb|PMD56157.1| histone-fold-containing protein, partial [Hyaloscypha bicolor E]
          Length=127

 Score = 222.2 bits (565),  Expect = 2E-72
 Identities = 109/115 (94%), Positives = 112/115 (97%), Gaps = 0/115 (0%)
```

```
Query  26   KKQQSHSARAGLQFPCGRVKRFLKQNTQQKMRVGAKAAVYVTAVLEYLTAEVLELAGNAA  85
            KKQQSHS++AGLQFPCGRVKRFLK NTQ KMRVGAKAAVYVTAVLEYLTAEVLELAGNAA
Sbjct  13   KKQQSHSSKAGLQFPCGRVKRFLKNNTQNKMRVGAKAAVYVTAVLEYLTAEVLELAGNAA  72

Query  86   KDLKVKRITPRHLQLAIRGDEELDTLIRATIAYGGVLPHINRALLLKVEQKKKAK  140
            KDLKVKRITPRHLQLAIRGDEELDTLIRATIA+GGVLPHINRALLLKVEQKKK K
Sbjct  73   KDLKVKRITPRHLQLAIRGDEELDTLIRATIAFGGVLPHINRALLLKVEQKKKNK  127


>XP_016224592.1| histone H2A.Z [Exophiala mesophila]
 gb|KIV93018.1| histone H2A.Z [Exophiala mesophila]
 gb|RVX71260.1| Histone H2A.Z [Exophiala mesophila]
          Length=139

 Score = 222.6 bits (566),  Expect = 2E-72
 Identities = 108/119 (90%), Positives = 114/119 (96%), Gaps = 0/119 (0%)

Query  22   EGANKKQQSHSARAGLQFPCGRVKRFLKQNTQQKMRVGAKAAVYVTAVLEYLTAEVLELA  81
            + + K Q+SHSA+AGLQFPCGRVKRFLK NTQ KMRVGAKAAVYVTAVLEYLTAEVLELA
Sbjct  18   DSSVKSQKSHSAKAGLQFPCGRVKRFLKNNTQNKMRVGAKAAVYVTAVLEYLTAEVLELA  77

Query  82   GNAAKDLKVKRITPRHLQLAIRGDEELDTLIRATIAYGGVLPHINRALLLKVEQKKKAK  140
            GNAAKDLKVKRITPRHLQLAIRGDEELDTLIRATIA+GGVLPHINRALLLKVEQKKK+K
Sbjct  78   GNAAKDLKVKRITPRHLQLAIRGDEELDTLIRATIAFGGVLPHINRALLLKVEQKKKSK  136


>XP_007293349.1| histone H2A [Marssonina brunnea f. sp. 'multigermtubi' MB_m1]
 gb|EKD16166.1| histone H2A [Marssonina brunnea f. sp. 'multigermtubi' MB_m1]
          Length=142

 Score = 222.2 bits (565),  Expect = 3E-72
 Identities = 108/115 (93%), Positives = 113/115 (98%), Gaps = 0/115 (0%)

Query  24   ANKKQQSHSARAGLQFPCGRVKRFLKQNTQQKMRVGAKAAVYVTAVLEYLTAEVLELAGN  83
```

```
            A+KKQQSHS++AGLQFPCGRVKRFLK NTQ KMRVGAKAAVYVTAVLEYLTAEVLELAGN
Sbjct  22   ASKKQQSHSSKAGLQFPCGRVKRFLKNNTQNKMRVGAKAAVYVTAVLEYLTAEVLELAGN  81

Query  84   AAKDLKVKRITPRHLQLAIRGDEELDTLIRATIAYGGVLPHINRALLLKVEQKKK  138
            AAKDLKVKRITPRHLQLAIRGDEELD+LIRATIA+GGVLPHINRALLLKVEQKKK
Sbjct  82   AAKDLKVKRITPRHLQLAIRGDEELDSLIRATIAFGGVLPHINRALLLKVEQKKK  136


>XP_013266185.1| histone H2A.Z [Exophiala aquamarina CBS 119918]
 gb|KEF63595.1| histone H2A.Z [Exophiala aquamarina CBS 119918]
          Length=139

 Score = 221.9 bits (564),  Expect = 3E-72
 Identities = 108/119 (90%), Positives = 113/119 (95%), Gaps = 0/119 (0%)

Query  22   EGANKKQQSHSARAGLQFPCGRVKRFLKQNTQQKMRVGAKAAVYVTAVLEYLTAEVLELA  81
            + + K Q+SHSA+AGLQFPCGRVKRFLK NTQ KMRVGAKAAVYVTAVLEYLTAEVLELA
Sbjct  18   DSSTKTQKSHSAKAGLQFPCGRVKRFLKNNTQNKMRVGAKAAVYVTAVLEYLTAEVLELA  77

Query  82   GNAAKDLKVKRITPRHLQLAIRGDEELDTLIRATIAYGGVLPHINRALLLKVEQKKKAK  140
            GNAAKDLKVKRITPRHLQLAIRGDEELDTLIRATIA+GGVLPHINRALLLKVEQKKK K
Sbjct  78   GNAAKDLKVKRITPRHLQLAIRGDEELDTLIRATIAFGGVLPHINRALLLKVEQKKKNK  136


>XP_031001079.1| Histone H2A.Z [Lachnellula hyalina]
 gb|TVY22291.1| Histone H2A.Z [Lachnellula hyalina]
 gb|TVY33519.1| Histone H2A.Z [Lachnellula occidentalis]
 gb|TVY38472.1| Histone H2A.Z [Lachnellula subtilissima]
          Length=140

 Score = 221.1 bits (562),  Expect = 9E-72
 Identities = 108/113 (95%), Positives = 111/113 (98%), Gaps = 0/113 (0%)

Query  26   KKQQSHSARAGLQFPCGRVKRFLKQNTQQKMRVGAKAAVYVTAVLEYLTAEVLELAGNAA  85
```

```
            KKQQSHS++AGLQFPCGRVKRFLK NTQ KMRVGAKAAVYVTAVLEYLTAEVLELAGNAA
Sbjct  24   KKQQSHSSKAGLQFPCGRVKRFLKNNTQNKMRVGAKAAVYVTAVLEYLTAEVLELAGNAA  83

Query  86   KDLKVKRITPRHLQLAIRGDEELDTLIRATIAYGGVLPHINRALLLKVEQKKK  138
            KDLKVKRITPRHLQLAIRGDEELDTLIRATIA+GGVLPHINRALLLKVEQKKK
Sbjct  84   KDLKVKRITPRHLQLAIRGDEELDTLIRATIAFGGVLPHINRALLLKVEQKKK  136


>XP_020061199.1| uncharacterized protein ASPACDRAFT_49223 [Aspergillus aculeatus ATCC 16872]
 ref|XP_025445879.1| histone-fold-containing protein [Aspergillus brunneoviolaceus CBS 621.78]
 ref|XP_025491701.1| histone-fold-containing protein [Aspergillus uvarum CBS 121591]
 ref|XP_025506846.1| histone-fold-containing protein [Aspergillus aculeatinus CBS 121060]
 ref|XP_025550306.1| histone-fold-containing protein [Aspergillus homomorphus CBS 101889]
 gb|PYI21471.1| histone-fold-containing protein [Aspergillus violaceofuscus CBS 115571]
 gb|PYI27189.1| histone-fold-containing protein [Aspergillus indologenus CBS 114.80]
 gb|RAK73827.1| histone-fold-containing protein [Aspergillus fijiensis CBS 313.89]
 gb|OJK04860.1| hypothetical protein ASPACDRAFT_49223 [Aspergillus aculeatus ATCC 16872]
 gb|PYH81501.1| histone-fold-containing protein [Aspergillus uvarum CBS 121591]
          Length=138

 Score = 219.9 bits (559),  Expect = 2E-71
 Identities = 108/119 (90%), Positives = 113/119 (95%), Gaps = 0/119 (0%)

Query  22   EGANKKQQSHSARAGLQFPCGRVKRFLKQNTQQKMRVGAKAAVYVTAVLEYLTAEVLELA  81
            + A K Q+SHSA+AGLQFPCGRVKRFLK NTQ KMRVGAKAAVYVTAVLEYLTAEVLELA
Sbjct  17   DSAGKSQKSHSAKAGLQFPCGRVKRFLKNNTQNKMRVGAKAAVYVTAVLEYLTAEVLELA  76

Query  82   GNAAKDLKVKRITPRHLQLAIRGDEELDTLIRATIAYGGVLPHINRALLLKVEQKKKAK  140
            GNAAKDLKVKRITPRHLQLAIRGDEELDTLIRATIA+GGVLP INRALLLKVEQKKK+K
Sbjct  77   GNAAKDLKVKRITPRHLQLAIRGDEELDTLIRATIAFGGVLPRINRALLLKVEQKKKSK  135


>XP_002151102.1| histone H2A [Talaromyces marneffei ATCC 18224]
 gb|KAE8549119.1| hypothetical protein EYB25_007634 [Talaromyces marneffei]
```

```
 gb|KAF3393457.1| Histone H2A.Z [Penicillium pinophilum]
 gb|KFX48366.1| Histone H2A.Z [Talaromyces marneffei PM1]
 gb|PCH00541.1| Histone-fold [Penicillium sp. 'occitanis']
 dbj|GAM41843.1| histone [Talaromyces cellulolyticus]
          Length=139

 Score = 219.9 bits (559),  Expect = 3E-71
 Identities = 109/119 (91%), Positives = 112/119 (94%), Gaps = 0/119 (0%)

Query  22   EGANKKQQSHSARAGLQFPCGRVKRFLKQNTQQKMRVGAKAAVYVTAVLEYLTAEVLELA  81
            + A K Q+SHSA+AGLQFPCGRVKRFLK NTQ KMRVGAKAAVYVTAVLEYLTAEVLELA
Sbjct  18   DAAGKTQKSHSAKAGLQFPCGRVKRFLKNNTQNKMRVGAKAAVYVTAVLEYLTAEVLELA  77

Query  82   GNAAKDLKVKRITPRHLQLAIRGDEELDTLIRATIAYGGVLPHINRALLLKVEQKKKAK  140
            GNAAKDLKVKRITPRHLQLAIRGDEELDTLIRATIAYGGVLP INRALLLKVEQKKK K
Sbjct  78   GNAAKDLKVKRITPRHLQLAIRGDEELDTLIRATIAYGGVLPRINRALLLKVEQKKKGK  136


>XP_002341942.1| histone H2A [Talaromyces stipitatus ATCC 10500]
 gb|EED24555.1| histone H2A [Talaromyces stipitatus ATCC 10500]
          Length=139

 Score = 219.2 bits (557),  Expect = 4E-71
 Identities = 108/119 (90%), Positives = 112/119 (94%), Gaps = 0/119 (0%)

Query  22   EGANKKQQSHSARAGLQFPCGRVKRFLKQNTQQKMRVGAKAAVYVTAVLEYLTAEVLELA  81
            + A K Q+SHSA+AGLQFPCGRVKRFLK NTQ KMRVGAKAAVYVTAVLEYLTAEVLELA
Sbjct  18   DAAGKSQKSHSAKAGLQFPCGRVKRFLKNNTQNKMRVGAKAAVYVTAVLEYLTAEVLELA  77

Query  82   GNAAKDLKVKRITPRHLQLAIRGDEELDTLIRATIAYGGVLPHINRALLLKVEQKKKAK  140
            GNAAKDLKVKRITPRHLQLAIRGDEELDTLIRATIA+GGVLP INRALLLKVEQKKK K
Sbjct  78   GNAAKDLKVKRITPRHLQLAIRGDEELDTLIRATIAFGGVLPRINRALLLKVEQKKKGK  136
```

```
>XP_020120058.1| Histone H2A.Z [Talaromyces atroroseus]
 gb|OKL59937.1| Histone H2A.Z [Talaromyces atroroseus]
          Length=139

 Score = 218.8 bits (556),  Expect = 6E-71
 Identities = 108/119 (90%), Positives = 112/119 (94%), Gaps = 0/119 (0%)

Query  22   EGANKKQQSHSARAGLQFPCGRVKRFLKQNTQQKMRVGAKAAVYVTAVLEYLTAEVLELA  81
            + A K Q+SHSA+AGLQFPCGRVKRFLK NTQ KMRVGAKAAVYVTAVLEYLTAEVLELA
Sbjct  18   DAAGKNQKSHSAKAGLQFPCGRVKRFLKNNTQNKMRVGAKAAVYVTAVLEYLTAEVLELA  77

Query  82   GNAAKDLKVKRITPRHLQLAIRGDEELDTLIRATIAYGGVLPHINRALLLKVEQKKKAK  140
            GNAAKDLKVKRITPRHLQLAIRGDEELDTLIRATIA+GGVLP INRALLLKVEQKKK K
Sbjct  78   GNAAKDLKVKRITPRHLQLAIRGDEELDTLIRATIAFGGVLPRINRALLLKVEQKKKGK  136


>XP_024685068.1| histone-fold-containing protein [Aspergillus novofumigatus IBT 16806]
 ref|XP_033411515.1| histone-fold-containing protein [Aspergillus lentulus]
 gb|KAF4212458.1| hypothetical protein CNMCM5878_001139 [Aspergillus fumigatiaffinis]
 gb|RHZ70010.1| histone H2A.Z [Aspergillus turcosus]
 dbj|GAO84568.1| histone H2A.Z [Aspergillus udagawae]
 gb|KAF4151784.1| hypothetical protein CNMCM6069_003052 [Aspergillus lentulus]
 gb|KAF4163116.1| hypothetical protein CNMCM6936_001253 [Aspergillus lentulus]
          Length=138

 Score = 218.8 bits (556),  Expect = 6E-71
 Identities = 108/120 (90%), Positives = 113/120 (94%), Gaps = 0/120 (0%)

Query  22   EGANKKQQSHSARAGLQFPCGRVKRFLKQNTQQKMRVGAKAAVYVTAVLEYLTAEVLELA  81
            + A K Q+SHSA+AGLQFPCGRVKRFLK NTQ KMRVGAKAAVYVTAVLEYLTAEVLELA
Sbjct  17   DAAGKTQKSHSAKAGLQFPCGRVKRFLKNNTQNKMRVGAKAAVYVTAVLEYLTAEVLELA  76

Query  82   GNAAKDLKVKRITPRHLQLAIRGDEELDTLIRATIAYGGVLPHINRALLLKVEQKKKAKA  141
            GNAAKDLKVKRITPRHLQLAIRGDEELDTLIRATIA+GGVLP INRALLLKVEQKKK K+
```

```
Sbjct  77   GNAAKDLKVKRITPRHLQLAIRGDEELDTLIRATIAFGGVLPRINRALLLKVEQKKKNKS  136


>XP_001266338.1| histone H2A [Aspergillus fischeri NRRL 181]
 ref|XP_748147.1| histone H2A [Aspergillus fumigatus Af293]
 sp|A1D0C1.1| RecName: Full=Histone H2A.Z [Aspergillus fischeri NRRL 181]
 sp|Q4WE68.1| RecName: Full=Histone H2A.Z [Aspergillus fumigatus Af293]
 gb|EDP51045.1| histone H2A [Aspergillus fumigatus A1163]
 gb|KAF4262835.1| hypothetical protein CNMCM8057_001248 [Aspergillus fumigatus]
 gb|KEY78711.1| histone H2A [Aspergillus fumigatus var. RP-2014]
 gb|KMK58960.1| histone H2A [Aspergillus fumigatus Z5]
 gb|EAL86109.1| histone H2A [Aspergillus fumigatus Af293]
          Length=138

 Score = 218.8 bits (556),  Expect = 6E-71
 Identities = 108/120 (90%), Positives = 113/120 (94%), Gaps = 0/120 (0%)

Query  22   EGANKKQQSHSARAGLQFPCGRVKRFLKQNTQQKMRVGAKAAVYVTAVLEYLTAEVLELA  81
            + A K Q+SHSA+AGLQFPCGRVKRFLK NTQ KMRVGAKAAVYVTAVLEYLTAEVLELA
Sbjct  17   DAAGKTQKSHSAKAGLQFPCGRVKRFLKNNTQNKMRVGAKAAVYVTAVLEYLTAEVLELA  76

Query  82   GNAAKDLKVKRITPRHLQLAIRGDEELDTLIRATIAYGGVLPHINRALLLKVEQKKKAKA  141
            GNAAKDLKVKRITPRHLQLAIRGDEELDTLIRATIA+GGVLP INRALLLKVEQKKK K+
Sbjct  77   GNAAKDLKVKRITPRHLQLAIRGDEELDTLIRATIAFGGVLPRINRALLLKVEQKKKNKS  136


>XP_035341716.1| uncharacterized protein TRUGW13939_02632 [Talaromyces rugulosus]
 gb|QKX55538.1| hypothetical protein TRUGW13939_02632 [Talaromyces rugulosus]
          Length=139

 Score = 218.8 bits (556),  Expect = 6E-71
 Identities = 109/119 (91%), Positives = 112/119 (94%), Gaps = 0/119 (0%)

Query  22   EGANKKQQSHSARAGLQFPCGRVKRFLKQNTQQKMRVGAKAAVYVTAVLEYLTAEVLELA  81
```

```
            + A K Q+SHSA+AGLQFPCGRVKRFLK NTQ KMRVGAKAAVYVTAVLEYLTAEVLELA
Sbjct  18   DAAGKAQKSHSAKAGLQFPCGRVKRFLKNNTQNKMRVGAKAAVYVTAVLEYLTAEVLELA  77

Query  82   GNAAKDLKVKRITPRHLQLAIRGDEELDTLIRATIAYGGVLPHINRALLLKVEQKKKAK  140
            GNAAKDLKVKRITPRHLQLAIRGDEELDTLIRATIAYGGVLP INRALLLKVEQKKK K
Sbjct  78   GNAAKDLKVKRITPRHLQLAIRGDEELDTLIRATIAYGGVLPRINRALLLKVEQKKKNK  136


>XP_022578503.1| hypothetical protein ASPZODRAFT_153973 [Penicilliopsis zonata CBS 506.65]
 gb|OJJ43993.1| hypothetical protein ASPZODRAFT_153973 [Penicilliopsis zonata CBS 506.65]
          Length=138

 Score = 218.8 bits (556),  Expect = 7E-71
 Identities = 110/123 (89%), Positives = 115/123 (93%), Gaps = 1/123 (1%)

Query  22   EGANKKQQSHSARAGLQFPCGRVKRFLKQNTQQKMRVGAKAAVYVTAVLEYLTAEVLELA  81
            + A K Q+SHSA+AGLQFPCGRVKRFLK NTQ KMRVGAKAAVYVTAVLEYLTAEVLELA
Sbjct  17   DAAGKTQKSHSAKAGLQFPCGRVKRFLKNNTQNKMRVGAKAAVYVTAVLEYLTAEVLELA  76

Query  82   GNAAKDLKVKRITPRHLQLAIRGDEELDTLIRATIAYGGVLPHINRALLLKVEQKKKAKA  141
            GNAAKDLKVKRITPRHLQLAIRGDEELDTLIRATIA+GGVLP INRALLLKVEQKKK K
Sbjct  77   GNAAKDLKVKRITPRHLQLAIRGDEELDTLIRATIAFGGVLPRINRALLLKVEQKKKNK-  135

Query  142  LEG  144
            +EG
Sbjct  136  IEG  137


>XP_001820387.1| unnamed protein product [Aspergillus oryzae RIB40]
 ref|XP_002374019.1| histone H2A [Aspergillus flavus NRRL3357]
 ref|XP_015412489.1| histone H2A.Z [Aspergillus nomiae NRRL 13137]
 ref|XP_022390191.1| histone H2A.Z [Aspergillus bombycis]
 ref|XP_031900224.1| histone H2A.Z [Aspergillus alliaceus]
 ref|XP_031943341.1| histone H2A.Z [Aspergillus pseudonomius]
```

```
 sp|Q2UJ80.1| RecName: Full=Histone H2A.Z [Aspergillus oryzae RIB40]
 gb|EIT83564.1| histone 2A [Aspergillus oryzae 3.042]
 gb|KAB8075793.1| histone H2A.Z [Aspergillus leporis]
 gb|KAB8211663.1| histone H2A.Z [Aspergillus parasiticus]
 gb|KAB8214781.1| histone H2A.Z [Aspergillus novoparasiticus]
 gb|KAB8246268.1| histone H2A.Z [Aspergillus flavus]
 gb|KAB8279261.1| histone H2A.Z [Aspergillus minisclerotigenes]
 gb|KAE8134717.1| histone H2A.Z [Aspergillus pseudotamarii]
 gb|KAE8167016.1| histone H2A.Z [Aspergillus tamarii]
 gb|KAE8311648.1| histone H2A.Z [Aspergillus transmontanensis]
 gb|KAE8326449.1| histone H2A.Z [Aspergillus sergii]
 gb|KAE8344758.1| histone H2A.Z [Aspergillus arachidicola]
 gb|KAE8383772.1| histone H2A.Z [Aspergillus bertholletiae]
 gb|KAE8412900.1| histone H2A.Z [Aspergillus pseudocaelatus]
 gb|KAF5857362.1| histone H2A.Z [Aspergillus sp. CLMG-2019a]
 gb|KDE78059.1| histone 2A [Aspergillus oryzae 100-8]
 gb|KJK65508.1| hypothetical protein P875_00010197 [Aspergillus parasiticus SU-1]
 gb|KOC07557.1| histone H2A [Aspergillus flavus AF70]
 gb|OOO09743.1| Histone core domain protein [Aspergillus oryzae]
          Length=138

 Score = 218.4 bits (555),  Expect = 9E-71
 Identities = 108/119 (90%), Positives = 112/119 (94%), Gaps = 0/119 (0%)

Query  22   EGANKKQQSHSARAGLQFPCGRVKRFLKQNTQQKMRVGAKAAVYVTAVLEYLTAEVLELA  81
            + A K Q+SHSA+AGLQFPCGRVKRFLK NTQ KMRVGAKAAVYVTAVLEYLTAEVLELA
Sbjct  17   DSAGKAQKSHSAKAGLQFPCGRVKRFLKNNTQNKMRVGAKAAVYVTAVLEYLTAEVLELA  76

Query  82   GNAAKDLKVKRITPRHLQLAIRGDEELDTLIRATIAYGGVLPHINRALLLKVEQKKKAK  140
            GNAAKDLKVKRITPRHLQLAIRGDEELDTLIRATIA+GGVLP INRALLLKVEQKKK K
Sbjct  77   GNAAKDLKVKRITPRHLQLAIRGDEELDTLIRATIAFGGVLPRINRALLLKVEQKKKNK  135


>XP_016760042.1| histone H2A [Sphaerulina musiva SO2202]
```

```
 gb|EMF11921.1| histone H2A [Sphaerulina musiva SO2202]
         Length=140

 Score = 218.4 bits (555),  Expect = 1E-70
 Identities = 107/120 (89%), Positives = 112/120 (93%), Gaps = 0/120 (0%)

Query  22   EGANKKQQSHSARAGLQFPCGRVKRFLKQNTQQKMRVGAKAAVYVTAVLEYLTAEVLELA  81
            + + K Q+SHSA+AGLQFPCGRVKRFLK  TQ KMRVGAKAAVYVTAVLEYLTAEVLELA
Sbjct  19   DNSGKSQKSHSAKAGLQFPCGRVKRFLKSQTQNKMRVGAKAAVYVTAVLEYLTAEVLELA  78

Query  82   GNAAKDLKVKRITPRHLQLAIRGDEELDTLIRATIAYGGVLPHINRALLLKVEQKKKAKA  141
            GNAAKDLKVKRITPRHLQLAIRGDEELDTLIRATIA+GGVLPHINRALLLKVEQKK  KA
Sbjct  79   GNAAKDLKVKRITPRHLQLAIRGDEELDTLIRATIAFGGVLPHINRALLLKVEQKKNKKA  138


>XP_002544485.1| histone H2A variant [Uncinocarpus reesii 1704]
 gb|EEP79156.1| histone H2A variant [Uncinocarpus reesii 1704]
         Length=138

 Score = 218.0 bits (554),  Expect = 1E-70
 Identities = 107/120 (89%), Positives = 113/120 (94%), Gaps = 0/120 (0%)

Query  22   EGANKKQQSHSARAGLQFPCGRVKRFLKQNTQQKMRVGAKAAVYVTAVLEYLTAEVLELA  81
            + A K Q+SHSA+AGLQFPCGR+KRFLK NTQ KMRVGAKAAVYVTAVLEYLTAEVLELA
Sbjct  17   DAAGKTQRSHSAKAGLQFPCGRIKRFLKNNTQNKMRVGAKAAVYVTAVLEYLTAEVLELA  76

Query  82   GNAAKDLKVKRITPRHLQLAIRGDEELDTLIRATIAYGGVLPHINRALLLKVEQKKKAKA  141
            GNAAKDLKVKRITPRHLQLAIRGDEELDTLIRATIA+GGVLP INRALLLKVEQKKK K+
Sbjct  77   GNAAKDLKVKRITPRHLQLAIRGDEELDTLIRATIAFGGVLPRINRALLLKVEQKKKGKS  136


>XP_013326979.1| Histone H2A [Rasamsonia emersonii CBS 393.64]
 gb|KKA20367.1| Histone H2A [Rasamsonia emersonii CBS 393.64]
         Length=138
```

```
 Score = 218.0 bits (554),  Expect = 1E-70
 Identities = 110/123 (89%), Positives = 115/123 (93%), Gaps = 1/123 (1%)

Query  22   EGANKKQQSHSARAGLQFPCGRVKRFLKQNTQQKMRVGAKAAVYVTAVLEYLTAEVLELA  81
            + A K Q+SHSA+AGLQFPCGRVKRFLK NTQ KMRVGAKAAVYVTAVLEYLTAEVLELA
Sbjct  17   DAAGKTQKSHSAKAGLQFPCGRVKRFLKNNTQNKMRVGAKAAVYVTAVLEYLTAEVLELA  76

Query  82   GNAAKDLKVKRITPRHLQLAIRGDEELDTLIRATIAYGGVLPHINRALLLKVEQKKKAKA  141
            GNAAKDLKVKRITPRHLQLAIRGDEELDTLIRATIA+GGVLP INRALLLKVEQKKK K
Sbjct  77   GNAAKDLKVKRITPRHLQLAIRGDEELDTLIRATIAFGGVLPRINRALLLKVEQKKKNK-  135

Query  142  LEG  144
            +EG
Sbjct  136  VEG  137


>XP_028481541.1| histone-fold-containing protein [Byssochlamys spectabilis]
 gb|RWQ91896.1| histone-fold-containing protein [Byssochlamys spectabilis]
 dbj|GAD92097.1| histone H2A.Z [Byssochlamys spectabilis No. 5]
          Length=138

 Score = 218.0 bits (554),  Expect = 1E-70
 Identities = 108/119 (90%), Positives = 112/119 (94%), Gaps = 0/119 (0%)

Query  22   EGANKKQQSHSARAGLQFPCGRVKRFLKQNTQQKMRVGAKAAVYVTAVLEYLTAEVLELA  81
            + A K Q+SHSA+AGLQFPCGRVKRFLK NTQ KMRVGAKAAVYVTAVLEYLTAEVLELA
Sbjct  17   DAAGKTQKSHSAKAGLQFPCGRVKRFLKNNTQNKMRVGAKAAVYVTAVLEYLTAEVLELA  76

Query  82   GNAAKDLKVKRITPRHLQLAIRGDEELDTLIRATIAYGGVLPHINRALLLKVEQKKKAK  140
            GNAAKDLKVKRITPRHLQLAIRGDEELDTLIRATIA+GGVLP INRALLLKVEQKKK K
Sbjct  77   GNAAKDLKVKRITPRHLQLAIRGDEELDTLIRATIAFGGVLPRINRALLLKVEQKKKNK  135
```
