# Supplementary figures and images for: Effect of H2A.Z deletion is rescued by compensatory mutations in Fusarium graminearum
Source: PLoS Genet. 2020 Oct 22;16(10):e1009125. doi: 10.1371/journal.pgen.1009125 (PMC7608984; doi:10.1371/journal.pgen.1009125)

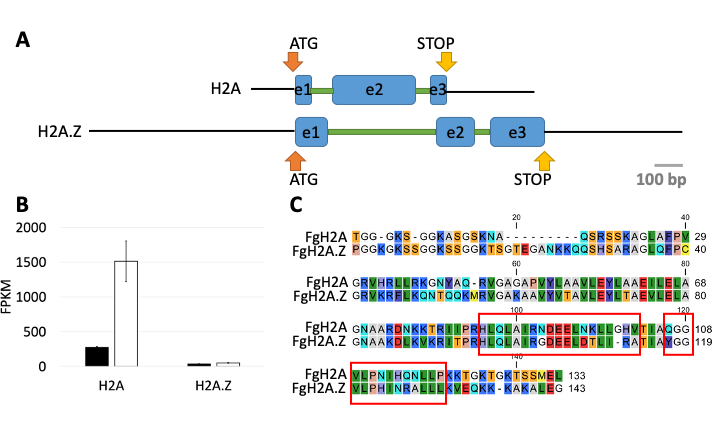

Supplement: S1 Fig — (A) Genomic sequences. Blue boxes are exons (e); green boxes are introns; black lines are UTRs. Translation start (ATG) and stop sites are indicated with orange and yellow arrows, respectively. Scaling key is provided in grey. (B). FgH2A and FgH2A.Z expression levels (in FPKM) previously measured by Zhao and Colleagues [1] in asexual spores (black bars) or actively growing mycelium (white bars). Displayed values are means of three replicates and error bars are standard deviations. (C). Protein alignment of FgH2A and FgH2A.Z. Residues were colored according to Rasmol scheme. Red boxes indicate domains essential for H2A.Z function (vs. H2A) according to Suto et al. [2]. All datasets can be viewed at FungiDB [3,4] (see ‘Additional References’ in S1 Text). (TIFF) [file pgen.1009125.s004.tiff]

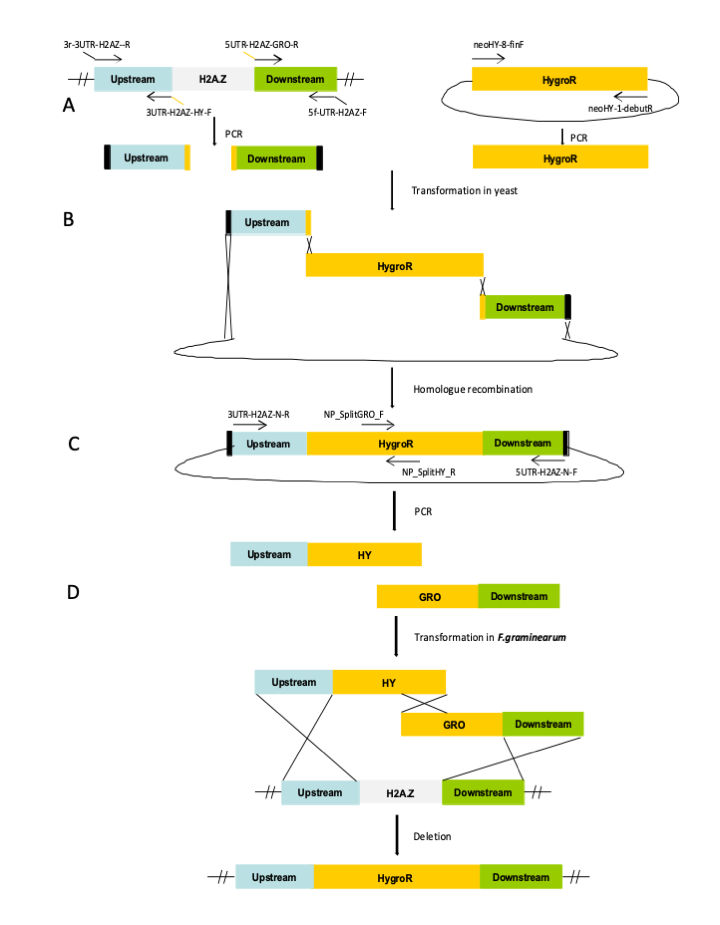

Supplement: S2 Fig — (A) The upstream and downstream flanking regions of H2A.Z, HygroR cassette were amplified from wild type F. graminearum genomic DNA and plasmid DNA of pBlueScriptSK(-)_NeoHygroR, respectively. (B) Fragment assembly in yeast. The three fragments were assembled by plasmid pRS426. (C) Extraction of yeast genomic DNA and amplification of fragments for split-marker method in F. graminearum. (D) Transformation in F. graminearum. H2A.Z was replaced by HygroR cassette. (TIFF) [file pgen.1009125.s005.tiff]

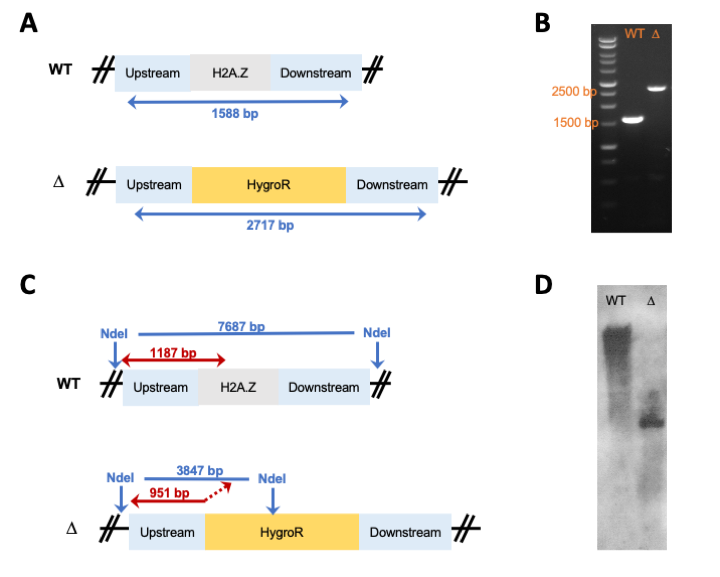

Supplement: S3 Fig — (A) Schematic representation of the loci amplified. Primers matching the upstream and downstream flanking regions of H2A.Z were used to amplify a 1,588 bp- or 2,717 bp-long fragments from wild-type INRA349 gDNA or ΔH2A.Z mutant gDNA, respectively. (B) Result of the agarose gel electrophoresis of the fragments obtained from S3A Fig. Lane 1: ladder; lane 2: INRA349 WT; lane 3: I349ΔH2A.Z. (C) Schematic representation of the Southern blot strategy used. Digestion with NdeI of gDNA extracted from I349 wild-type or ΔH2A.Z leads to fragments of 7,687 bp and 3,847 bp in size, respectively. A probe marked with digoxygenin and matching the upstream region of H2A.Z CDS as well as part of its 5’ end was then synthesized and used to reveal the two digestion fragments obtained on X-ray films (as displayed in (D); Lane 1: INRA349 WT; lane 2: I349ΔH2A.Z. WT = INRA349 WT; Δ = I349ΔH2A.Z. (TIFF) [file pgen.1009125.s006.tiff]

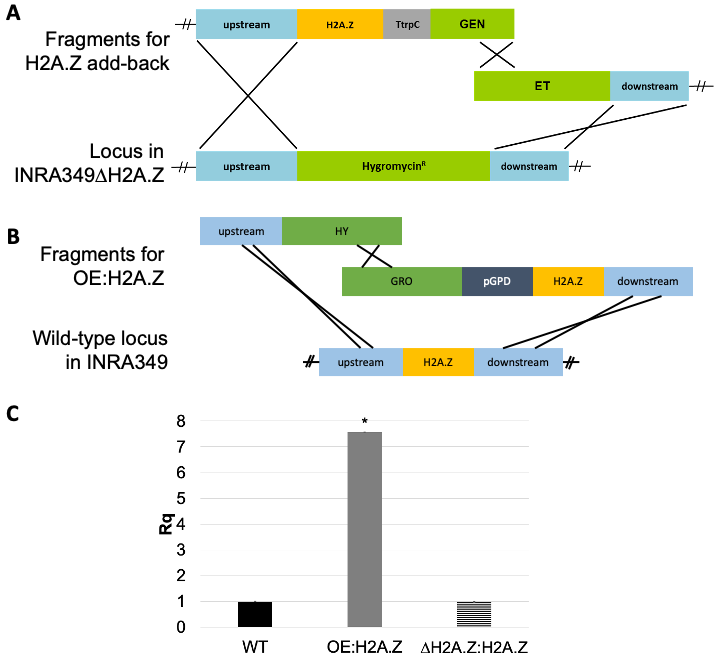

Supplement: S4 Fig — Schematic representation of the split-marker strategy used to add back H2A.Z to INRA349ΔH2A.Z at its original locus (A), or place it under the control of the strong constitutive promoter pGPD (B). (C) Rq expression levels for H2A.Z in INRA349OE:H2A.Z (grey bar; Rq = 7.6) and INRA349ΔH2A.Z (stripped bar; Rq = 0.978) relative to wild type (black bar; Rq = 1), measured by RT-qPCR. Displayed values are means of three replicates, error bars are standard deviations. The star indicates significant difference with p < 0.01. (TIFF) [file pgen.1009125.s007.tiff]

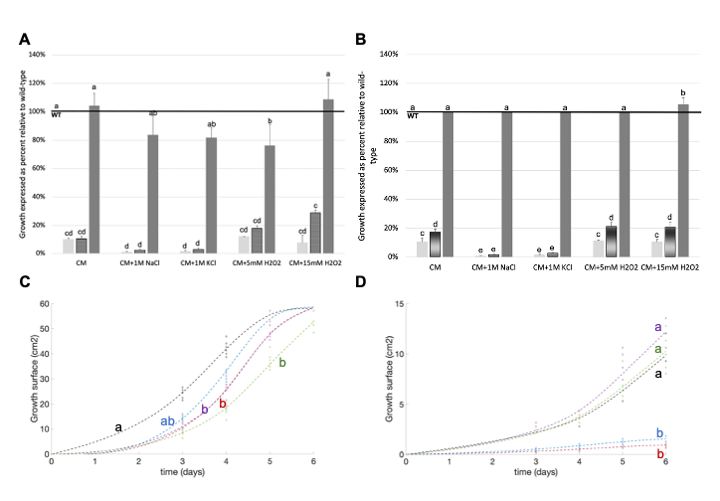

Supplement: S5 Fig — (A) 3 dpi and (B) 6 dpi radial growth in I349ΔH2A.Z (light grey), I349ΔH2A.Z::H2A.Z (stripes), and I349OE::H2A.Z (dark grey) expressed relative to growth of wild-type in not supplemented CM medium (black solid line marking 100% of growth). (C) and (D) Fitted radial growth kinetics followed for six days for I349 OE:H2A.Z (C) and I349ΔH2A.Z::H2A.Z (D). Black = CM; red = CM + NaCl 1M; blue = CM + KCl 1M; purple = CM + H2O2 5 mM; green = CM + H2O2 15 mM. In all panels, letters indicate statistically significant curve groups after Kruskal-Wallis testing and Tukey-Kramer correction for multiple testing (p < 0.05). (TIFF) [file pgen.1009125.s008.tiff]

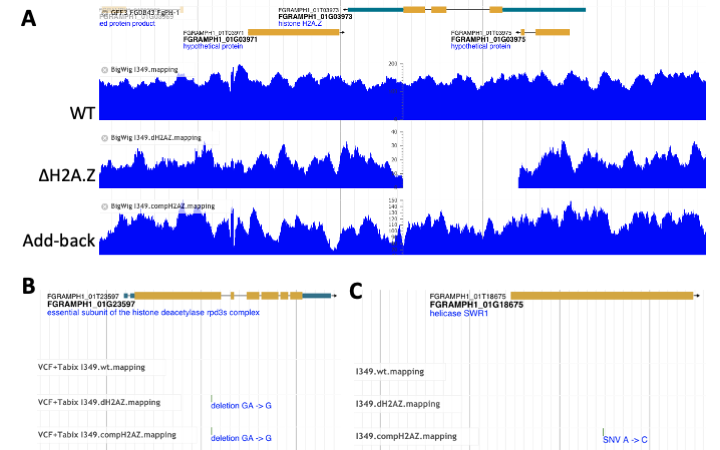

Supplement: S6 Fig — (A) JBrowse screenshot of read coverages per base. (B) and (C) Mutations detected elsewhere in the genomes of I349Δ:H2A.Z (B) and I349ΔH2A.Z::H2A.Z (C). (TIFF) [file pgen.1009125.s009.tiff]

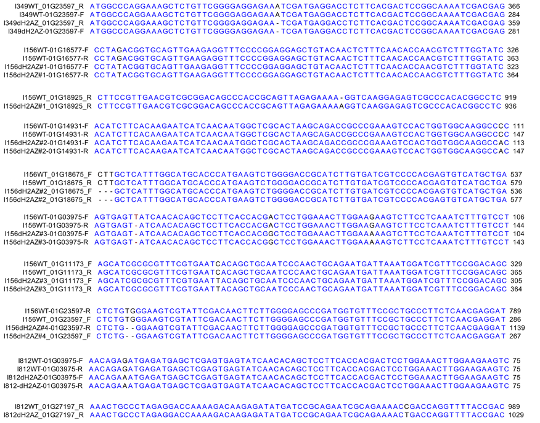

Supplement: S7 Fig — Nucleotides in black are nucleotides that are different. (TIFF) [file pgen.1009125.s010.tiff]

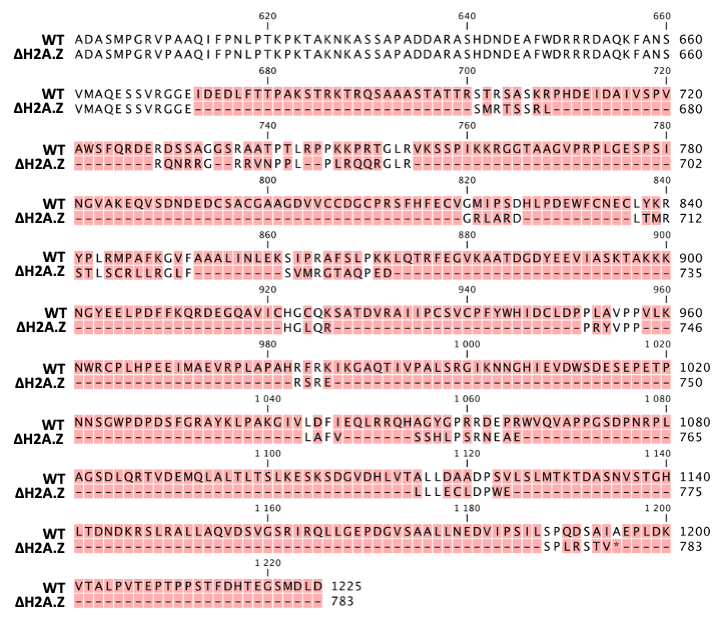

Supplement: S8 Fig — Only the C-terminal end of the protein, containing the mutation is depicted. Pink boxes highlight differences between sequences. (TIFF) [file pgen.1009125.s011.tiff]

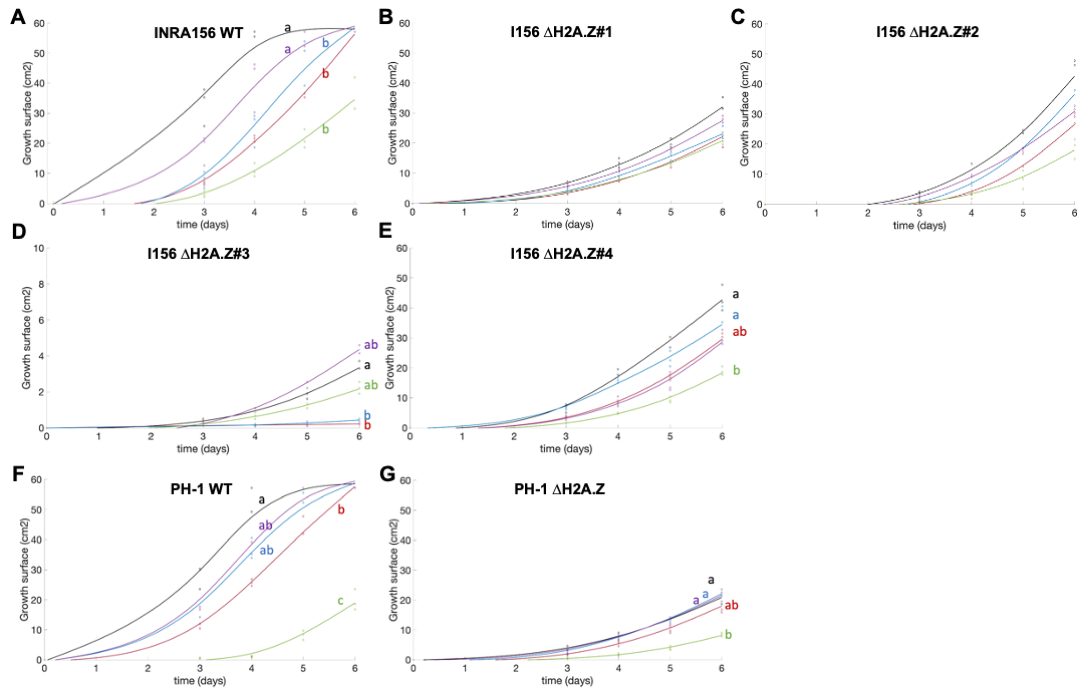

Supplement: S9 Fig — Fitted radial growth kinetics followed for six days for INRA156 wild-type (A) and its four ΔH2A.Z mutants (B) to (E), and PH-1 wild-type (F) and its ΔH2A.Z mutant (G). Black = CM; red = CM + NaCl 1M; blue = CM + KCl 1M; purple = CM + H2O2 5 mM; green = CM + H2O2 15 mM. Letters indicate statistically significant curve groups after Kruskal-Wallis testing and Tukey-Kramer correction for multiple testing (p < 0.05). (TIFF) [file pgen.1009125.s012.tiff]

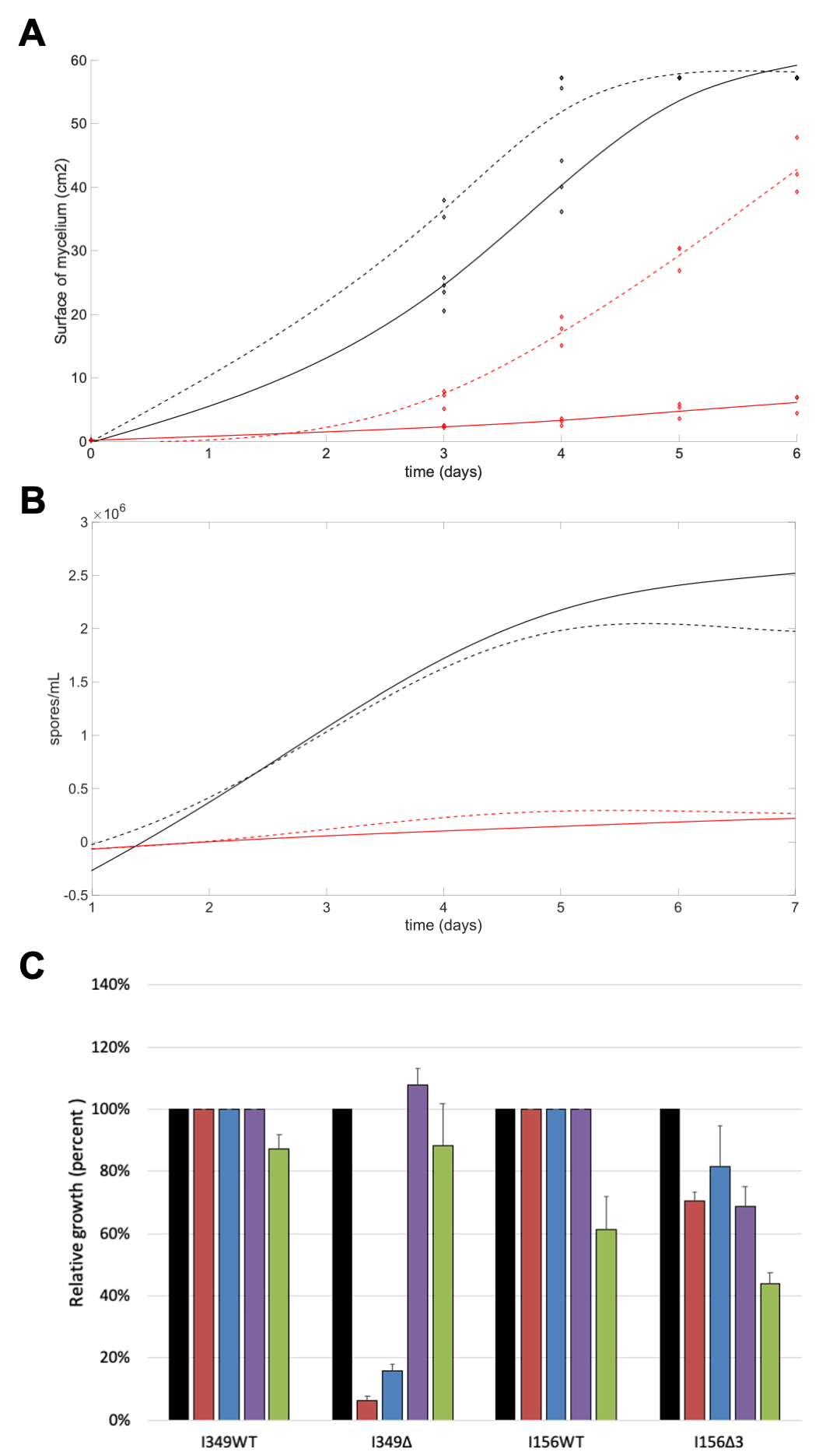

Supplement: S10 Fig — (A) Fitted radial growth kinetics followed for six days. (B) Fitted kinetics of sporulation. (A) and (B) Solid black = wild-type I349; dashed black = wild-type I156; solid red = I349ΔH2A.Z; dashed red = I156ΔH2A.Z#4. (C) Effect of NaCl (red bars), KCl (blue bars), H2O2 5 mM (purple bars), and H2O2 15 mM (green bars) after 6 day-long radial growth of INRA349 wild-type (I349WT), INRA156 wild-type (I156WT), and their deletion mutants I349ΔH2A.Z (I349Δ) and I156ΔH2A.Z#4 (I156Δ4). For comparison sake, growth of a given strain is expressed in percentage relative to itself grown in not supplemented CM medium (black bars). (TIFF) [file pgen.1009125.s013.tiff]

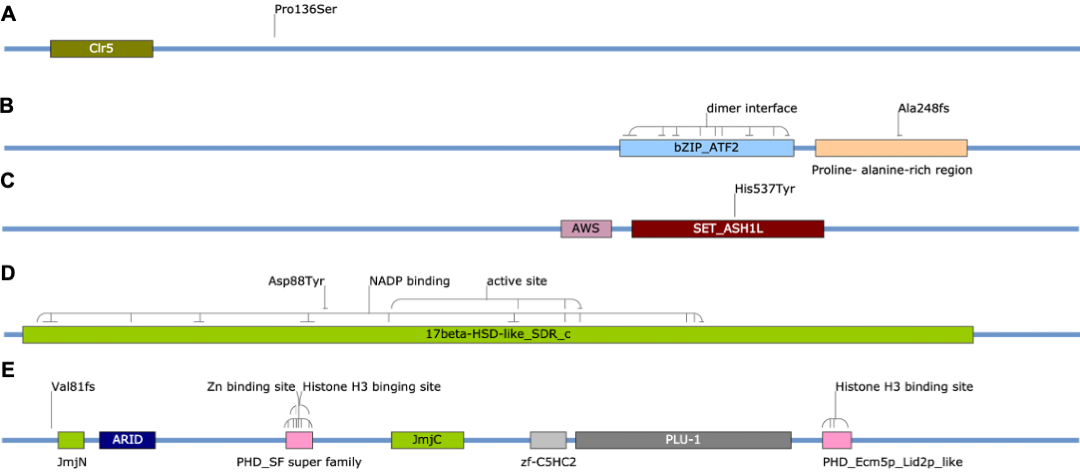

Supplement: S11 Fig — (A) Protein of unknown function (FGRAMPH1_01G26683; 537 aa) containing a Clr5 domain (PFAM14420). “Pro136Ser” indicates a mutation. (B) Transcription factor (FGRAMPH1_01G26173; 297 aa) containing a bZIP_ATF2 domain (CL21462). “Ala248fs” indicates a mutation. (C) SET2 protein (FGRAMPH1_01G11173; 786 aa) containing a SET_ASH1L domain (CL1917) and an AWS domain (PFAM17907). “His537Tyr” indicates a mutation. (D) Yusz oxidoreductase (FGRAMPH1_01G16577; 292 aa) containing a 17beta-HSD-like_SDR_c domain (CD05374). “Asp88Tyr” indicates a mutation. (E) Jarid1 histone demethylase (FGRAMPH1_01G18925; 1,731 aa) containing various domains (PLU-1 PFAM08429, JmjC PFAM02373, ARID SMART01014, PHD_Ecm5p_Lid2p_like CD15518, JmjN SMART00545, PHD_SF super family CL22851, zf-C5HC2 PFAM02928). “Val81fs” indicates a mutation. Architectures are displayed with SnapGene Viewer 5.0.6. (TIFF) [file pgen.1009125.s014.tiff]

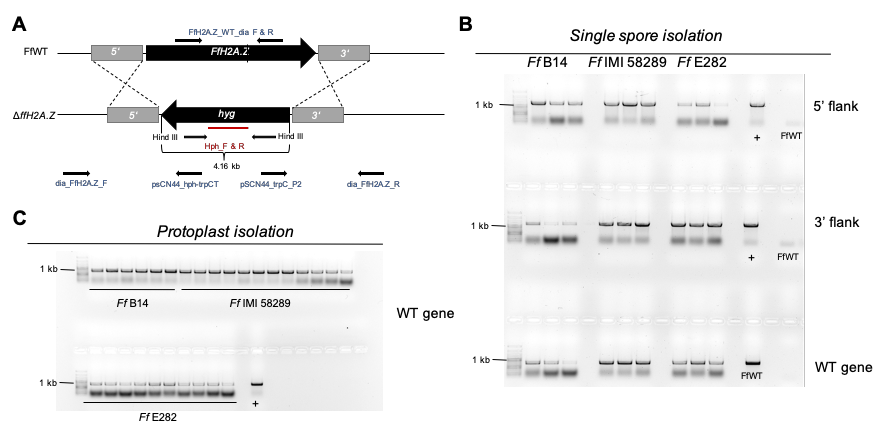

Supplement: S12 Fig — (A) Schematic representation of the deletion strategy and position of the primers used for PCR validation (B). Visualization of typical PCR results after three rounds of single-spore isolation. + = ΔffH2A.Z gDNA before single spore isolation; FfWT = F. fujikuroi wild-type gDNA. Results are shown for three transformants per strain tested (C) Visualization of PCR results after protoplast isolation. + = ΔffH2A.Z gDNA before protoplast isolation. (TIFF) [file pgen.1009125.s015.tiff]
